# Supplementary material for: Retroviral integration into nucleosomes through DNA looping and sliding along the histone octamer
Source: Nat Commun. 2019 Sep 13;10:4189. doi: 10.1038/s41467-019-12007-w (PMC6744463; doi:10.1038/s41467-019-12007-w)
Supplement: Supplementary file 1 — Supplementary Information [file 41467_2019_12007_MOESM1_ESM.pdf]

Supplementary Information

Retroviral integration into nucleosomes through DNA looping and sliding along the histone octamer

M. D. Wilson, L. Renault et al.

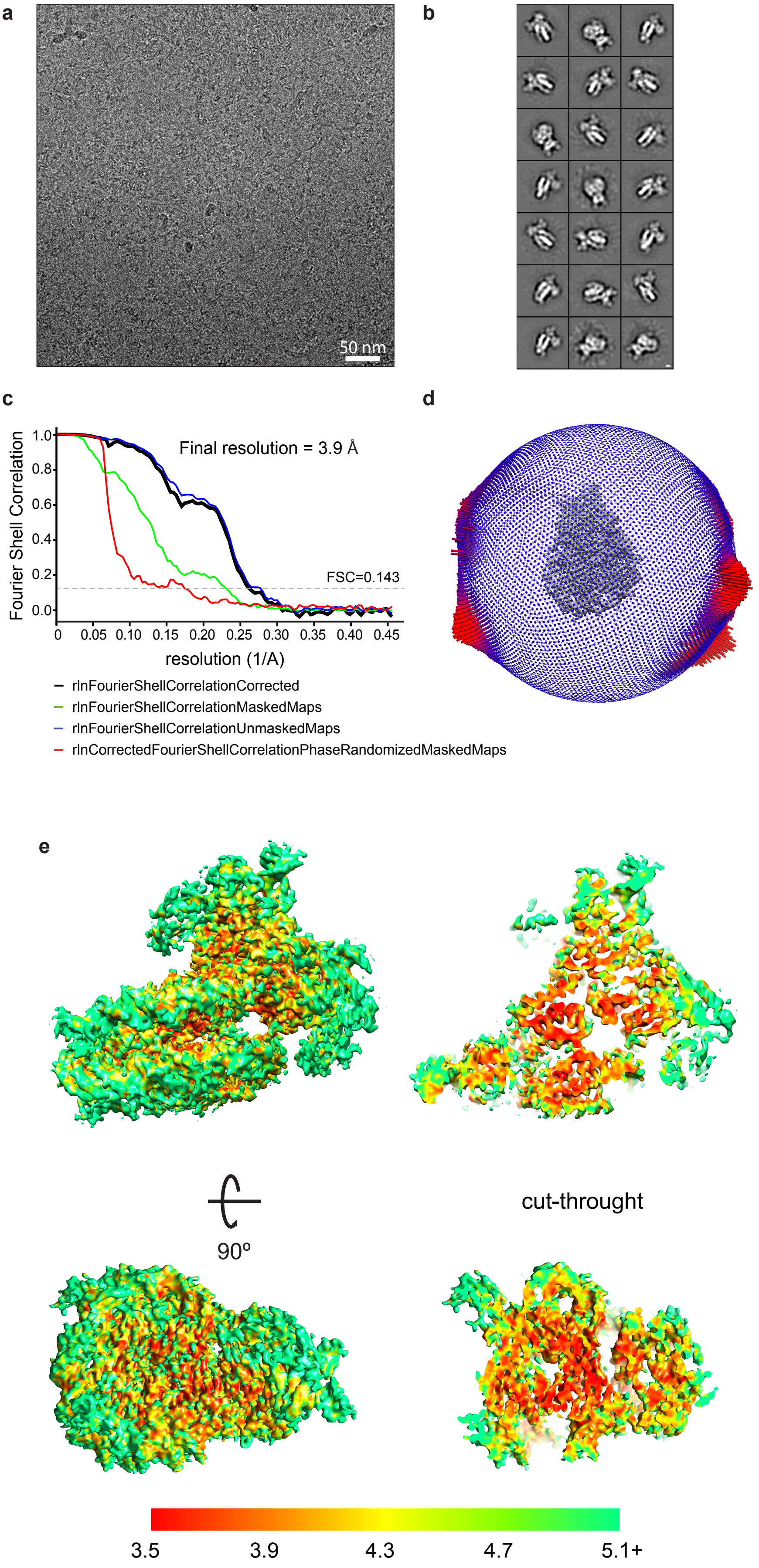
a
50 nm
b
c
Final resolution = 3.9 Å
Fourier Shell Correlation
1.0
0.8
0.6
0.4
0.2
0.0
FSC=0.143
0
0.05
0.10
0.15
0.20
0.25
0.30
0.35
0.40
0.45
resolution (1/A)
rlnFourierShellCorrelationCorrected
rlnFourierShellCorrelationMaskedMaps
rlnFourierShellCorrelationUnmaskedMaps
rlnCorrectedFourierShellCorrelationPhaseRandomizedMaskedMaps
d
e
90º
cut-throught
3.5
3.9
4.3
4.7
5.1+

Supplementary Figure 1 Cryo-EM analysis of Intasome-D02 NCP. **(a)** Representative micrograph. **(b)** Representative 2D averages, white scale bar is 25 Angstrom long. **(c)** Gold standard FSC curve for the refined cryo-EM map. **(d)** Euler angle distribution plot for all particles included in the final map. Bar length and colour (blue low, red high) correspond to number of particles contributing to each view. **(e)** Cryo-EM map coloured according to local resolution estimated with ResMap.

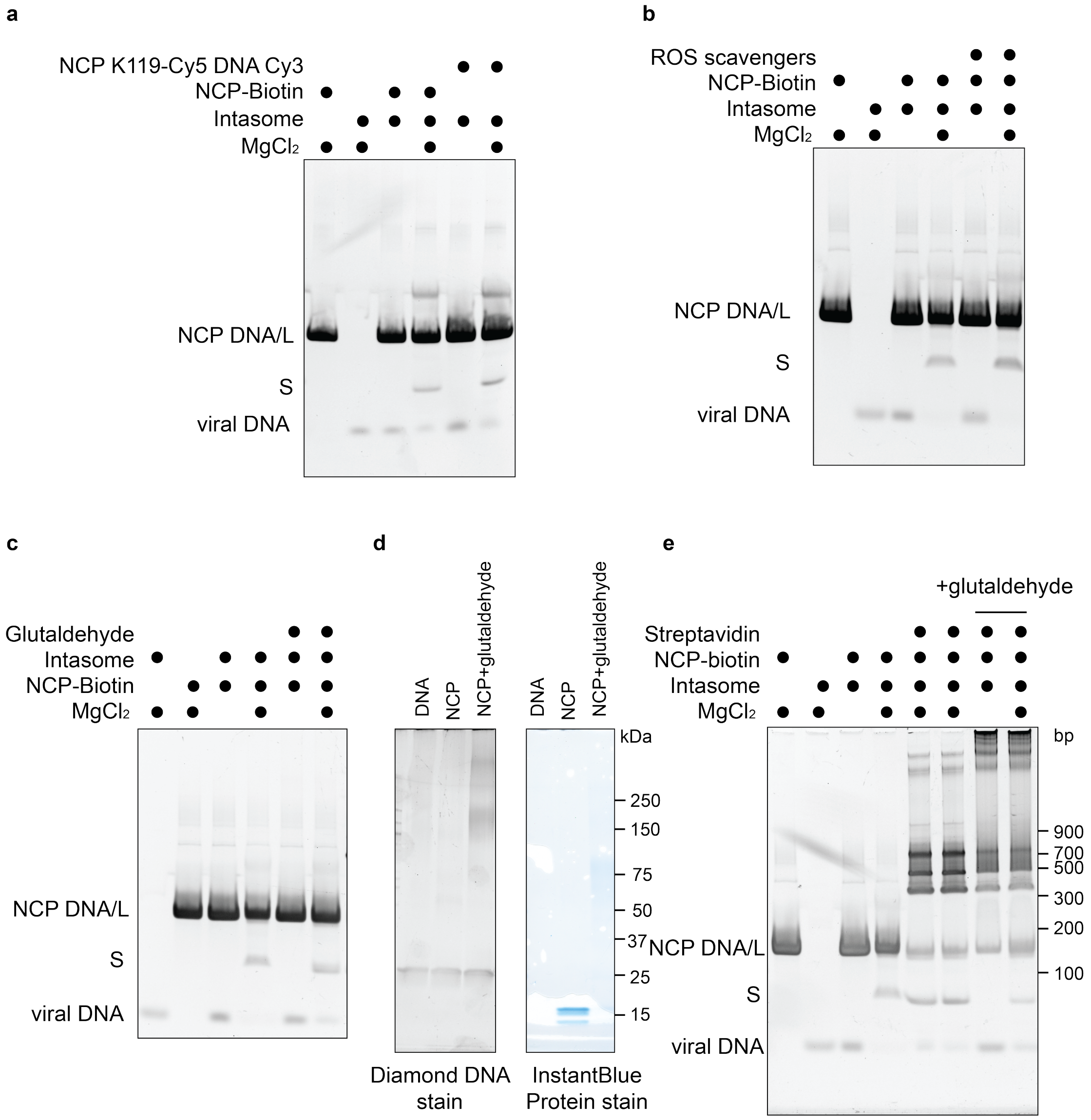

Supplementary Figure 2 Retroviral integration into various nucleosome derivatives. **(a)** Integration assay shows magnesium dependence of viral strand transfer. Strand transfer product S and L labeled. Assay performed with D02-NCP used for structure determination and fluorophore labelled nucleosomes used in single-molecule FRET experiments. **(b)** PFV Integration assay shows that ROS scavengers used in single-molecule FRET experiments do not affect the strand transfer reaction. **(c)** Glutaraldehyde cross-linking of D02 NCP does not prevent integration. **(d)** SDS-PAGE gels stained with Diamond nucleic acid stain (left) or InstantBlue protein stain (right). Glutaraldhyde cross-inked sample migrates as lower electrophoretic-mobility smear. **(e)** Efficient integration occurs with NCP-D02-biotin complexes pre-assembled with streptavidin, and cross-linked with glutaraldehyde.

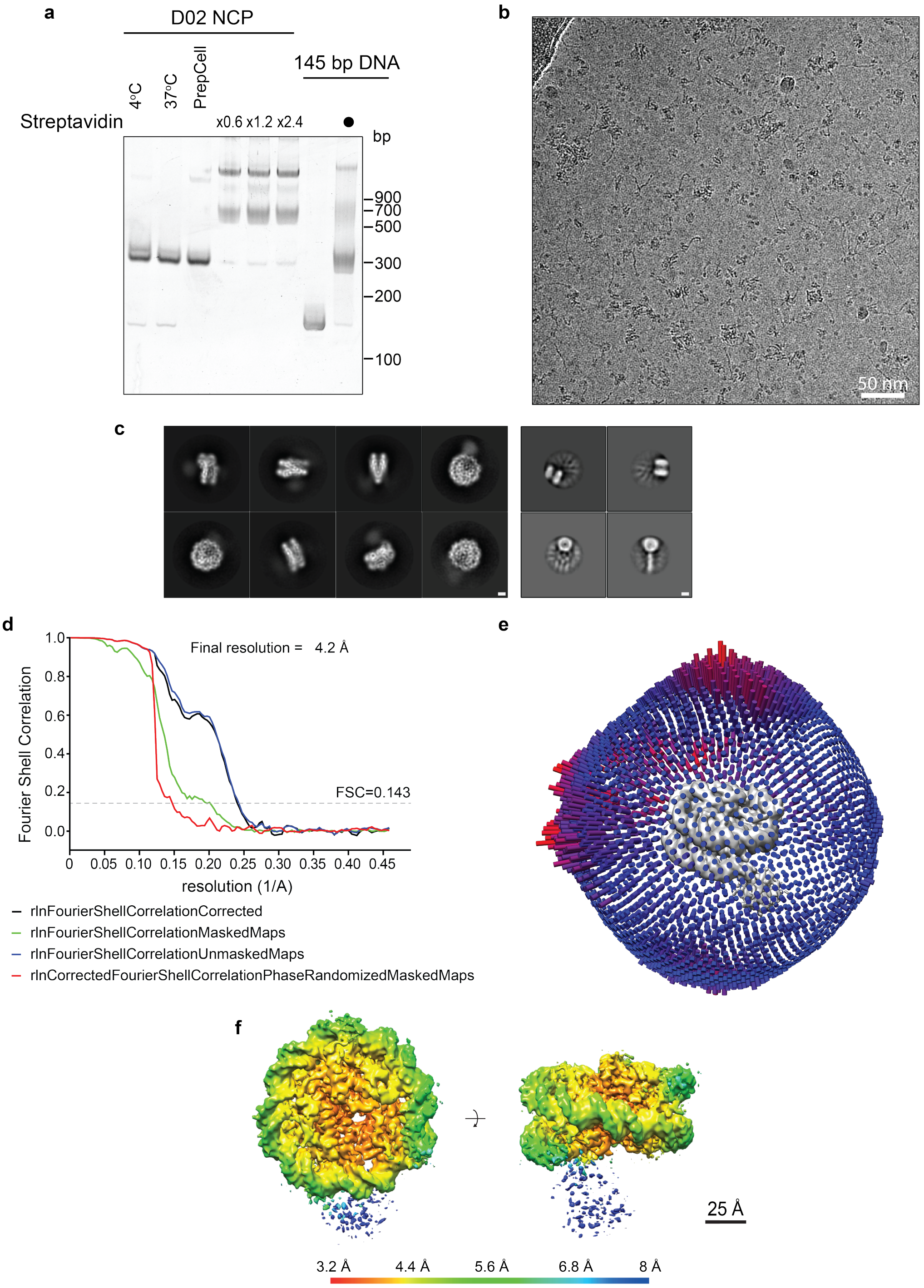

Supplementary Figure 3 D02 NCP-streptavidin preparation and cryo-EM characterisation. **(a)** Native gel electrophoresis of biotin-D02 wrapped NCP during purification and interaction with streptavidin. **(b)** Representative cryo-EM micrograph of NCP-D02-streptavidin. **(c)** Selected 2D averages of D02 NCP-streptavidin (left) and isolated or DNA-bound streptavidin (right). , white scale bar is 25 Angstrom long. **(d)** Gold standard FSC curve for the final structure. (**e**) Euler angle distribution plot for all particles included in the final map. Bar length and colour (blue low, red high) correspond to number of particles contributing to each view. (**f**) NCP-D02-streptavidin coloured according to local resolution estimated in RELION. Streptavidin density is fragmented at the displayed threshold, due to the flexible tether with DNA.

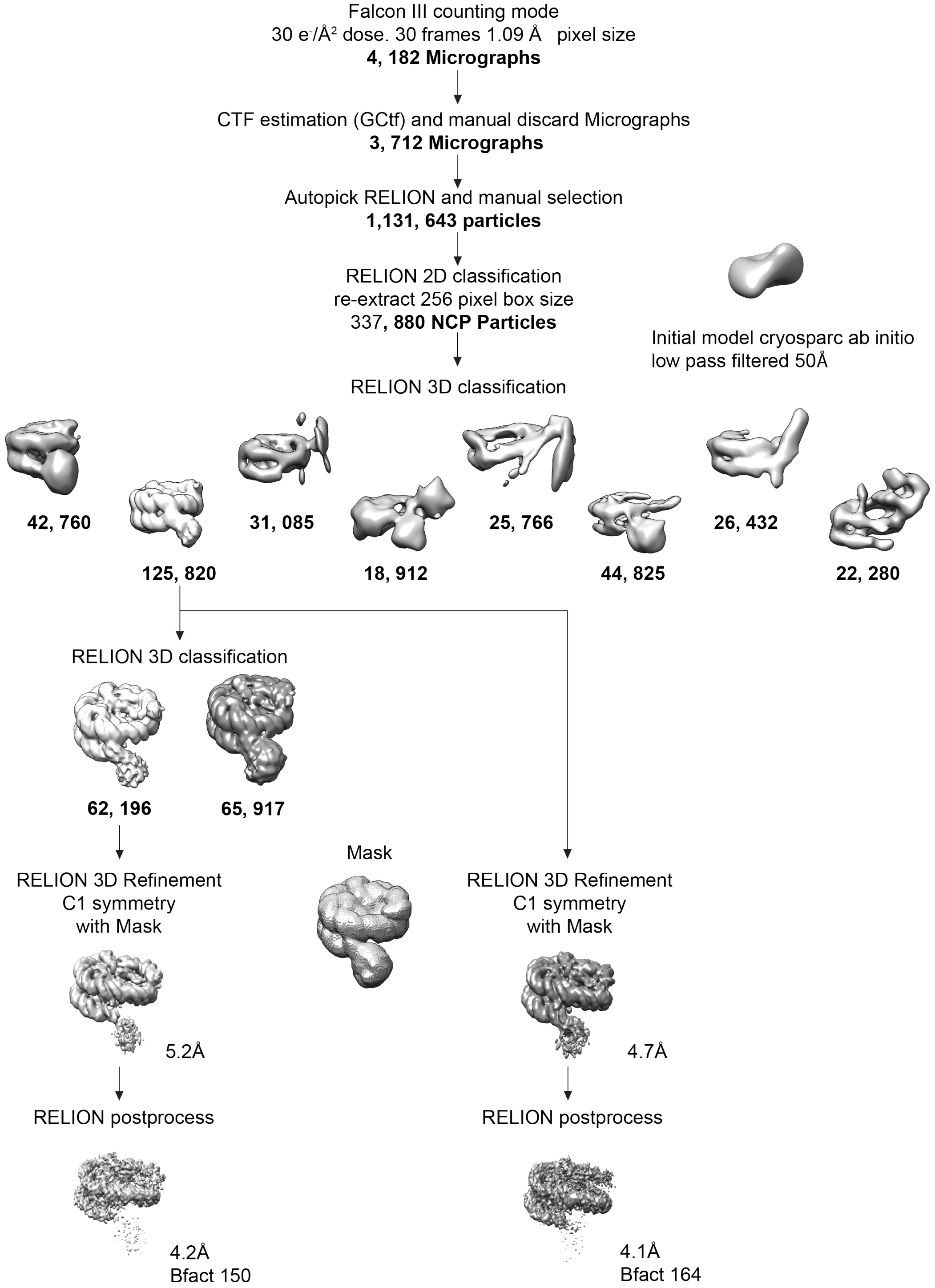

Supplementary Figure 4 Overview of cryo-EM single-particle reconstruction for D02 NCP-streptavidin.

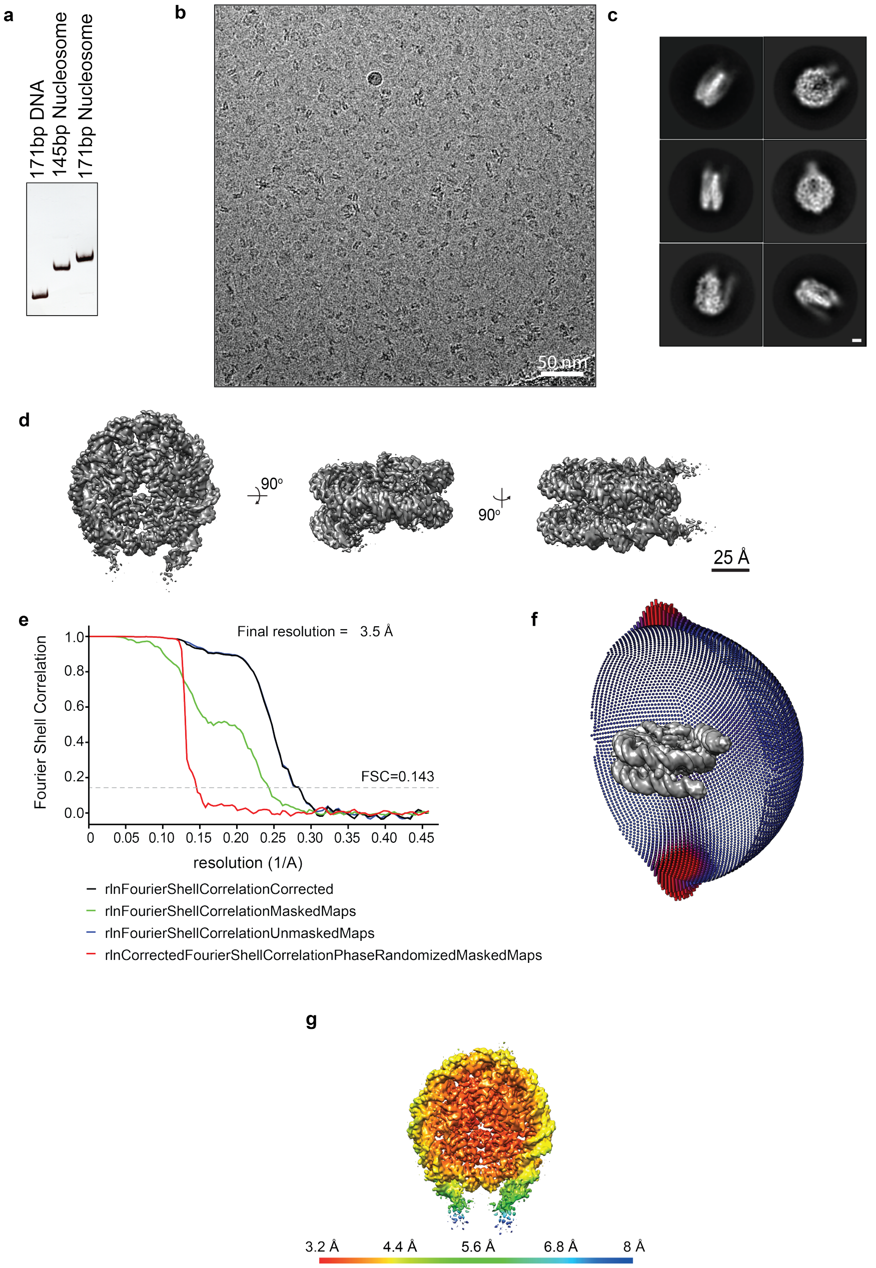

Supplementary Figure 5 Electron microscopy of Widom 601 NCP. **(A)** Native gel electrophoresis of Widom 601 DNA isolated, and wrapped in a nucleosomes with either 145 base pair or 171 base pair-long DNA. **(B)** Representative cryo micrograph of nucleosome reconstituted with 171bp of Widom 601 DNA. **(C)** Selected representative 2D class averages, ,white scale bar is 25 Angstrom long. **(D)** Surface rendering of the NCP 171bp complex, shown in three views rotated about their axis. **(E)** Gold standard FSC curve for the refined cryo-EM map. **(F)** Euler angle distribution plot of all particles used for the final map (C2 symmetry imposed. Bar length and colour (blue low, red high) corresponds to number of particle images contributed to each view. **(G)** 171-base pair Widom 601 nucleosome, coloured according to local resolution estimated in RELION. Density for the DNA projecting away from the NCP core is visibly weaker.

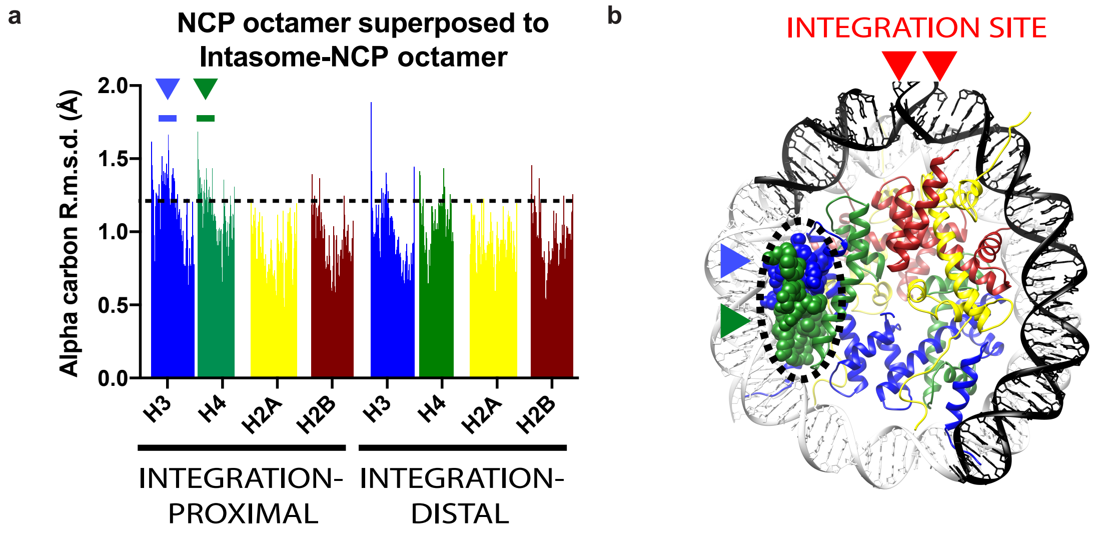

Supplementary Figure 6 Intasome-induced octamer distortion. **(a)** After superposing Intasome-free and intasome-engaged D02 NCP via the histone octamers, the alpha carbon R.M.S.D. was measured for each histone chain. The most prominent intasome-induced movement is observed for histones H3 and H4 mapping on the nucleosome face proximal to the integration site. **(b)** Inspection of the integration-proximal face of the nucleosome shows that the most prominent movement clusters around DNA-interacting histone H3 and H4 elements, at a site downstream of histone H3 L1. Dotted line, blue and green triangles and atom spheres represent the region of most prominent alpha carbon movement. Red triangles indicate the integration site. Register-shifted DNA is black. Unperturbed DNA is white.

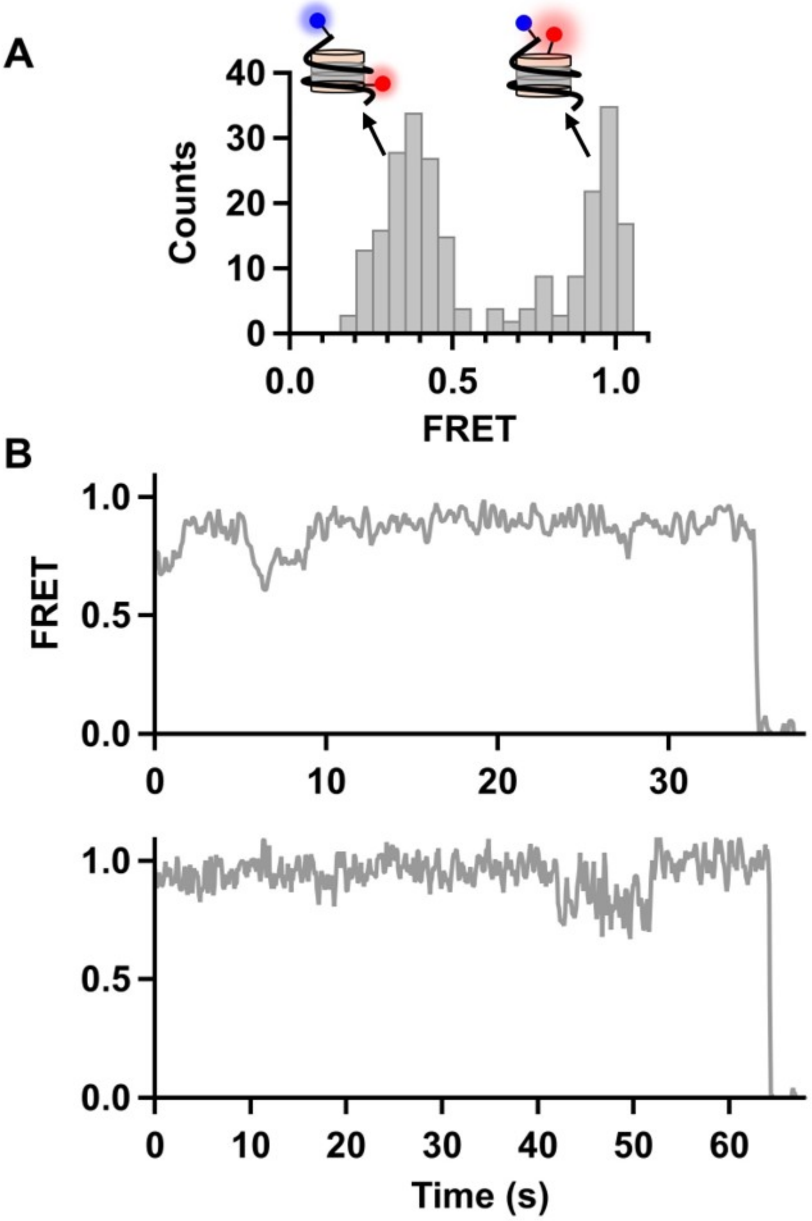

Supplementary Figure 7. Single-molecule FRET of D02 nucleosome core particles (A) Single-molecule FRET histogram of labelled nucleosomes (N = 245). Distal- and proximal-labelled nucleosomes yield 0.37 and 0.95 FRET-efficiency peaks, respectively. **(B)** Representative dynamic single-molecule FRET time trajectories of proximal-only fluorescent nucleosomes. Data were collected at 100 ms/frame and smoothed with a 3-point moving average.

mdw100	tggaacacaTGCACAGGATGTATATATCTGAC
mdw101	tggaacacaCCCTGGAGAATCCCGGTGC
mdw171	GGCTGTGTTTGTATCAAGTTACCTG
mdw166-2	/5Cy3/TGTCCAGGTTCTCCCTGTGGTGAAAACC
mdw155	TGTCCAGGTTCTCCCTGTGGTGAAAACC
mdw153	/5Biosg/GGCTGTGTTTGTATCAAGTTACCTG
mdw1	GTGATGGCGCTGCAGGAGGCTagcGAGGCCTACCTGGTGGGGCtatt
mdw2	GCCCCACCAGGTAGGCCTCgctAGCCTCCTGCAGCGCCATCACcgc
mdw3	GGTGGGGCTATTTGAGGATACCAACCTGgccGCCATTCACGCCAAACGCGTCAC
mdw4	GTGACGCGTTTGGCGTGAATGGCggcCAGGTTGGTATCCTCAAATAGCCCCACC
mdw25	GAATATCCAGGCTGTTCTGCTGCCGAAAtgcACCGAATCTCACCACAAAGCGAAAGG
mdw26	CCTTTCGCTTTGTGGTGAGATTCGGTgcaTTTCGGCAGCAGAACAGCCTGGATATTC
IO1	TGCGAAATTCCATGACA
IO2	ATTGTCATGGAATTTCGCA

Supplemantary Table 1 List of oligos.
