# Supplementary material for: Retroviral integration into nucleosomes through DNA looping and sliding along the histone octamer
Source: Nat Commun. 2019 Sep 13;10:4189. doi: 10.1038/s41467-019-12007-w (PMC6744463; doi:10.1038/s41467-019-12007-w)
Supplement: Supplementary file 3 — Description of Additional Supplementary Files [file 41467_2019_12007_MOESM3_ESM.pdf]

Descriptions of Additional Supplementary Files

File name: Supplementary Movie 1

Description: Nucleosome core particle morphed between the isolated and the strand transfer complex state.
