# Supplementary material for: Retroviral integration into nucleosomes through DNA looping and sliding along the histone octamer
Source: Nat Commun. 2019 Sep 13;10:4189. doi: 10.1038/s41467-019-12007-w (PMC6744463; doi:10.1038/s41467-019-12007-w)
Supplement: Supplementary file 6 — Source Data [file 41467_2019_12007_MOESM6_ESM.pdf]

Figure 2B Coomassie Pull-down assay

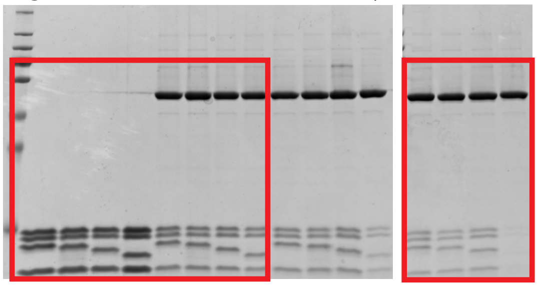

Figure 2C Coomassie Pull-down assay

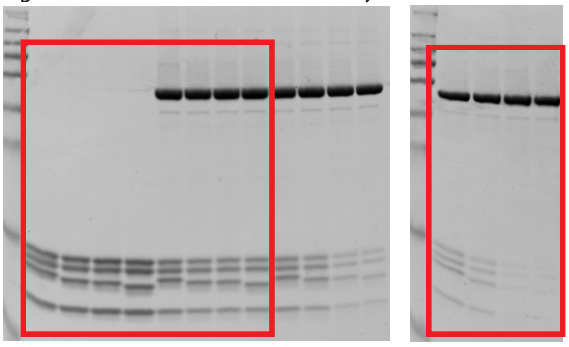

Supp Figure 2A DNA stained integration

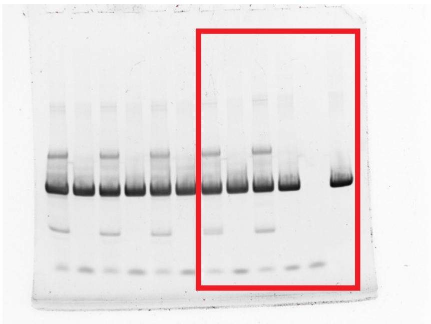

Supp Figure 2B DNA stained integration assay

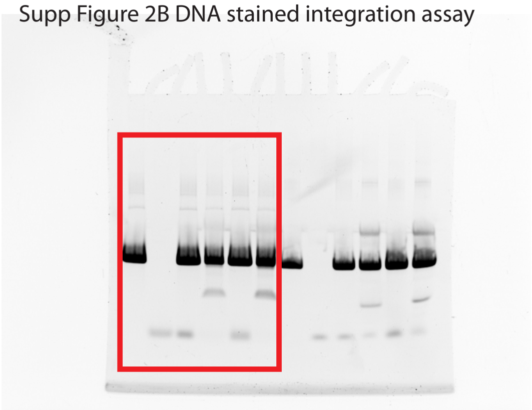

Supp Figure 2c DNA stained integration assay

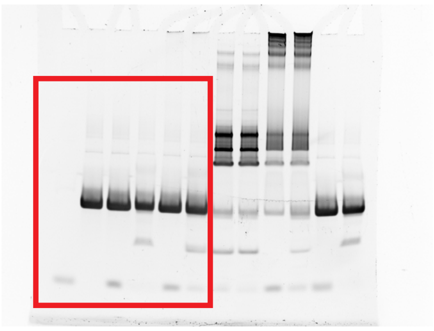

Supp Figure 2D DNA stained and coomassie stained fixation test

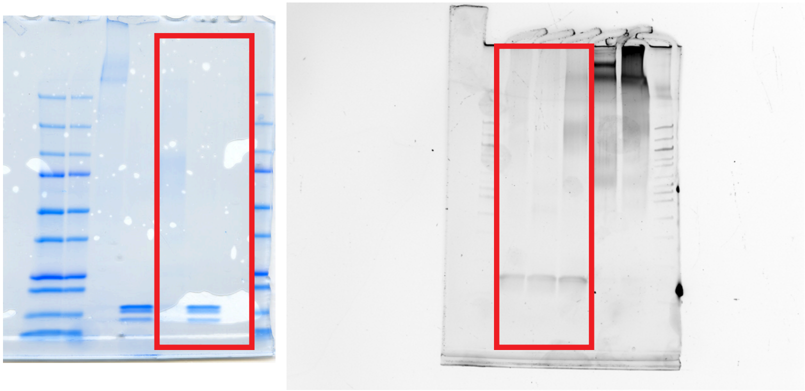

Supp Figure 2E DNA stained integration assay

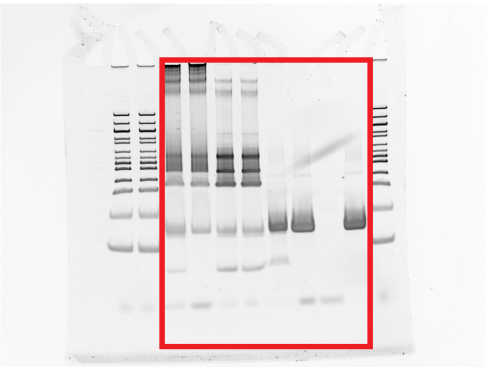

Supp Figure 3A DNA stained native gel

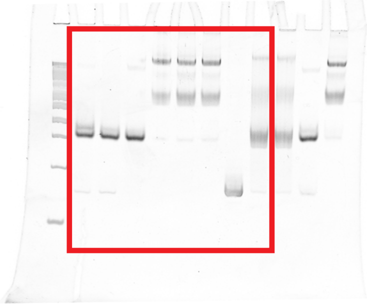

Supp Figure 5A DNA stained native gel

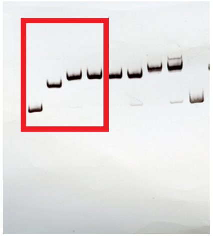

used Superpose in CCP4 - raw data below:

```
RESIDUE ALIGNMENT
 ~~~~~~~~~~~~~~~~~
$$

.-------------.------------.-------------.
|    Query    |  Dist.(A)  |   Target    |
|-------------+------------+-------------|
| + A:HIS  39 |            | + A:HIS  39 |
| + A:ARG  40 | <**1.63**> | + A:ARG  40 |
| . A:TYR  41 | <**1.47**> | . A:TYR  41 |
| + A:ARG  42 | <**1.37**> | + A:ARG  42 |
| + A:PRO  43 | <**1.04**> | + A:PRO  43 |
| - A:GLY  44 | <**1.30**> | - A:GLY  44 |
| . A:THR  45 | <**0.99**> |H. A:THR  45 |
|H- A:VAL  46 | <**0.77**> |H- A:VAL  46 |
|H- A:ALA  47 | <**0.81**> |H- A:ALA  47 |
|H- A:LEU  48 | <**1.19**> |H- A:LEU  48 |
|H+ A:ARG  49 | <**1.21**> |H+ A:ARG  49 |
|H+ A:GLU  50 | <**1.28**> |H+ A:GLU  50 |
|H- A:ILE  51 | <**1.27**> |H- A:ILE  51 |
|H+ A:ARG  52 | <**1.26**> |H+ A:ARG  52 |
|H+ A:ARG  53 | <**1.24**> |H+ A:ARG  53 |
|H. A:TYR  54 | <**1.24**> |H. A:TYR  54 |
|H+ A:GLN  55 | <**1.26**> | + A:GLN  55 |
|H+ A:LYS  56 | <**1.34**> | + A:LYS  56 |
| . A:SER  57 | <**1.27**> | . A:SER  57 |
| . A:THR  58 | <**1.21**> | . A:THR  58 |
| + A:GLU  59 | <**1.03**> | + A:GLU  59 |
| - A:LEU  60 | <**0.88**> | - A:LEU  60 |
| - A:LEU  61 | <**1.22**> | - A:LEU  61 |
| - A:ILE  62 | <**1.40**> | - A:ILE  62 |
| + A:ARG  63 | <**1.53**> | + A:ARG  63 |
| + A:LYS  64 | <**1.36**> | + A:LYS  64 |
|H- A:LEU  65 | <**1.35**> |H- A:LEU  65 |
|H+ A:PRO  66 | <**1.43**> |H+ A:PRO  66 |
|H- A:PHE  67 | <**1.33**> |H- A:PHE  67 |
|H+ A:GLN  68 | <**1.34**> |H+ A:GLN  68 |
|H+ A:ARG  69 | <**1.41**> |H+ A:ARG  69 |
|H- A:LEU  70 | <**1.42**> |H- A:LEU  70 |
|H- A:VAL  71 | <**1.33**> |H- A:VAL  71 |
|H+ A:ARG  72 | <**1.26**> |H+ A:ARG  72 |
|H+ A:GLU  73 | <**1.48**> |H+ A:GLU  73 |
```

H- A:ILE 74	<**1.41**>	H- A:ILE 74
H- A:ALA 75	<**1.30**>	- A:ALA 75
H+ A:GLN 76	<**1.38**>	+ A:GLN 76
H+ A:ASP 77	<**1.68**>	+ A:ASP 77
H- A:PHE 78	<**1.57**>	- A:PHE 78
+ A:LYS 79	<**1.31**>	+ A:LYS 79
. A:THR 80	<**1.24**>	. A:THR 80
+ A:ASP 81	<**1.18**>	+ A:ASP 81
- A:LEU 82	<**1.20**>	- A:LEU 82
S+ A:ARG 83	<**1.24**>	S+ A:ARG 83
S- A:PHE 84	<**1.28**>	S- A:PHE 84
+ A:GLN 85	<**1.40**>	+ A:GLN 85
. A:SER 86	<**1.41**>	H. A:SER 86
H- A:ALA 87	<**1.45**>	H- A:ALA 87
H- A:ALA 88	<**1.29**>	H- A:ALA 88
H- A:ILE 89	<**1.28**>	H- A:ILE 89
H- A:GLY 90	<**1.38**>	H- A:GLY 90
H- A:ALA 91	<**1.36**>	H- A:ALA 91
H- A:LEU 92	<**1.18**>	H- A:LEU 92
H+ A:GLN 93	<**1.12**>	H+ A:GLN 93
H+ A:GLU 94	<**1.24**>	H+ A:GLU 94
H- A:ALA 95	<**1.10**>	H- A:ALA 95
H. A:SER 96	<**1.03**>	H. A:SER 96
H+ A:GLU 97	<**1.16**>	H+ A:GLU 97
H- A:ALA 98	<**1.27**>	H- A:ALA 98
H. A:TYR 99	<**1.07**>	H. A:TYR 99
H- A:LEU 100	<**1.13**>	H- A:LEU 100
H- A:VAL 101	<**1.15**>	H- A:VAL 101
H- A:GLY 102	<**1.17**>	H- A:GLY 102
H- A:LEU 103	<**0.96**>	H- A:LEU 103
H- A:PHE 104	<**1.03**>	H- A:PHE 104
H+ A:GLU 105	<**1.11**>	H+ A:GLU 105
H+ A:ASP 106	<**0.99**>	H+ A:ASP 106
H. A:THR 107	<**0.84**>	H. A:THR 107
H+ A:ASN 108	<**0.95**>	H+ A:ASN 108
H- A:LEU 109	<**1.02**>	H- A:LEU 109
H- A:CYS 110	<**0.92**>	H- A:CYS 110
H- A:ALA 111	<**0.61**>	H- A:ALA 111
H- A:ILE 112	<**0.79**>	H- A:ILE 112
+ A:HIS 113	<**0.78**>	H+ A:HIS 113
- A:ALA 114	<**0.68**>	- A:ALA 114
+ A:LYS 115	<**0.78**>	+ A:LYS 115
+ A:ARG 116	<**0.90**>	+ A:ARG 116
- A:VAL 117	<**1.13**>	- A:VAL 117
. A:THR 118	<**1.24**>	S. A:THR 118
- A:ILE 119	<**1.15**>	S- A:ILE 119

- A:MET 120	<**0.96**>	- A:MET 120
+ A:PRO 121	<**0.65**>	+ A:PRO 121
H+ A:LYS 122	<**0.56**>	+ A:LYS 122
H+ A:ASP 123	<**0.78**>	+ A:ASP 123
H- A:ILE 124	<**0.90**>	H- A:ILE 124
H+ A:GLN 125	<**0.79**>	H+ A:GLN 125
H- A:LEU 126	<**0.78**>	H- A:LEU 126
H- A:ALA 127	<**0.66**>	H- A:ALA 127
H+ A:ARG 128	<**0.98**>	H+ A:ARG 128
H+ A:ARG 129	<**0.86**>	H+ A:ARG 129
H- A:ILE 130	<**0.94**>	H- A:ILE 130
H+ A:ARG 131	<**1.22**>	H+ A:ARG 131
- A:GLY 132	<**0.99**>	- A:GLY 132
+ A:GLU 133	<**0.73**>	+ A:GLU 133
+ A:ARG 134	<**0.79**>	+ A:ARG 134
- A:ALA 135		- A:ALA 135
+ B:LYS 20		+ B:LYS 20
- B:VAL 21	<**1.70**>	- B:VAL 21
- B:LEU 22	<**1.51**>	- B:LEU 22
+ B:ARG 23	<**1.27**>	+ B:ARG 23
+ B:ASP 24	<**1.44**>	+ B:ASP 24
+ B:ASN 25	<**1.38**>	+ B:ASN 25
- B:ILE 26	<**1.39**>	- B:ILE 26
+ B:GLN 27	<**1.28**>	+ B:GLN 27
- B:GLY 28	<**1.31**>	- B:GLY 28
- B:ILE 29	<**1.34**>	- B:ILE 29
. B:THR 30	<**1.19**>	. B:THR 30
H+ B:LYS 31	<**1.04**>	H+ B:LYS 31
H+ B:PRO 32	<**1.29**>	H+ B:PRO 32
H- B:ALA 33	<**1.22**>	H- B:ALA 33
H- B:ILE 34	<**1.14**>	H- B:ILE 34
H+ B:ARG 35	<**1.37**>	H+ B:ARG 35
H+ B:ARG 36	<**1.28**>	H+ B:ARG 36
H- B:LEU 37	<**1.24**>	H- B:LEU 37
H- B:ALA 38	<**1.23**>	H- B:ALA 38
H+ B:ARG 39	<**1.40**>	H+ B:ARG 39
H+ B:ARG 40	<**1.45**>	+ B:ARG 40
H- B:GLY 41	<**1.37**>	- B:GLY 41
- B:GLY 42	<**1.25**>	- B:GLY 42
- B:VAL 43	<**1.15**>	- B:VAL 43
+ B:LYS 44	<**1.30**>	+ B:LYS 44
+ B:ARG 45	<**1.22**>	S+ B:ARG 45
- B:ILE 46	<**1.32**>	S- B:ILE 46
. B:SER 47	<**1.25**>	. B:SER 47
- B:GLY 48	<**1.25**>	- B:GLY 48
- B:LEU 49	<**1.24**>	- B:LEU 49

- B:ILE 50	<**1.23**>	- B:ILE 50
H. B:TYR 51	<**1.13**>	H. B:TYR 51
H+ B:GLU 52	<**1.45**>	H+ B:GLU 52
H+ B:GLU 53	<**1.42**>	H+ B:GLU 53
H. B:THR 54	<**1.21**>	H. B:THR 54
H+ B:ARG 55	<**1.09**>	H+ B:ARG 55
H- B:GLY 56	<**1.13**>	H- B:GLY 56
H- B:VAL 57	<**1.14**>	H- B:VAL 57
H- B:LEU 58	<**1.12**>	H- B:LEU 58
H+ B:LYS 59	<**1.12**>	H+ B:LYS 59
H- B:VAL 60	<**1.12**>	H- B:VAL 60
H- B:PHE 61	<**1.09**>	H- B:PHE 61
H- B:LEU 62	<**1.03**>	H- B:LEU 62
H+ B:GLU 63	<**0.95**>	H+ B:GLU 63
H+ B:ASN 64	<**0.92**>	H+ B:ASN 64
H- B:VAL 65	<**1.02**>	H- B:VAL 65
H- B:ILE 66	<**0.92**>	H- B:ILE 66
H+ B:ARG 67	<**1.03**>	H+ B:ARG 67
H+ B:ASP 68	<**1.04**>	H+ B:ASP 68
H- B:ALA 69	<**0.95**>	H- B:ALA 69
H- B:VAL 70	<**1.02**>	H- B:VAL 70
H. B:THR 71	<**1.03**>	H. B:THR 71
H. B:TYR 72	<**0.97**>	H. B:TYR 72
H. B:THR 73	<**1.03**>	H. B:THR 73
H+ B:GLU 74	<**0.77**>	H+ B:GLU 74
H+ B:HIS 75	<**0.67**>	H+ B:HIS 75
- B:ALA 76	<**1.00**>	- B:ALA 76
+ B:LYS 77	<**1.07**>	+ B:LYS 77
+ B:ARG 78	<**1.37**>	+ B:ARG 78
+ B:LYS 79	<**1.19**>	+ B:LYS 79
S. B:THR 80	<**1.22**>	S. B:THR 80
S- B:VAL 81	<**1.19**>	S- B:VAL 81
. B:THR 82	<**1.12**>	. B:THR 82
- B:ALA 83	<**1.09**>	- B:ALA 83
H- B:MET 84	<**1.01**>	- B:MET 84
H+ B:ASP 85	<**0.91**>	H+ B:ASP 85
H- B:VAL 86	<**1.02**>	H- B:VAL 86
H- B:VAL 87	<**1.08**>	H- B:VAL 87
H. B:TYR 88	<**0.85**>	H. B:TYR 88
H- B:ALA 89	<**0.91**>	H- B:ALA 89
H- B:LEU 90	<**1.07**>	H- B:LEU 90
H+ B:LYS 91	<**1.12**>	H+ B:LYS 91
H+ B:ARG 92	<**1.00**>	H+ B:ARG 92
H+ B:GLN 93	<**1.26**>	+ B:GLN 93
- B:GLY 94	<**1.20**>	- B:GLY 94
+ B:ARG 95	<**1.12**>	+ B:ARG 95

S. B:THR 96	<**0.97**>	. B:THR 96
S- B:LEU 97	<**0.96**>	S- B:LEU 97
. B:TYR 98	<**1.14**>	. B:TYR 98
- B:GLY 99	<**1.13**>	- B:GLY 99
- B:PHE 100	<**1.04**>	- B:PHE 100
- B:GLY 101	<**1.32**>	- B:GLY 101
- B:GLY 102	<**1.31**>	- B:GLY 102
		+ C:LYS 9
		- C:ALA 10
		+ C:ARG 11
		- C:ALA 12
		+ C:LYS 13
		- C:ALA 14
		+ C:LYS 15
. C:THR 16	<**1.24**>	. C:THR 16
H+ C:ARG 17	<**0.92**>	H+ C:ARG 17
H. C:SER 18	<**1.06**>	H. C:SER 18
H. C:SER 19	<**1.05**>	H. C:SER 19
H+ C:ARG 20	<**1.00**>	H+ C:ARG 20
H- C:ALA 21	<**0.91**>	H- C:ALA 21
- C:GLY 22	<**1.13**>	- C:GLY 22
- C:LEU 23	<**1.13**>	- C:LEU 23
+ C:GLN 24	<**0.96**>	+ C:GLN 24
- C:PHE 25	<**1.09**>	- C:PHE 25
+ C:PRO 26	<**1.18**>	+ C:PRO 26
H- C:VAL 27	<**1.04**>	- C:VAL 27
H- C:GLY 28	<**0.75**>	H- C:GLY 28
H+ C:ARG 29	<**0.83**>	H+ C:ARG 29
H- C:VAL 30	<**0.83**>	H- C:VAL 30
H+ C:HIS 31	<**0.83**>	H+ C:HIS 31
H+ C:ARG 32	<**1.05**>	H+ C:ARG 32
H- C:LEU 33	<**1.08**>	H- C:LEU 33
H- C:LEU 34	<**1.06**>	H- C:LEU 34
H+ C:ARG 35	<**1.15**>	H+ C:ARG 35
H+ C:LYS 36	<**0.91**>	+ C:LYS 36
- C:GLY 37	<**0.74**>	- C:GLY 37
+ C:ASN 38	<**0.91**>	+ C:ASN 38
. C:TYR 39	<**0.87**>	. C:TYR 39
- C:ALA 40	<**0.79**>	- C:ALA 40
+ C:GLU 41	<**1.12**>	+ C:GLU 41
+ C:ARG 42	<**1.00**>	S+ C:ARG 42
S- C:VAL 43	<**0.81**>	S- C:VAL 43
- C:GLY 44	<**1.01**>	- C:GLY 44
- C:ALA 45	<**0.64**>	- C:ALA 45
- C:GLY 46	<**0.83**>	- C:GLY 46
- C:ALA 47	<**0.78**>	H- C:ALA 47

H+ C:PRO 48	<**0.74**>	H+ C:PRO 48
H- C:VAL 49	<**0.86**>	H- C:VAL 49
H. C:TYR 50	<**0.70**>	H. C:TYR 50
H- C:LEU 51	<**0.59**>	H- C:LEU 51
H- C:ALA 52	<**0.66**>	H- C:ALA 52
H- C:ALA 53	<**0.74**>	H- C:ALA 53
H- C:VAL 54	<**0.74**>	H- C:VAL 54
H- C:LEU 55	<**0.79**>	H- C:LEU 55
H+ C:GLU 56	<**0.79**>	H+ C:GLU 56
H. C:TYR 57	<**0.73**>	H. C:TYR 57
H- C:LEU 58	<**0.69**>	H- C:LEU 58
H. C:THR 59	<**0.76**>	H. C:THR 59
H- C:ALA 60	<**0.90**>	H- C:ALA 60
H+ C:GLU 61	<**0.96**>	H+ C:GLU 61
H- C:ILE 62	<**0.89**>	H- C:ILE 62
H- C:LEU 63	<**0.82**>	H- C:LEU 63
H+ C:GLU 64	<**0.74**>	H+ C:GLU 64
H- C:LEU 65	<**0.72**>	H- C:LEU 65
H- C:ALA 66	<**0.71**>	H- C:ALA 66
H- C:GLY 67	<**0.71**>	H- C:GLY 67
H+ C:ASN 68	<**0.73**>	H+ C:ASN 68
H- C:ALA 69	<**0.75**>	H- C:ALA 69
H- C:ALA 70	<**0.69**>	H- C:ALA 70
H+ C:ARG 71	<**0.61**>	H+ C:ARG 71
+ C:ASP 72	<**0.55**>	H+ C:ASP 72
+ C:ASN 73	<**0.58**>	H+ C:ASN 73
+ C:LYS 74	<**0.97**>	+ C:LYS 74
+ C:LYS 75	<**1.13**>	+ C:LYS 75
. C:THR 76	<**0.91**>	. C:THR 76
S+ C:ARG 77	<**0.96**>	S+ C:ARG 77
S- C:ILE 78	<**0.93**>	S- C:ILE 78
- C:ILE 79	<**0.79**>	- C:ILE 79
+ C:PRO 80	<**0.81**>	+ C:PRO 80
H+ C:ARG 81	<**0.88**>	H+ C:ARG 81
H+ C:HIS 82	<**0.95**>	H+ C:HIS 82
H- C:LEU 83	<**0.94**>	H- C:LEU 83
H+ C:GLN 84	<**0.89**>	H+ C:GLN 84
H- C:LEU 85	<**0.94**>	H- C:LEU 85
H- C:ALA 86	<**0.80**>	H- C:ALA 86
H- C:ILE 87	<**0.80**>	- C:ILE 87
+ C:ARG 88	<**1.16**>	+ C:ARG 88
+ C:ASN 89	<**0.56**>	+ C:ASN 89
+ C:ASP 90	<**0.50**>	+ C:ASP 90
H+ C:GLU 91	<**0.47**>	H+ C:GLU 91
H+ C:GLU 92	<**0.80**>	H+ C:GLU 92
H- C:LEU 93	<**0.87**>	H- C:LEU 93

H+ C:ASN 94	<**0.83**>	H+ C:ASN 94
H+ C:LYS 95	<**0.82**>	H+ C:LYS 95
H- C:LEU 96	<**0.75**>	H- C:LEU 96
H- C:LEU 97	<**0.76**>	H- C:LEU 97
- C:GLY 98	<**1.08**>	- C:GLY 98
+ C:LYS 99	<**1.14**>	+ C:LYS 99
S- C:VAL 100	<**1.08**>	- C:VAL 100
S. C:THR 101	<**1.05**>	S. C:THR 101
- C:ILE 102	<**1.15**>	- C:ILE 102
- C:ALA 103	<**1.18**>	- C:ALA 103
+ C:GLN 104	<**1.05**>	+ C:GLN 104
- C:GLY 105	<**0.87**>	- C:GLY 105
- C:GLY 106	<**1.05**>	- C:GLY 106
- C:VAL 107	<**1.00**>	- C:VAL 107
- C:LEU 108	<**0.69**>	- C:LEU 108
+ C:PRO 109	<**0.65**>	+ C:PRO 109
+ C:ASN 110	<**0.37**>	+ C:ASN 110
- C:ILE 111	<**0.76**>	- C:ILE 111
+ C:GLN 112	<**0.90**>	+ C:GLN 112
- C:ALA 113	<**1.15**>	- C:ALA 113
- C:VAL 114	<**1.00**>	- C:VAL 114
- C:LEU 115	<**0.52**>	- C:LEU 115
- C:LEU 116	<**0.65**>	- C:LEU 116
+ C:PRO 117	<**0.95**>	+ C:PRO 117
+ C:LYS 118	<**1.21**>	+ C:LYS 118
. D:SER 29	<**1.20**>	. D:SER 29
+ D:ARG 30	<**1.41**>	+ D:ARG 30
+ D:LYS 31	<**1.13**>	+ D:LYS 31
+ D:GLU 32	<**1.04**>	+ D:GLU 32
. D:SER 33	<**1.09**>	. D:SER 33
. D:TYR 34	<**1.20**>	. D:TYR 34
H. D:SER 35	<**1.24**>	. D:SER 35
H- D:VAL 36	<**1.26**>	- D:VAL 36
H. D:TYR 37	<**1.21**>	H. D:TYR 37
H- D:VAL 38	<**0.97**>	H- D:VAL 38
H. D:TYR 39	<**0.96**>	H. D:TYR 39
H+ D:LYS 40	<**1.18**>	H+ D:LYS 40
H- D:VAL 41	<**1.07**>	H- D:VAL 41
H- D:LEU 42	<**1.12**>	H- D:LEU 42
H+ D:LYS 43	<**1.11**>	H+ D:LYS 43
H+ D:GLN 44	<**1.19**>	H+ D:GLN 44
H- D:VAL 45	<**1.08**>	H- D:VAL 45
+ D:HIS 46	<**1.20**>	+ D:HIS 46
+ D:PRO 47	<**1.38**>	+ D:PRO 47
+ D:ASP 48	<**1.02**>	+ D:ASP 48
. D:THR 49	<**0.94**>	. D:THR 49

S- D:GLY 50	<**1.15**>	S- D:GLY 50
S- D:ILE 51	<**1.10**>	S- D:ILE 51
. D:SER 52	<**1.05**>	. D:SER 52
H. D:SER 53	<**0.87**>	H. D:SER 53
H+ D:LYS 54	<**0.96**>	H+ D:LYS 54
H- D:ALA 55	<**0.97**>	H- D:ALA 55
H- D:MET 56	<**0.97**>	H- D:MET 56
H- D:GLY 57	<**1.06**>	H- D:GLY 57
H- D:ILE 58	<**1.03**>	H- D:ILE 58
H- D:MET 59	<**0.81**>	H- D:MET 59
H+ D:ASN 60	<**0.67**>	H+ D:ASN 60
H. D:SER 61	<**0.76**>	H. D:SER 61
H- D:PHE 62	<**0.77**>	H- D:PHE 62
H- D:VAL 63	<**0.85**>	H- D:VAL 63
H+ D:ASN 64	<**0.55**>	H+ D:ASN 64
H+ D:ASP 65	<**0.79**>	H+ D:ASP 65
H- D:ILE 66	<**0.72**>	H- D:ILE 66
H- D:PHE 67	<**0.77**>	H- D:PHE 67
H+ D:GLU 68	<**0.99**>	H+ D:GLU 68
H+ D:ARG 69	<**0.85**>	H+ D:ARG 69
H- D:ILE 70	<**0.69**>	H- D:ILE 70
H- D:ALA 71	<**0.75**>	H- D:ALA 71
H- D:GLY 72	<**0.46**>	H- D:GLY 72
H+ D:GLU 73	<**0.63**>	H+ D:GLU 73
H- D:ALA 74	<**0.61**>	H- D:ALA 74
H. D:SER 75	<**0.59**>	H. D:SER 75
H+ D:ARG 76	<**0.67**>	H+ D:ARG 76
H- D:LEU 77	<**0.78**>	H- D:LEU 77
H- D:ALA 78	<**1.05**>	H- D:ALA 78
H+ D:HIS 79	<**0.99**>	H+ D:HIS 79
. D:TYR 80	<**0.64**>	H. D:TYR 80
+ D:ASN 81	<**0.60**>	H+ D:ASN 81
+ D:LYS 82	<**0.60**>	+ D:LYS 82
+ D:ARG 83	<**0.96**>	+ D:ARG 83
. D:SER 84	<**0.99**>	. D:SER 84
. D:THR 85	<**0.57**>	S. D:THR 85
S- D:ILE 86	<**0.69**>	S- D:ILE 86
. D:THR 87	<**0.80**>	. D:THR 87
. D:SER 88	<**0.86**>	. D:SER 88
H+ D:ARG 89	<**0.72**>	+ D:ARG 89
H+ D:GLU 90	<**0.65**>	H+ D:GLU 90
H- D:ILE 91	<**0.71**>	H- D:ILE 91
H+ D:GLN 92	<**0.79**>	H+ D:GLN 92
H. D:THR 93	<**0.85**>	H. D:THR 93
H- D:ALA 94	<**0.89**>	H- D:ALA 94
H- D:VAL 95	<**0.80**>	H- D:VAL 95

H+ D:ARG 96	<**0.89**>	H+ D:ARG 96
H- D:LEU 97	<**0.96**>	H- D:LEU 97
H- D:LEU 98	<**0.83**>	H- D:LEU 98
- D:LEU 99	<**0.54**>	- D:LEU 99
+ D:PRO 100	<**1.03**>	+ D:PRO 100
H- D:GLY 101	<**1.26**>	- D:GLY 101
H+ D:GLU 102	<**1.13**>	+ D:GLU 102
H- D:LEU 103	<**0.91**>	H- D:LEU 103
H- D:ALA 104	<**0.98**>	H- D:ALA 104
H+ D:LYS 105	<**0.91**>	H+ D:LYS 105
H+ D:HIS 106	<**0.90**>	H+ D:HIS 106
H- D:ALA 107	<**0.92**>	H- D:ALA 107
H- D:VAL 108	<**1.02**>	H- D:VAL 108
H. D:SER 109	<**0.96**>	H. D:SER 109
H+ D:GLU 110	<**1.06**>	H+ D:GLU 110
H- D:GLY 111	<**0.82**>	H- D:GLY 111
H. D:THR 112	<**0.87**>	H. D:THR 112
H+ D:LYS 113	<**0.87**>	H+ D:LYS 113
H- D:ALA 114	<**0.88**>	H- D:ALA 114
H- D:VAL 115	<**0.87**>	H- D:VAL 115
H. D:THR 116	<**0.81**>	H. D:THR 116
H+ D:LYS 117	<**0.69**>	H+ D:LYS 117
H. D:TYR 118	<**0.85**>	H. D:TYR 118
H. D:THR 119	<**0.97**>	H. D:THR 119
. D:SER 120	<**0.95**>	. D:SER 120
. D:SER 121	<**1.09**>	. D:SER 121
		+ D:LYS 122
+ E:HIS 39	<**1.90**>	+ E:HIS 39
+ E:ARG 40	<**1.43**>	+ E:ARG 40
. E:TYR 41	<**1.18**>	. E:TYR 41
+ E:ARG 42	<**1.14**>	+ E:ARG 42
+ E:PRO 43	<**1.16**>	+ E:PRO 43
- E:GLY 44	<**1.01**>	- E:GLY 44
H. E:THR 45	<**0.64**>	H. E:THR 45
H- E:VAL 46	<**0.98**>	H- E:VAL 46
H- E:ALA 47	<**1.00**>	H- E:ALA 47
H- E:LEU 48	<**0.97**>	H- E:LEU 48
H+ E:ARG 49	<**1.01**>	H+ E:ARG 49
H+ E:GLU 50	<**1.20**>	H+ E:GLU 50
H- E:ILE 51	<**1.17**>	H- E:ILE 51
H+ E:ARG 52	<**1.05**>	H+ E:ARG 52
H+ E:ARG 53	<**1.11**>	H+ E:ARG 53
H. E:TYR 54	<**1.06**>	H. E:TYR 54
H+ E:GLN 55	<**1.05**>	+ E:GLN 55
H+ E:LYS 56	<**1.20**>	+ E:LYS 56
. E:SER 57	<**0.97**>	. E:SER 57

. E:THR 58	<**0.88**>	. E:THR 58
+ E:GLU 59	<**0.84**>	+ E:GLU 59
- E:LEU 60	<**0.74**>	- E:LEU 60
- E:LEU 61	<**1.11**>	- E:LEU 61
- E:ILE 62	<**1.18**>	- E:ILE 62
+ E:ARG 63	<**1.31**>	+ E:ARG 63
+ E:LYS 64	<**1.14**>	+ E:LYS 64
H- E:LEU 65	<**1.11**>	H- E:LEU 65
H+ E:PRO 66	<**1.28**>	H+ E:PRO 66
H- E:PHE 67	<**1.11**>	H- E:PHE 67
H+ E:GLN 68	<**1.08**>	H+ E:GLN 68
H+ E:ARG 69	<**1.24**>	H+ E:ARG 69
H- E:LEU 70	<**1.27**>	H- E:LEU 70
H- E:VAL 71	<**1.19**>	H- E:VAL 71
H+ E:ARG 72	<**1.15**>	H+ E:ARG 72
H+ E:GLU 73	<**1.42**>	H+ E:GLU 73
H- E:ILE 74	<**1.34**>	H- E:ILE 74
H- E:ALA 75	<**1.17**>	- E:ALA 75
+ E:GLN 76	<**1.21**>	+ E:GLN 76
+ E:ASP 77	<**1.29**>	+ E:ASP 77
- E:PHE 78	<**1.18**>	- E:PHE 78
+ E:LYS 79	<**0.96**>	+ E:LYS 79
. E:THR 80	<**0.99**>	. E:THR 80
+ E:ASP 81	<**1.11**>	+ E:ASP 81
- E:LEU 82	<**1.06**>	- E:LEU 82
S+ E:ARG 83	<**1.08**>	+ E:ARG 83
S- E:PHE 84	<**1.12**>	- E:PHE 84
+ E:GLN 85	<**1.12**>	+ E:GLN 85
. E:SER 86	<**1.11**>	H. E:SER 86
H- E:ALA 87	<**1.09**>	H- E:ALA 87
H- E:ALA 88	<**1.00**>	H- E:ALA 88
H- E:ILE 89	<**1.01**>	H- E:ILE 89
H- E:GLY 90	<**1.03**>	H- E:GLY 90
H- E:ALA 91	<**0.95**>	H- E:ALA 91
H- E:LEU 92	<**0.99**>	H- E:LEU 92
H+ E:GLN 93	<**0.85**>	H+ E:GLN 93
H+ E:GLU 94	<**0.86**>	H+ E:GLU 94
H- E:ALA 95	<**0.74**>	H- E:ALA 95
H. E:SER 96	<**0.89**>	H. E:SER 96
H+ E:GLU 97	<**0.91**>	H+ E:GLU 97
H- E:ALA 98	<**0.96**>	H- E:ALA 98
H. E:TYR 99	<**0.86**>	H. E:TYR 99
H- E:LEU 100	<**0.98**>	H- E:LEU 100
H- E:VAL 101	<**0.86**>	H- E:VAL 101
H- E:GLY 102	<**0.82**>	H- E:GLY 102
H- E:LEU 103	<**0.78**>	H- E:LEU 103

H- E:PHE 104	<**0.87**>	H- E:PHE 104
H+ E:GLU 105	<**0.84**>	H+ E:GLU 105
H+ E:ASP 106	<**0.71**>	H+ E:ASP 106
H. E:THR 107	<**0.64**>	H. E:THR 107
H+ E:ASN 108	<**0.73**>	H+ E:ASN 108
H- E:LEU 109	<**0.74**>	H- E:LEU 109
H- E:CYS 110	<**0.82**>	H- E:CYS 110
H- E:ALA 111	<**0.68**>	H- E:ALA 111
H- E:ILE 112	<**0.66**>	H- E:ILE 112
+ E:HIS 113	<**0.51**>	H+ E:HIS 113
- E:ALA 114	<**0.49**>	- E:ALA 114
+ E:LYS 115	<**0.79**>	+ E:LYS 115
+ E:ARG 116	<**0.81**>	+ E:ARG 116
- E:VAL 117	<**0.88**>	- E:VAL 117
. E:THR 118	<**0.93**>	. E:THR 118
- E:ILE 119	<**0.85**>	- E:ILE 119
- E:MET 120	<**0.69**>	- E:MET 120
H+ E:PRO 121	<**0.63**>	+ E:PRO 121
H+ E:LYS 122	<**0.75**>	+ E:LYS 122
H+ E:ASP 123	<**0.66**>	+ E:ASP 123
H- E:ILE 124	<**0.79**>	H- E:ILE 124
H+ E:GLN 125	<**0.52**>	H+ E:GLN 125
H- E:LEU 126	<**0.68**>	H- E:LEU 126
H- E:ALA 127	<**0.66**>	H- E:ALA 127
H+ E:ARG 128	<**0.87**>	H+ E:ARG 128
H+ E:ARG 129	<**0.79**>	H+ E:ARG 129
H- E:ILE 130	<**0.72**>	H- E:ILE 130
H+ E:ARG 131	<**0.93**>	H+ E:ARG 131
- E:GLY 132	<**0.94**>	- E:GLY 132
+ E:GLU 133	<**0.84**>	+ E:GLU 133
+ E:ARG 134	<**1.05**>	+ E:ARG 134
- E:ALA 135	<**1.46**>	- E:ALA 135
		+ F:LYS 20
		- F:VAL 21
		- F:LEU 22
		+ F:ARG 23
		+ F:ASP 24
+ F:ASN 25	<**1.43**>	+ F:ASN 25
- F:ILE 26	<**1.40**>	- F:ILE 26
+ F:GLN 27	<**1.21**>	+ F:GLN 27
- F:GLY 28	<**1.14**>	- F:GLY 28
- F:ILE 29	<**1.06**>	- F:ILE 29
. F:THR 30	<**0.95**>	. F:THR 30
H+ F:LYS 31	<**0.96**>	H+ F:LYS 31
H+ F:PRO 32	<**1.19**>	H+ F:PRO 32
H- F:ALA 33	<**1.24**>	H- F:ALA 33

H- F:ILE 34	<**1.05**>	H- F:ILE 34
H+ F:ARG 35	<**0.92**>	H+ F:ARG 35
H+ F:ARG 36	<**0.87**>	H+ F:ARG 36
H- F:LEU 37	<**0.85**>	H- F:LEU 37
H- F:ALA 38	<**0.79**>	H- F:ALA 38
H+ F:ARG 39	<**0.89**>	H+ F:ARG 39
H+ F:ARG 40	<**1.01**>	+ F:ARG 40
H- F:GLY 41	<**0.97**>	- F:GLY 41
- F:GLY 42	<**0.79**>	- F:GLY 42
- F:VAL 43	<**0.66**>	- F:VAL 43
+ F:LYS 44	<**0.56**>	+ F:LYS 44
+ F:ARG 45	<**0.66**>	+ F:ARG 45
- F:ILE 46	<**0.89**>	- F:ILE 46
. F:SER 47	<**0.78**>	. F:SER 47
- F:GLY 48	<**0.67**>	- F:GLY 48
- F:LEU 49	<**0.93**>	- F:LEU 49
H- F:ILE 50	<**0.81**>	- F:ILE 50
H. F:TYR 51	<**0.91**>	H. F:TYR 51
H+ F:GLU 52	<**1.19**>	H+ F:GLU 52
H+ F:GLU 53	<**1.19**>	H+ F:GLU 53
H. F:THR 54	<**1.15**>	H. F:THR 54
H+ F:ARG 55	<**1.07**>	H+ F:ARG 55
H- F:GLY 56	<**1.00**>	H- F:GLY 56
H- F:VAL 57	<**1.02**>	H- F:VAL 57
H- F:LEU 58	<**0.91**>	H- F:LEU 58
H+ F:LYS 59	<**1.09**>	H+ F:LYS 59
H- F:VAL 60	<**1.17**>	H- F:VAL 60
H- F:PHE 61	<**1.03**>	H- F:PHE 61
H- F:LEU 62	<**0.98**>	H- F:LEU 62
H+ F:GLU 63	<**1.03**>	H+ F:GLU 63
H+ F:ASN 64	<**1.07**>	H+ F:ASN 64
H- F:VAL 65	<**1.00**>	H- F:VAL 65
H- F:ILE 66	<**1.03**>	H- F:ILE 66
H+ F:ARG 67	<**1.21**>	H+ F:ARG 67
H+ F:ASP 68	<**1.20**>	H+ F:ASP 68
H- F:ALA 69	<**1.16**>	H- F:ALA 69
H- F:VAL 70	<**1.19**>	H- F:VAL 70
H. F:THR 71	<**1.22**>	H. F:THR 71
H. F:TYR 72	<**1.20**>	H. F:TYR 72
H. F:THR 73	<**1.21**>	H. F:THR 73
H+ F:GLU 74	<**1.19**>	H+ F:GLU 74
H+ F:HIS 75	<**1.15**>	H+ F:HIS 75
H- F:ALA 76	<**1.27**>	- F:ALA 76
+ F:LYS 77	<**1.17**>	+ F:LYS 77
+ F:ARG 78	<**1.45**>	+ F:ARG 78
+ F:LYS 79	<**1.23**>	+ F:LYS 79

```
|S. F:THR  80 | <**1.27**> | . F:THR  80 |
|S- F:VAL  81 | <**1.32**> | - F:VAL  81 |
| . F:THR  82 | <**1.17**> | . F:THR  82 |
| - F:ALA  83 | <**0.98**> | - F:ALA  83 |
|H- F:MET  84 | <**0.98**> | - F:MET  84 |
|H+ F:ASP  85 | <**1.05**> |H+ F:ASP  85 |
|H- F:VAL  86 | <**1.13**> |H- F:VAL  86 |
|H- F:VAL  87 | <**1.17**> |H- F:VAL  87 |
|H. F:TYR  88 | <**0.96**> |H. F:TYR  88 |
|H- F:ALA  89 | <**1.03**> |H- F:ALA  89 |
|H- F:LEU  90 | <**1.10**> |H- F:LEU  90 |
|H+ F:LYS  91 | <**1.02**> |H+ F:LYS  91 |
|H+ F:ARG  92 | <**0.99**> |H+ F:ARG  92 |
|H+ F:GLN  93 | <**1.27**> | + F:GLN  93 |
| - F:GLY  94 | <**1.21**> | - F:GLY  94 |
| + F:ARG  95 | <**1.21**> | + F:ARG  95 |
|S. F:THR  96 | <**0.94**> | . F:THR  96 |
|S- F:LEU  97 | <**0.95**> |S- F:LEU  97 |
| . F:TYR  98 | <**0.98**> | . F:TYR  98 |
| - F:GLY  99 | <**1.01**> | - F:GLY  99 |
| - F:PHE 100 | <**0.95**> | - F:PHE 100 |
| - G:ALA  14 |            | - F:GLY 101 |
|             |            | - F:GLY 102 |
|             |            | + G:LYS   9 |
|             |            | - G:ALA  10 |
|             |            | + G:ARG  11 |
|             |            | - G:ALA  12 |
|             |            | + G:LYS  13 |
|             |            | - G:ALA  14 |
| + G:LYS  15 | <**0.86**> | + G:LYS  15 |
| . G:THR  16 | <**0.93**> | . G:THR  16 |
|H+ G:ARG  17 | <**0.96**> |H+ G:ARG  17 |
|H. G:SER  18 | <**1.21**> |H. G:SER  18 |
|H. G:SER  19 | <**1.20**> |H. G:SER  19 |
|H+ G:ARG  20 | <**1.10**> |H+ G:ARG  20 |
|H- G:ALA  21 | <**1.03**> |H- G:ALA  21 |
| - G:GLY  22 | <**1.13**> | - G:GLY  22 |
| - G:LEU  23 | <**1.12**> | - G:LEU  23 |
| + G:GLN  24 | <**1.05**> | + G:GLN  24 |
| - G:PHE  25 | <**1.13**> | - G:PHE  25 |
| + G:PRO  26 | <**0.99**> | + G:PRO  26 |
|H- G:VAL  27 | <**0.80**> | - G:VAL  27 |
|H- G:GLY  28 | <**0.74**> |H- G:GLY  28 |
|H+ G:ARG  29 | <**0.73**> |H+ G:ARG  29 |
|H- G:VAL  30 | <**0.67**> |H- G:VAL  30 |
|H+ G:HIS  31 | <**0.73**> |H+ G:HIS  31 |
```

H+ G:ARG 32	<**1.02**>	H+ G:ARG 32
H- G:LEU 33	<**0.92**>	H- G:LEU 33
H- G:LEU 34	<**0.85**>	H- G:LEU 34
H+ G:ARG 35	<**1.08**>	H+ G:ARG 35
H+ G:LYS 36	<**0.94**>	+ G:LYS 36
- G:GLY 37	<**0.84**>	- G:GLY 37
+ G:ASN 38	<**0.91**>	+ G:ASN 38
. G:TYR 39	<**0.72**>	. G:TYR 39
- G:ALA 40	<**0.79**>	- G:ALA 40
+ G:GLU 41	<**1.24**>	+ G:GLU 41
+ G:ARG 42	<**0.54**>	+ G:ARG 42
S- G:VAL 43	<**0.86**>	S- G:VAL 43
- G:GLY 44	<**0.99**>	- G:GLY 44
- G:ALA 45	<**1.24**>	- G:ALA 45
- G:GLY 46	<**0.89**>	- G:GLY 46
H- G:ALA 47	<**0.86**>	H- G:ALA 47
H+ G:PRO 48	<**0.87**>	H+ G:PRO 48
H- G:VAL 49	<**0.87**>	H- G:VAL 49
H. G:TYR 50	<**0.82**>	H. G:TYR 50
H- G:LEU 51	<**0.75**>	H- G:LEU 51
H- G:ALA 52	<**0.87**>	H- G:ALA 52
H- G:ALA 53	<**0.92**>	H- G:ALA 53
H- G:VAL 54	<**0.87**>	H- G:VAL 54
H- G:LEU 55	<**0.95**>	H- G:LEU 55
H+ G:GLU 56	<**0.89**>	H+ G:GLU 56
H. G:TYR 57	<**0.93**>	H. G:TYR 57
H- G:LEU 58	<**0.91**>	H- G:LEU 58
H. G:THR 59	<**0.87**>	H. G:THR 59
H- G:ALA 60	<**1.01**>	H- G:ALA 60
H+ G:GLU 61	<**1.15**>	H+ G:GLU 61
H- G:ILE 62	<**1.08**>	H- G:ILE 62
H- G:LEU 63	<**0.79**>	H- G:LEU 63
H+ G:GLU 64	<**0.94**>	H+ G:GLU 64
H- G:LEU 65	<**1.02**>	H- G:LEU 65
H- G:ALA 66	<**0.77**>	H- G:ALA 66
H- G:GLY 67	<**0.75**>	H- G:GLY 67
H+ G:ASN 68	<**0.86**>	H+ G:ASN 68
H- G:ALA 69	<**0.84**>	H- G:ALA 69
H- G:ALA 70	<**0.59**>	H- G:ALA 70
H+ G:ARG 71	<**0.61**>	H+ G:ARG 71
+ G:ASP 72	<**0.46**>	H+ G:ASP 72
+ G:ASN 73	<**0.79**>	H+ G:ASN 73
+ G:LYS 74	<**0.99**>	+ G:LYS 74
+ G:LYS 75	<**1.08**>	+ G:LYS 75
. G:THR 76	<**0.93**>	. G:THR 76
S+ G:ARG 77	<**0.97**>	+ G:ARG 77

S- G:ILE 78	<**0.91**>	- G:ILE 78
- G:ILE 79	<**0.78**>	- G:ILE 79
+ G:PRO 80	<**0.80**>	+ G:PRO 80
H+ G:ARG 81	<**0.81**>	H+ G:ARG 81
H+ G:HIS 82	<**0.93**>	H+ G:HIS 82
H- G:LEU 83	<**0.96**>	H- G:LEU 83
H+ G:GLN 84	<**0.87**>	H+ G:GLN 84
H- G:LEU 85	<**0.96**>	H- G:LEU 85
H- G:ALA 86	<**0.96**>	H- G:ALA 86
H- G:ILE 87	<**0.80**>	- G:ILE 87
H+ G:ARG 88	<**1.19**>	+ G:ARG 88
+ G:ASN 89	<**1.05**>	+ G:ASN 89
+ G:ASP 90	<**1.03**>	+ G:ASP 90
+ G:GLU 91	<**0.71**>	H+ G:GLU 91
H+ G:GLU 92	<**0.93**>	H+ G:GLU 92
H- G:LEU 93	<**0.96**>	H- G:LEU 93
H+ G:ASN 94	<**0.84**>	H+ G:ASN 94
H+ G:LYS 95	<**0.82**>	H+ G:LYS 95
H- G:LEU 96	<**0.89**>	H- G:LEU 96
H- G:LEU 97	<**0.81**>	H- G:LEU 97
- G:GLY 98	<**1.08**>	- G:GLY 98
+ G:LYS 99	<**1.13**>	+ G:LYS 99
S- G:VAL 100	<**1.16**>	- G:VAL 100
S. G:THR 101	<**1.11**>	S. G:THR 101
- G:ILE 102	<**1.09**>	- G:ILE 102
- G:ALA 103	<**1.12**>	- G:ALA 103
+ G:GLN 104	<**0.94**>	+ G:GLN 104
- G:GLY 105	<**0.77**>	- G:GLY 105
- G:GLY 106	<**1.08**>	- G:GLY 106
- G:VAL 107	<**1.02**>	- G:VAL 107
- G:LEU 108	<**0.71**>	- G:LEU 108
+ G:PRO 109	<**0.83**>	+ G:PRO 109
+ G:ASN 110	<**0.82**>	+ G:ASN 110
- G:ILE 111	<**0.87**>	- G:ILE 111
+ G:GLN 112	<**1.02**>	+ G:GLN 112
- G:ALA 113	<**0.92**>	- G:ALA 113
- G:VAL 114	<**0.91**>	- G:VAL 114
- G:LEU 115	<**0.74**>	- G:LEU 115
- G:LEU 116	<**0.74**>	- G:LEU 116
+ G:PRO 117	<**0.56**>	+ G:PRO 117
+ G:LYS 118	<**1.21**>	+ G:LYS 118
. H:SER 29	<**1.27**>	. H:SER 29
+ H:ARG 30	<**1.47**>	+ H:ARG 30
+ H:LYS 31	<**1.14**>	+ H:LYS 31
+ H:GLU 32	<**1.05**>	+ H:GLU 32
. H:SER 33	<**1.07**>	. H:SER 33

. H:TYR 34	<**1.15**>	. H:TYR 34
H. H:SER 35	<**1.23**>	. H:SER 35
H- H:VAL 36	<**1.26**>	- H:VAL 36
H. H:TYR 37	<**1.19**>	H. H:TYR 37
H- H:VAL 38	<**0.83**>	H- H:VAL 38
H. H:TYR 39	<**0.84**>	H. H:TYR 39
H+ H:LYS 40	<**1.15**>	H+ H:LYS 40
H- H:VAL 41	<**0.93**>	H- H:VAL 41
H- H:LEU 42	<**0.98**>	H- H:LEU 42
H+ H:LYS 43	<**0.99**>	H+ H:LYS 43
H+ H:GLN 44	<**0.97**>	H+ H:GLN 44
H- H:VAL 45	<**0.66**>	H- H:VAL 45
+ H:HIS 46	<**0.92**>	+ H:HIS 46
+ H:PRO 47	<**1.38**>	+ H:PRO 47
+ H:ASP 48	<**1.12**>	+ H:ASP 48
. H:THR 49	<**1.15**>	. H:THR 49
S- H:GLY 50	<**1.22**>	- H:GLY 50
S- H:ILE 51	<**0.97**>	- H:ILE 51
. H:SER 52	<**0.84**>	. H:SER 52
H. H:SER 53	<**0.68**>	H. H:SER 53
H+ H:LYS 54	<**0.84**>	H+ H:LYS 54
H- H:ALA 55	<**0.82**>	H- H:ALA 55
H- H:MET 56	<**0.76**>	H- H:MET 56
H- H:GLY 57	<**0.88**>	H- H:GLY 57
H- H:ILE 58	<**0.80**>	H- H:ILE 58
H- H:MET 59	<**0.52**>	H- H:MET 59
H+ H:ASN 60	<**0.56**>	H+ H:ASN 60
H. H:SER 61	<**0.55**>	H. H:SER 61
H- H:PHE 62	<**0.51**>	H- H:PHE 62
H- H:VAL 63	<**0.68**>	H- H:VAL 63
H+ H:ASN 64	<**0.49**>	H+ H:ASN 64
H+ H:ASP 65	<**0.66**>	H+ H:ASP 65
H- H:ILE 66	<**0.73**>	H- H:ILE 66
H- H:PHE 67	<**0.80**>	H- H:PHE 67
H+ H:GLU 68	<**0.95**>	H+ H:GLU 68
H+ H:ARG 69	<**0.88**>	H+ H:ARG 69
H- H:ILE 70	<**0.76**>	H- H:ILE 70
H- H:ALA 71	<**0.79**>	H- H:ALA 71
H- H:GLY 72	<**0.82**>	H- H:GLY 72
H+ H:GLU 73	<**0.82**>	H+ H:GLU 73
H- H:ALA 74	<**0.83**>	H- H:ALA 74
H. H:SER 75	<**0.90**>	H. H:SER 75
H+ H:ARG 76	<**0.88**>	H+ H:ARG 76
H- H:LEU 77	<**0.80**>	H- H:LEU 77
H- H:ALA 78	<**1.12**>	H- H:ALA 78
H+ H:HIS 79	<**1.16**>	H+ H:HIS 79

```
| . H:TYR  80 | <**1.02**> |H. H:TYR  80 |
| + H:ASN  81 | <**0.95**> |H+ H:ASN  81 |
| + H:LYS  82 | <**0.97**> | + H:LYS  82 |
| + H:ARG  83 | <**1.07**> | + H:ARG  83 |
| . H:SER  84 | <**1.14**> | . H:SER  84 |
| . H:THR  85 | <**0.92**> | . H:THR  85 |
|S- H:ILE  86 | <**0.94**> |S- H:ILE  86 |
| . H:THR  87 | <**0.93**> | . H:THR  87 |
| . H:SER  88 | <**0.93**> | . H:SER  88 |
|H+ H:ARG  89 | <**0.78**> | + H:ARG  89 |
|H+ H:GLU  90 | <**0.66**> |H+ H:GLU  90 |
|H- H:ILE  91 | <**0.79**> |H- H:ILE  91 |
|H+ H:GLN  92 | <**0.89**> |H+ H:GLN  92 |
|H. H:THR  93 | <**0.94**> |H. H:THR  93 |
|H- H:ALA  94 | <**0.94**> |H- H:ALA  94 |
|H- H:VAL  95 | <**0.84**> |H- H:VAL  95 |
|H+ H:ARG  96 | <**0.93**> |H+ H:ARG  96 |
|H- H:LEU  97 | <**1.06**> |H- H:LEU  97 |
|H- H:LEU  98 | <**0.99**> |H- H:LEU  98 |
| - H:LEU  99 | <**0.72**> | - H:LEU  99 |
| + H:PRO 100 | <**0.86**> | + H:PRO 100 |
|H- H:GLY 101 | <**1.26**> | - H:GLY 101 |
|H+ H:GLU 102 | <**1.06**> | + H:GLU 102 |
|H- H:LEU 103 | <**0.71**> |H- H:LEU 103 |
|H- H:ALA 104 | <**0.83**> |H- H:ALA 104 |
|H+ H:LYS 105 | <**0.90**> |H+ H:LYS 105 |
|H+ H:HIS 106 | <**0.83**> |H+ H:HIS 106 |
|H- H:ALA 107 | <**0.97**> |H- H:ALA 107 |
|H- H:VAL 108 | <**1.05**> |H- H:VAL 108 |
|H. H:SER 109 | <**0.94**> |H. H:SER 109 |
|H+ H:GLU 110 | <**1.04**> |H+ H:GLU 110 |
|H- H:GLY 111 | <**0.91**> |H- H:GLY 111 |
|H. H:THR 112 | <**1.00**> |H. H:THR 112 |
|H+ H:LYS 113 | <**0.98**> |H+ H:LYS 113 |
|H- H:ALA 114 | <**1.00**> |H- H:ALA 114 |
|H- H:VAL 115 | <**1.00**> |H- H:VAL 115 |
|H. H:THR 116 | <**0.96**> |H. H:THR 116 |
|H+ H:LYS 117 | <**0.84**> |H+ H:LYS 117 |
|H. H:TYR 118 | <**0.97**> |H. H:TYR 118 |
|H. H:THR 119 | <**1.15**> |H. H:THR 119 |
| . H:SER 120 | <**1.16**> | . H:SER 120 |
| . H:SER 121 | <**1.27**> | . H:SER 121 |
|             |            | + H:LYS 122 |
`-------------'------------'-------------'
```

NCP traces					NCP + Int. traces					NCP + Mg traces				
0	296	3673.3333	0.92542828	NCP	0	142	4474.3333	0.96923966	NCP + Int.	0	27	5224	0.99485812	NCP + Mg
0.10003287	168.66667	3370	0.9523361	FRET histogram	0.10003318	171.33333	4643.6667	0.96441675	FRET histogram	0.10003324	-85.333333	3867.6667	1.022561	FRET histogram
0.20006575	121	3375.3333	0.96539232	0.6	0.20006636	154.33333	4589	0.96746311	0.6	0.20006649	-156	3304.3333	1.04955	0.6
0.30009862	239.33333	3276.3333	0.93192377	0.6	0.30009954	213.33333	4215	0.95182537	0.6	0.30009973	-84.333333	3188	1.0271722	0.6
0.40013149	417.33333	3391.3333	0.89042535	0.6	0.40013271	236.33333	4554	0.95066453	0.6	0.40013298	-2.3333333	3410	1.0006847	0.62
0.50016437	420.66667	3223.6667	0.88456965	0.6	0.50016589	177.66667	4770	0.96409082	0.62	0.50016622	-45.666667	4361	1.0105824	0.65
0.60019724	245.33333	3260	0.93001141	0.65	0.60019907	-25.666667	4894.3333	1.0052718	0.64	0.60019947	22.666667	4555	0.99504842	0.7
0.70023011	241	3052.6667	0.92682927	0.68	0.70023225	-21	4480.6667	1.0047089	0.65	0.70023271	66.666667	4730.3333	0.98610243	0.72
0.80026298	288.66667	3108.6667	0.9150314	0.7	0.80026543	40.666667	4391.6667	0.990825	0.65	0.80026596	206.33333	3650	0.94649494	0.75
0.90029586	577	3063.6667	0.84151254	0.72	0.90029861	174	4780.3333	0.96487923	0.66	0.9002992	98.333333	3459	0.97235757	0.8
1.0003287	467.66667	3323.3333	0.87663765	0.73	1.0003318	151.33333	5008.6667	0.97067183	0.68	1.0003324	16	3286	0.99515445	0.8
1.1003616	463	3247.6667	0.87522458	0.74	1.100365	43.333333	5232	0.99178567	0.68	1.1003657	-69.666667	3551.3333	1.0200096	0.8
1.2003945	408	3551	0.89694367	0.75	1.2003981	369.33333	4876.3333	0.92959268	0.7	1.2003989	-134	4040	1.0343062	0.85
1.3004274	380.66667	3487	0.90157718	0.75	1.3004313	675.66667	4209.3333	0.86168543	0.7	1.3004322	-191.66667	4032.3333	1.0499045	0.85
1.4004602	388	3848.3333	0.90841136	0.75	1.4004645	719.66667	4551.6667	0.8634754	0.72	1.4004654	-156.66667	4001.3333	1.0407491	0.85
1.5004931	74	3546.3333	0.97955989	0.76	1.5004977	407.66667	4450	0.91607768	0.72	1.5004987	-69.333333	3527	1.0200521	0.87
1.600526	143.33333	3405.6667	0.95961304	0.76	1.6005309	51.666667	5402.3333	0.99052683	0.75	1.6005319	85	3746.6667	0.97781644	0.9
1.7005588	117	3140	0.96407737	0.76	1.700564	155.33333	4979	0.96974615	0.75	1.7005652	295.33333	3923.3333	0.92999368	0.9
1.8005917	217.66667	3662.3333	0.94390034	0.77	1.8005972	116.66667	5549.3333	0.97940934	0.75	1.8005984	398.33333	4132.3333	0.91208064	0.9
1.9006246	274	3998.3333	0.93586643	0.78	1.9006304	93	5281	0.98269445	0.78	1.9006316	319.33333	3883.3333	0.9240165	0.9
2.0006575	404.33333	3780.3333	0.90337741	0.79	2.0006636	466.66667	5355.6667	0.91984886	0.78	2.0006649	20	3244.3333	0.99387317	0.9
2.1006903	529.66667	3503	0.86865598	0.8	2.1006967	306.66667	4900	0.94110115	0.8	2.1006981	92.666667	3109.3333	0.97105975	0.9
2.2007232	419	3145.3333	0.88244646	0.8	2.2007299	331	4650.3333	0.93355193	0.8	2.2007314	63.333333	3477.6667	0.98211428	0.9
2.3007561	226.66667	3641.6667	0.94140457	0.82	2.3007631	226	3951.3333	0.9458985	0.8	2.3007646	118	4127	0.97220259	0.91
2.400789	121	3430.3333	0.96592829	0.85	2.4007963	566.66667	3678.3333	0.86650962	0.83	2.4007979	17.666667	4415.6667	0.99601504	0.92
2.5008218	267	3491	0.92895157	0.85	2.5008295	532.33333	3593.3333	0.87097035	0.84	2.5008311	107.33333	4268	0.97546854	0.92
2.6008547	350.33333	3133.6667	0.89944508	0.87	2.6008626	182.66667	4330.3333	0.95952434	0.84	2.6008644	269.33333	3848	0.93458549	0.92
2.7008876	394	3221	0.89100968	0.87	2.7008958	86.333333	4596.6667	0.98156452	0.85	2.7008976	202	3405.3333	0.94400296	0.93
2.8009204	210.33333	2992.3333	0.93432556	0.87	2.800929	187	4576.6667	0.96074452	0.85	2.8009309	146.66667	3631.3333	0.96117875	0.93
2.9009533	120	3080.6667	0.96250781	0.87	2.9009622	333.66667	4693	0.93362069	0.86	2.9009641	85.333333	3984.3333	0.97903186	0.93
3.0009862	159.66667	3353	0.95454545	0.88	3.0009954	235	4586.3333	0.9512583	0.86	3.0009973	22	4219	0.99481254	0.95
3.1010191	140	3776.3333	0.96425228	0.88	3.1010285	51	4929	0.98975904	0.86	3.1010306	-102	3963.6667	1.0264135	0.95
3.2010519	14.666667	4095.3333	0.99643147	0.89	3.2010617	67	4404	0.98501454	0.87	3.2010638	-173	3724.6667	1.0487095	0.95
3.3010848	-170.66667	4199.3333	1.0423631	0.9	3.3010949	42.333333	4558.6667	0.9907991	0.87	3.3010971	-155	3499	1.0463517	0.97
3.4011177	50.333333	4179.3333	0.98809993	0.9	3.4011281	111.66667	4109	0.97354288	0.88	3.4011303	-4.3333333	3416	1.0012702	0.98
3.5011506	111	3816	0.97173415	0.9	3.5011612	-67.333333	4578.6667	1.0149254	0.89	3.5011636	17.333333	3679.3333	0.99531109	0.98
3.6011834	134.33333	3546	0.96349968	0.9	3.6011944	11.666667	4631.3333	0.99748726	0.9	3.6011968	84	4155.3333	0.98018556	0.98
3.7012163	-64	3406	1.0191502	0.9	3.7012276	27	4713	0.9943038	0.9	3.7012301	28.333333	4373.3333	0.99356304	0.98
3.8012492	34	3801	0.99113429	0.9	3.8012608	136.33333	4281.6667	0.96914139	0.9	3.8012633	77	4391	0.98276634	0.98
3.9012821	-7.6666667	3814	1.0020142	0.9	3.901294	218.66667	4313.3333	0.95175051	0.9	3.9012965	197	4345	0.95662704	0.98
4.0013149	219.66667	4326.6667	0.95168267	0.91	4.0013271	297	3944.3333	0.92997485	0.9	4.0013298	71	4762	0.98530933	0.98
4.1013478	260.33333	3716.3333	0.93453479	0.91	4.1013603	484.33333	4029	0.89268833	0.9	4.101363	-25	4709	1.0053373	0.98
4.2013807	450	3830.6667	0.89487619	0.92	4.2013935	467	4139.6667	0.89862518	0.9	4.2013963	-95.666667	4726.3333	1.0206594	1
4.3014135	487.66667	3610.3333	0.88099886	0.92	4.3014267	439.66667	4340.3333	0.90801953	0.9	4.3014295	41.333333	4059.6667	0.98992116	1
4.4014464	460	4147.6667	0.90016639	0.92	4.4014599	44	4015	0.98915989	0.9	4.4014628	53.666667	3902.6667	0.98643525	
4.5014793	489	3802.6667	0.88605825	0.93	4.501493	29.666667	3795.3333	0.99224401	0.9	4.501496	178	3804	0.95529884	
4.6015122	326	3277	0.90951984	0.93	4.6015262	-63	5018.3333	1.0127136	0.91	4.6015293	29.333333	3756.6667	0.99225216	
4.701545	274	2920	0.91421415	0.94	4.7015594	318.33333	5344.3333	0.94378385	0.91	4.7015625	155	3907.3333	0.96184459	
4.8015779	318.33333	2897	0.90099523	0.94	4.8015926	335.33333	5172	0.93911149	0.91	4.8015957	157	3949	0.96176327	
4.9016108	270.66667	3494.6667	0.92811615	0.94	4.9016257	565.33333	4390.6667	0.88592951	0.91	4.901629	348.66667	4198	0.92331378	
5.0016437	400.33333	3427	0.8954015	0.94	5.0016589	352.33333	3972.6667	0.91853565	0.92	5.0016622	321.66667	3902.6667	0.92385386	
5.1016765	226	3897.6667	0.94519441	0.94	5.1016921	246	4551.3333	0.94872151	0.92	5.1016955	1	3816.3333	0.99973804	
5.2017094	315	3802	0.92348798	0.94	5.2017253	176	4347.6667	0.96109351	0.92	5.2017287	29.333333	3375.6667	0.99138522	
5.3017423	169.66667	4128	0.96052121	0.94	5.3017585	204	5001.6667	0.96081194	0.92	5.301762	-9	3528.3333	1.0025573	
5.4017751	195	3872.3333	0.95205704	0.94	5.4017916	468.66667	4749.6667	0.91018844	0.92	5.4017952	177.33333	3473.3333	0.9514244	
5.501808	179	3611.6667	0.95277875	0.95	5.5018248	423	4883.3333	0.92028394	0.92	5.5018285	42.333333	3507.6667	0.98807512	
5.6018409	277.33333	3678.3333	0.92988961	0.95	5.601858	425.33333	4814	0.91881919	0.92	5.6018617	-146	3543	1.0429791	
5.7018738	440.33333	3438.6667	0.88648277	0.95	5.7018912	4.6666667	4668	0.99900128	0.92	5.7018949	26	3502.3333	0.99263108	
5.8019066	357	3477.6667	0.90690195	0.95	5.8019244	239.66667	4609	0.9505706	0.92	5.8019282	-5.6666667	3469	1.0016362	
5.9019395	352.66667	2886	0.89110745	0.95	5.9019575	90	4653	0.98102467	0.92	5.9019614	151	3559.6667	0.9593065	
6.0019724	280	3176	0.91898148	0.95	6.0019907	318.33333	5085	0.94108575	0.92	6.0019947	-79.666667	3905.6667	1.0208224	
6.1020053	233	3121	0.93053071	0.95	6.1020239	220	4936	0.95733126	0.92	6.1020279	-54.666667	3979	1.0139302	
6.2020381	216.66667	3436.6667	0.94069343	0.95	6.2020571	490	5274.3333	0.91499451	0.94	6.2020612	-31.666667	3747	1.0085232	
6.302071	4	3002.3333	0.99866948	0.95	6.3020902	360.66667	4825	0.93044932	0.94	6.3020944	-95	3476.6667	1.0280927	
6.4021039	155.33333	3297.3333	0.95501062	0.95	6.4021234	581.33333	4871.6667	0.89339202	0.94	6.4021277	-42.666667	3720.3333	1.0116016	
6.5021368	100.66667	3122.3333	0.96876616	0.95	6.5021566	502.33333	4434	0.89823756	0.94	6.5021609	-83.666667	3905.6667	1.0218908	
6.6021696	335.66667	3682.6667	0.9164662	0.95	6.6021898	542.33333	4093.3333	0.88300856	0.94	6.6021941	-48.333333	3856	1.0126937	
6.7022025	527.33333	3512	0.8694504	0.96	6.702223	273.33333	4274.3333	0.93989592	0.94	6.7022274	-74.666667	3066.6667	1.0249554	
6.8022354	564	3953	0.87513837	0.96	6.8022561	250.33333	4095.3333	0.94239472	0.94	6.8022606	-129.66667	2897.6667	1.0468449	
6.9022682	419.33333	3633.3333	0.89652903	0.96	6.9022893	639.33333	4590.3333	0.87774874	0.94	6.9022939	-162.66667	2519.6667	1.0690143	
7.0023011	426.66667	3570.3333	0.89325327	0.96	7.0023225	403	4454	0.91702697	0.95	7.0023271	-206.33333	3379	1.0650347	
7.102334	445.33333	3015.6667	0.87132813	0.96	7.1023557	462.33333	5135	0.91740114	0.95	7.1023604	-133.33333	3442.6667	1.0402901	
7.2023669	414.33333	3457.3333	0.89298321	0.96	7.2023889	272.66667	5198	0.95015842	0.95	7.2023936	-59.666667	3896	1.015553	
7.3023997	112.33333	3431.3333	0.96830025	0.97	7.302422	550.33333	5622	0.91083869	0.95	7.3024269	108.33333	4129.3333	0.97443562	
7.4024326	330.66667	4087	0.92514902	0.97	7.4024552	692.66667	4739.6667	0.87249187	0.95	7.4024601	12.333333	4196.6667	0.99706977	
7.5024655	313.33333	3819	0.9241752	0.97	7.5024884	693.66667	4527.6667	0.8671476	0.95	7.5024934	-36	4246.3333	1.0085504	
7.6024984	326	4152	0.92719964	0.97	7.6025216	596.33333	4505.3333	0.88311009	0.95	7.6025266	-187.66667	4029	1.0488546	
7.7025312	172.33333	3814.3333	0.95677258	0.98	7.7025547	131.33333	4886	0.97382408	0.95	7.7025598	0.33333333	3933	0.99991525	
7.8025641	177	3695	0.95428719	0.98	7.8025879	-143.33333	4968	1.0297084	0.95	7.8025931	-104.66667	3975.3333	1.027041	
7.902597	407	3566	0.89755852	0.98	7.9026211	-303.66667	5114.3333	1.0631236	0.95	7.9026263	-2	3709.6667	1.0005394	
8.0026298	329.33333	2980.6667	0.90050352	0.98	8.0026543	110	5251.3333	0.97948272	0.95	8.0026596	-108	3827.3333	1.0290375	
8.1026627	389.66667	3444	0.89835666	0.98	8.1026875	77.333333	5670.6667	0.98654605	0.96	8.1026928	16.666667	3555	0.99533364	
8.2026956	324	3182	0.90758699	0.98	8.2027206	180	5660.3333	0.96917984	0.96	8.2027261	-82.333333	3810.3333	1.0220851	
8.3027285	437.33333	3800	0.89679043	0.98	8.3027538	-116.33333	5677	1.0209208	0.96	8.3027593	9	3584	0.99749513	
8.4027613	308.66667	4137	0.93056909	0.98	8.402787	507	4955.6667	0.90718819	0.97	8.4027926	-72.333333	3405	1.0217043	
8.5027942	229.33333	4398.3333	0.95044299	0.98	8.5028202	488.33333	4380.6667	0.89970562	0.97	8.5028258	-77.666667	3531	1.0224903	
8.6028271	181	4556.6667	0.96179554	0.98	8.6028534	538.66667	4638	0.89594334	0.98	8.602859	-62.333333	3590.6667	1.0176665	
8.70286	387.33333	3410.3333	0.89800755	0.99	8.7028865	33	5191.6667	0.99368381	0.98	8.7028923	99.666667	3987.3333	0.97561373	
8.8028928	512.33333	3331.6667	0.8667187	0.99	8.8029197	55.333333	5903.3333	0.99071381	0.98	8.8029255	225	3606	0.9412686	
8.9029257	513	3202.3333	0.86192356	0.99	8.9029529	64.666667	5580.3333	0.98854443	0.99	8.9029588	26	3646.3333	0.99292003	
9.0029586	550.33333	3609.3333	0.86769773	1	9.0029861	349.66667	5338	0.93852195	0.99	9.002992	21	3317.3333	0.99370944	
9.1029915	689.66667	3578.3333	0.83840987	1	9.1030192	429.66667	4970.3333	0.9204321	0.99	9.1030253	-93.666667	3656.3333	1.0262912	
9.2030243	550.33333	3662.6667	0.86937258	1	9.2030524	518.66667	4792.3333	0.90234105	1	9.2030585	-10.666667	3716.6667	1.0028782	
9.3030572	442.33333	3804.6667	0.89584805	1	9.3030856	347.66667	5126.6667	0.93649151	1	9.3030918	-75.333333	4050.3333	1.0189518	
9.4030901	105	3238	0.96859109	1	9.4031188	328	4667	0.93433433	1	9.403125	-10	3718.6667	1.0026964	
9.5031229	263.66667	2671.6667	0.91017488	1	9.503152	400.66667	4348	0.91562544		9.5031582	0.33333333	3657.3333	0.99990887	
9.6031558	221.33333	2312.6667	0.91265456	1	9.6031851	477.66667	4413.3333	0.90233763		9.6031915	-25.666667	3570.6667	1.0072402	
9.7031887	286.33333	2763.3333	0.90610996	1	9.7032183	408	4660.3333	0.91950016		9.7032247	10	3876.3333	0.99742688	
9.8032216	48.333333	3049	0.98439518	1	9.8032515	313.33333	5230.6667	0.94348244		9.803258	272	4174.6667	0.93883058	
9.9032544	-37.666667	3233.6667	1.0117856	1	9.9032847	79.666667	4514.6667	0.9826598		9.9032912	301.33333	4086.6667	0.93132786	
10.003287	32	3029.6667	0.98954818	1	10.003318	-3.3333333	4310.3333	1.0007739		10.003324	242.33333	3985	0.94267466	
10.10332	2	2963	0.99932546	1	10.103351	320.33333	3599	0.91826841		10.103358	-45.333333	3649	1.0125798	
10.203353	217.66667	2712.3333	0.92571104	1	10.203384	536	4161	0.88588461		10.203391	-22.666667	3584	1.0063647	
10.303386	49	2748.3333	0.98248332	1	10.303417	519.33333	4606.3333	0.89867985		10.303424	98	3933.3333	0.97569043	
10.403419	197.33333	2793	0.93400959	1	10.403451	272.33333	5463	0.95251656		10.403457	56	4066.6667	0.98641656	
10.503452	57	2504.6667	0.97774886	1	10.503484	220.33333	5437.6667	0.96105809		10.503491	225.66667	4246.3333	0.94953787	

NCP + Int. + Mg traces					dynamic NCP trace 1				dynamic NCP trace 2				
0	279	2541	0.90106383	NCP + Int. + Mg	0	727.66667	1641.3333	0.69283805	0	109.33333	3187.6667	0.96683854	NCP
0.10003307	610.33333	3155.3333	0.83792157	FRET histogram	0.10003303	747.66667	2511.6667	0.77060749	0.10003322	303	2797.3333	0.90226857	proximal and distal
0.20006614	851	2998.3333	0.77892276	0.61	0.20006605	793.33333	2550.3333	0.76273552	0.20006645	357.33333	2812	0.88725284	FRET histogram
0.30009921	859	3077.3333	0.78177661	0.64	0.30009908	1182	2426.6667	0.6724552	0.30009967	318	2690.3333	0.89429363	0.15
0.40013228	851.66667	2734	0.76248025	0.65	0.4001321	981.66667	2037.6667	0.67487304	0.40013289	140	2730	0.95121951	0.15
0.50016534	1339.3333	2798.3333	0.6763071	0.68	0.50016513	1037.3333	2315.6667	0.69062531	0.50016611	185	2598.3333	0.93353293	0.17
0.60019841	1191.6667	2484.6667	0.67585457	0.68	0.60019815	790.66667	2306.6667	0.74472665	0.60019934	192	2852.3333	0.936932	0.2
0.70023148	1151.6667	2425.6667	0.6780656	0.7	0.70023118	933.66667	2349	0.71557677	0.70023256	320.66667	2745.3333	0.89541205	0.2
0.80026455	499.66667	2589.3333	0.83824323	0.73	0.8002642	998	2163.3333	0.68431042	0.80026578	332.66667	2938	0.89828781	0.2
0.90029762	453	2312.3333	0.83618611	0.73	0.90029723	948.33333	2258.6667	0.70429269	0.900299	243.33333	2906	0.92273497	0.2
1.0003307	433	2590	0.8567648	0.74	1.0003303	949.66667	2336	0.71096683	1.0003322	154	2832.3333	0.94843174	0.2
1.1003638	362	2469	0.87212999	0.75	1.1003633	722	2504	0.77619343	1.1003654	5	2770.3333	0.99819841	0.2
1.2003968	364.66667	2862	0.88698347	0.75	1.2003963	890.33333	2521.3333	0.73903273	1.2003987	140.33333	2770.6667	0.95179205	0.2
1.3004299	327.33333	3044.3333	0.90291646	0.75	1.3004293	859	2825.3333	0.76685063	1.3004319	195.66667	2986.6667	0.93851472	0.2
1.400463	377.66667	3271.6667	0.89651078	0.75	1.4004624	1039.3333	3011.6667	0.74343783	1.4004651	213.66667	2997.3333	0.93345791	0.22
1.500496	464	2966	0.86472303	0.75	1.5004954	829	3163.6667	0.79236934	1.5004983	116	3141.6667	0.96439169	0.22
1.6005291	742.66667	2613.6667	0.77872679	0.75	1.6005284	734	3128.6667	0.80997584	1.6005316	106.66667	3201	0.96775169	0.22
1.7005622	750	2698.3333	0.78250362	0.76	1.7005614	465.66667	3283	0.87577805	1.7005648	320.33333	2736	0.89519032	0.23
1.8005952	847.33333	3092.6667	0.78494078	0.76	1.8005945	402	3801.6667	0.9043692	1.800598	273	2672	0.90730051	0.24
1.9006283	688	2872.6667	0.80677776	0.77	1.9006275	479.66667	3894	0.89032848	1.9006312	284.66667	2689.6667	0.90429228	0.25
2.0006614	902	1912	0.67945984	0.78	2.0006605	507.33333	3937.6667	0.88586427	2.0006645	231.33333	3056.6667	0.92964315	0.25
2.1006944	742.33333	1775	0.70511123	0.78	2.1006935	428.66667	3264.3333	0.88392454	2.1006977	299.33333	2929.6667	0.90729844	0.25
2.2007275	594.66667	2088	0.77833002	0.8	2.2007266	301.33333	3242.3333	0.91496567	2.2007309	145.33333	2597	0.94700377	0.25
2.3007606	399	2878.3333	0.87825468	0.8	2.3007596	332	3258	0.90752089	2.3007641	-48	2713.6667	1.0180068	0.26
2.4007937	377.66667	2554.6667	0.87120609	0.8	2.4007926	458.66667	3244	0.87612532	2.4007973	14.333333	3182.6667	0.99551663	0.26
2.5008267	533.66667	2343.6667	0.81452734	0.8	2.5008256	518	2835.6667	0.84554219	2.5008306	311.66667	3995.6667	0.92764278	0.27
2.6008598	437.66667	2151.6667	0.83097322	0.8	2.6008587	391.66667	2651.3333	0.8712893	2.6008638	533.66667	4103.6667	0.88491949	0.27
2.7008929	486.33333	2535	0.83903354	0.8	2.7008917	502	2710	0.84371108	2.700897	504.66667	3587.6667	0.87667997	0.27
2.8009259	397	2644	0.86945084	0.8	2.8009247	398.33333	2884.6667	0.87866789	2.8009302	436	3223.3333	0.88085261	0.27
2.900959	408	2780.6667	0.87204683	0.81	2.9009577	489	2828.3333	0.85259244	2.9009635	354.33333	2941.6667	0.89249595	0.27
3.0009921	453	2609.6667	0.85208968	0.81	3.0009908	334.33333	3199.3333	0.90538628	3.0009967	220.66667	3182.3333	0.93515526	0.27
3.1010251	389.66667	2757.3333	0.87617837	0.82	3.1010238	293.33333	3265	0.9175644	3.1010299	76	3056.6667	0.97573952	0.27
3.2010582	607	2727.6667	0.81797281	0.82	3.2010568	208	3110.6667	0.93732423	3.2010631	137	3251.3333	0.95956714	0.27
3.3010913	701.66667	2486.6667	0.77992682	0.82	3.3010898	297.66667	2614.6667	0.897791	3.3010963	405.33333	3168.6667	0.88658832	0.29
3.4011243	781.66667	2668.6667	0.77345184	0.82	3.4011229	543.33333	2818.3333	0.83837382	3.4011296	506.66667	3043.6667	0.8572904	0.29
3.5011574	572	2744.3333	0.82752035	0.83	3.5011559	599.66667	2978	0.8323861	3.5011628	356	2891.3333	0.89037159	0.3
3.6011905	460.33333	3082	0.87004799	0.83	3.6011889	358	2944.6667	0.89160275	3.601196	76.666667	2913	0.97435612	0.3
3.7012235	397.66667	2851.6667	0.87761592	0.83	3.7012219	159	2914.6667	0.94827025	3.7012292	47.666667	2738.3333	0.98289064	0.3
3.8012566	477.33333	2591	0.84443237	0.83	3.801255	368	3148	0.89533561	3.8012625	331	2909.6667	0.89786052	0.3
3.9012897	377.66667	2958.3333	0.88679057	0.83	3.901288	613.33333	3456.6667	0.84930385	3.9012957	485.66667	3112.6667	0.86503011	0.3
4.0013228	439.66667	2746.6667	0.86201486	0.84	4.001321	574.66667	3426.6667	0.85638121	4.0013289	354.33333	3493	0.90790158	0.3
4.1013558	414.66667	2961.3333	0.8771722	0.84	4.101354	463.33333	3263.3333	0.87567084	4.1013621	109	3443	0.96931306	0.3
4.2013889	484.33333	2488.3333	0.83707109	0.84	4.2013871	764.66667	3420	0.8172694	4.2013953	189	3095	0.94244823	0.3
4.301422	809	2644.6667	0.7657562	0.84	4.3014201	695.33333	3708.6667	0.84211323	4.3014286	136	3060.3333	0.95745125	0.3
4.401455	772.66667	2478.6667	0.76235391	0.84	4.4014531	619.33333	3757	0.85848122	4.4014618	183.66667	2783	0.93808989	0.3
4.5014881	964	2669.3333	0.7346789	0.85	4.5014861	348.66667	3340.3333	0.90548477	4.501495	205.66667	2929	0.93438962	0.3
4.6015212	691.66667	2891.3333	0.80695879	0.85	4.6015192	684.66667	2762.6667	0.80139238	4.6015282	483.66667	2889.6667	0.85662055	0.3
4.7015542	862.33333	3134.6667	0.78425486	0.85	4.7015522	659.66667	2857.6667	0.81245262	4.7015615	573.33333	2767.3333	0.82837757	0.3
4.8015873	624	3066	0.83089431	0.85	4.8015852	500.66667	2962	0.85541009	4.8015947	345.66667	2396.6667	0.87395162	0.3
4.9016204	591.66667	2863.3333	0.8287506	0.85	4.9016182	243	3460.6667	0.93438934	4.9016279	370.66667	2567	0.87382276	0.3
5.0016534	325.33333	2768	0.89482759	0.85	5.0016513	271.66667	3256.6667	0.92300425	5.0016611	382	2858.6667	0.88212302	0.3
5.1016865	321	2806.6667	0.89736758	0.85	5.1016843	306.66667	3449.6667	0.9183601	5.1016944	381.66667	3255.6667	0.89506965	0.31
5.2017196	435.66667	2800.6667	0.86538263	0.86	5.2017173	375.66667	3101.3333	0.89195667	5.2017276	113.66667	3454.3333	0.96814275	0.31
5.3017526	629.66667	2923.3333	0.82277887	0.87	5.3017503	697	3395.6667	0.82969539	5.3017608	-9.3333333	3492.3333	1.0026797	0.32
5.4017857	762.33333	2723.6667	0.78131574	0.88	5.4017834	824	3366.6667	0.80337257	5.401794	144	3183.6667	0.95672643	0.32
5.5018188	453.66667	2749	0.85834721	0.88	5.5018164	909	3544.6667	0.79589851	5.5018272	412.33333	2649	0.86530923	0.32
5.6018519	261	2477.6667	0.90469815	0.89	5.6018494	742.33333	2806.3333	0.79081345	5.6018605	191.66667	2568.6667	0.93056394	0.33
5.7018849	24.333333	2224.6667	0.98918038	0.89	5.7018824	905	2716.6667	0.75011505	5.7018937	175	2660.3333	0.93827886	0.33
5.801918	105.66667	2167.6667	0.95351906	0.89	5.8019155	865.66667	2199.6667	0.71759461	5.8019269	-48.666667	2666.3333	1.0185916	0.33
5.9019511	191.33333	2067	0.91527675	0.89	5.9019485	1070.6667	2565	0.70551022	5.9019601	221	2762.6667	0.92593006	0.33
6.0019841	208	2426.6667	0.92105263	0.9	6.0019815	964	2198.3333	0.6951618	6.0019934	349.33333	2755	0.88746913	0.33
6.1020172	360	2486.3333	0.87352149	0.9	6.1020145	1102	2218.6667	0.66813893	6.1020266	499	2974	0.85632018	0.34
6.2020503	392	2862	0.87953288	0.9	6.2020476	927	1894.3333	0.67143195	6.2020598	336.66667	3006.6667	0.89930209	0.34
6.3020833	553.66667	2866.3333	0.83810916	0.9	6.3020806	1156.6667	1856.6667	0.61615044	6.302093	96.666667	3256.3333	0.9711701	0.35
6.4021164	539.66667	3014.3333	0.84815232	0.9	6.4021136	1183.6667	1840	0.60853269	6.4021262	-81.333333	3190.6667	1.0261578	0.35
6.5021495	747.33333	3183.6667	0.80988722	0.9	6.5021466	1325.3333	2075.3333	0.6102725	6.5021595	28	3032.6667	0.99085167	0.35
6.6021825	788	3234.3333	0.80409381	0.9	6.6021797	1203	2246	0.65120325	6.6021927	59.333333	2907.6667	0.98000225	0.35
6.7022156	787.66667	3021.3333	0.79320907	0.9	6.7022127	1186.3333	2564.3333	0.68370068	6.7022259	79.333333	3170.6667	0.97558974	0.35
6.8022487	523	2971.3333	0.8503291	0.9	6.8022457	1083	2694	0.7132645	6.8022591	-29	3279.6667	1.0089212	0.35
6.9022817	400.33333	2816.3333	0.87554404	0.9	6.9022787	1108	3018.3333	0.73148073	6.9022924	-16.666667	3477	1.0048165	0.35
7.0023148	355.33333	2719.3333	0.88443192	0.9	7.0023118	1016.3333	3056.3333	0.75045016	7.0023256	212	3469.6667	0.94241738	0.35
7.1023479	430.66667	2750	0.86459862	0.91	7.1023448	879.66667	3058.6667	0.77663986	7.1023588	402.66667	3443.3333	0.89530248	0.35
7.202381	670	3193	0.82655967	0.91	7.2023778	838.33333	3035.3333	0.78358145	7.202392	459	3376	0.88031291	0.35
7.302414	689	3509.3333	0.83588726	0.91	7.3024108	990.66667	2949.6667	0.74858303	7.3024252	355.66667	3087	0.89668861	0.35
7.4024471	694.33333	3325	0.82725162	0.91	7.4024439	1116	2761.6667	0.71219806	7.4024585	184	3128	0.94444444	0.35
7.5024802	498.33333	3196.3333	0.86512089	0.91	7.5024769	979	2704.6667	0.7342322	7.5024917	243.33333	2999	0.92495117	0.36
7.6025132	479.33333	3287	0.8727321	0.92	7.6025099	894	2626.6667	0.74607082	7.6025249	280.33333	3025	0.91518758	0.36
7.7025463	439.33333	3532	0.88937385	0.92	7.7025429	922	2429	0.72485825	7.7025581	325	2984	0.90178302	0.36
7.8025794	496.33333	3255.6667	0.867715	0.92	7.802576	836.33333	2115.6667	0.71668925	7.8025914	170	3632	0.95528669	0.36
7.9026124	663	2907.6667	0.81432039	0.92	7.902609	886	2313.3333	0.72306731	7.9026246	285	4054.3333	0.93432171	0.36
8.0026455	619	2536.6667	0.80384494	0.92	8.002642	869.33333	2414.6667	0.73528218	8.0026578	137.66667	3956	0.96637082	0.36
8.1026786	612.66667	2420.3333	0.79799978	0.93	8.102675	1002.3333	2557	0.71839296	8.102691	247	3417.6667	0.9325996	0.37
8.2027116	274.33333	2499.6667	0.9011055	0.93	8.2027081	875	2297	0.7241488	8.2027243	142.66667	3278.3333	0.95829679	0.37
8.3027447	219.33333	2450.6667	0.91785268	0.94	8.3027411	737.66667	2596.3333	0.77874425	8.3027575	519.66667	3911.6667	0.88272905	0.37
8.4027778	96.666667	2669	0.96504761	0.94	8.4027741	787.66667	2488.6667	0.75958897	8.4027907	419.33333	3934.3333	0.90368272	0.37
8.5028108	187	2945.6667	0.94030645	0.94	8.5028071	957.33333	2555.6667	0.72748838	8.5028239	418.66667	3974	0.90468963	0.37
8.6028439	355	2806.3333	0.88770561	0.95	8.6028402	1013.3333	2761.6667	0.73156733	8.6028571	107.66667	3311.6667	0.96851238	0.37
8.702877	516	2643.6667	0.83669163	0.95	8.7028732	1211.3333	2737.3333	0.69322978	8.7028904	156	3568	0.95810956	0.37
8.8029101	508.33333	2193.6667	0.81186775	0.95	8.8029062	847.33333	3034	0.78169014	8.8029236	72	3535	0.98003881	0.37
8.9029431	602.66667	2365.6667	0.796968	0.95	8.9029392	772.66667	2775	0.78220427	8.9029568	38.333333	3631.6667	0.98955495	0.38
9.0029762	505.33333	2668.6667	0.84078975	0.95	9.0029723	399.33333	2911.6667	0.87939193	9.00299	10.333333	3445.6667	0.99701003	0.38
9.1030093	734	2663.6667	0.78396939	0.95	9.1030053	559.33333	2619.6667	0.82405369	9.1030233	0.66666667	3172.6667	0.99978992	0.38
9.2030423	701	3018.3333	0.81152536	0.95	9.2030383	587.66667	3087.3333	0.8400907	9.2030565	7.3333333	3166	0.99768908	0.38
9.3030754	695.33333	2699.6667	0.795189	0.95	9.3030713	595.33333	3320.3333	0.84796118	9.3030897	36.333333	2868.3333	0.98749139	0.38
9.4031085	510	2884	0.84973483	0.95	9.4031044	403.33333	3650	0.90049342	9.4031229	156	2812.6667	0.94745116	0.39
9.5031415	338.33333	2540	0.88245512	0.95	9.5031374	267.66667	3415.6667	0.92733032	9.5031561	174	3085.3333	0.94661485	0.39
9.6031746	549.66667	2740.3333	0.83292806	0.95	9.6031704	257	3442.3333	0.93052802	9.6031894	233	3187.3333	0.93187798	0.39
9.7032077	579.66667	2547	0.81460554	0.96	9.7032034	392	3169.6667	0.88993917	9.7032226	1.6666667	3578	0.99953441	0.4
9.8032407	852	2426.3333	0.74011185	0.96	9.8032365	681.33333	3172	0.82318339	9.8032558	171.33333	3517.6667	0.95355562	0.4
9.9032738	943.33333	2272	0.70661414	0.96	9.9032695	593	2729	0.82149308	9.903289	8.3333333	3602.6667	0.99769224	0.4
10.003307	970.66667	2259	0.69945299	0.96	10.003303	586.33333	2910.6667	0.83233248	10.003322	88.333333	3440.3333	0.97496694	0.4
10.10334	879.66667	2223	0.71648045	0.96	10.103336	278.33333	3011	0.91538306	10.103355	-29.666667	3611.6667	1.0082822	0.4
10.203373	667.33333	2302.3333	0.77528342	0.97	10.203369	362.66667	3330	0.90178733	10.203389	1.6666667	3365.6667	0.99950505	0.4
10.303406	943.33333	2299.3333	0.70908717	0.97	10.303402	287.66667	3556.6667	0.92517125	10.303422	-34.666667	3279.3333	1.0106842	0.4
10.403439	771.33333	2434.3333	0.75938442	0.97	10.403435	238.66667	3216.3333	0.93092137	10.403455	42	2937.6667	0.98590446	0.4
10.503472	854.33333	2330.3333	0.7317354	0.98	10.503468	126.33333	3192.3333	0.9619325	10.503488	88.333333	3115.6667	0.9724303	0.4

10.603485	38.333333	2442.6667	0.98454924	1	10.603517	253	5402.3333	0.95526347	10.603524	58	4048.3333	0.98587548	10.603505	676.33333	2633.3333	0.79564911	0.98	10.603501	346.66667	2682.3333	0.88555079	10.603522	137.66667	2968	0.95567243	0.4
10.703517	183.66667	2274.3333	0.925278		10.70355	94	4514	0.97960069	10.703557	14	4365.3333	0.99680317	10.703538	814	2511.6667	0.75523705	0.98	10.703534	332	2894	0.89708617	10.703555	-28.666667	3076	1.0094071	0.4
10.80355	352.66667	2708.3333	0.88478711		10.803583	84.666667	4403	0.98113348	10.80359	-170.33333	4554.6667	1.0388505	10.803571	716.33333	2503.6667	0.77753623	0.98	10.803567	452.66667	3057.3333	0.87103514	10.803588	14.666667	2989.6667	0.99511816	0.41
10.903583	438.66667	2925	0.86958676		10.903616	364.33333	3815.3333	0.91283196	10.903624	-200	4850.3333	1.0430077	10.903604	751	1976	0.72460579	0.99	10.9036	286	3421	0.92284866	10.903621	152.66667	3355.3333	0.95648043	0.41
11.003616	307.33333	3664	0.92261205		11.00365	437.66667	4563.6667	0.91249	11.003657	50	4271.6667	0.98843039	11.003638	659	1950.6667	0.74747733	0.99	11.003633	429	3401.6667	0.88800905	11.003654	286	3033.3333	0.91383812	0.41
11.103649	260.33333	3631	0.93309919		11.103683	452	4483	0.90840932	11.10369	65.666667	4200.6667	0.98460817	11.103671	608	1864.6667	0.75411162	1	11.103666	376	3717.3333	0.90814332	11.103688	174	3173.3333	0.94801832	0.41
11.203682	256	3653.6667	0.93452127		11.203716	328.66667	5631	0.9448515	11.203723	29	4035.3333	0.99286476	11.203704	263	2465.6667	0.90361593	1	11.203699	417.33333	3627	0.89681035	11.203721	-49.333333	2843.3333	1.0176569	0.41
11.303715	343	3189.3333	0.90289705		11.303749	301	5392.3333	0.94713115	11.303757	-66.666667	4041	1.0167743	11.303737	343	2605	0.88364993	1	11.303732	347.66667	3555.6667	0.91093083	11.303754	-247.33333	3148.6667	1.0852482	0.42
11.403748	419.66667	3657	0.89705642		11.403782	250.33333	5030	0.95259138	11.40379	-137	3931.6667	1.0361033	11.40377	409.66667	2603.6667	0.86404867	1	11.403765	339.66667	3283	0.9062385	11.403787	-275.66667	3255	1.0925263	0.42
11.50378	394.66667	3751.3333	0.90480785		11.503816	185	4058.3333	0.9564022	11.503823	-99.333333	3595.3333	1.0284134	11.503803	441.66667	2420	0.84566104	1	11.503798	343	3319	0.90633534	11.503821	-129.66667	3051	1.0443861	0.42
11.603813	380.66667	3665.3333	0.90591531		11.603849	135.66667	3915.3333	0.96651033	11.603856	-94.333333	3648.6667	1.0265404	11.603836	348.33333	2515.3333	0.87836108		11.603831	483.66667	3280	0.87149057	11.603854	163	2912	0.94699187	0.42
11.703846	198	3258.3333	0.94271386		11.703882	4	4545	0.99912069	11.70389	-45.333333	3817	1.0120194	11.703869	303.66667	2285.3333	0.8827089		11.703864	499.33333	3196	0.86487462	11.703887	195.66667	2564.6667	0.92911484	0.42
11.803879	323.33333	3269	0.9099935		11.803915	21.333333	4729.6667	0.99550972	11.803923	53	4162.6667	0.98742785	11.803902	456	2182	0.82714177		11.803897	465.33333	2945.3333	0.86356529	11.80392	47.333333	2802	0.98338793	0.42
11.903912	229.66667	3671.6667	0.94113124		11.903948	90.666667	4438	0.97997939	11.903956	274.33333	4209	0.93881041	11.903935	315	2041.3333	0.86631773		11.90393	472.33333	2460.6667	0.83895897	11.903953	-172.66667	2824	1.0651245	0.42
12.003945	203.33333	3895.6667	0.95039441		12.003981	2.3333333	3649.6667	0.99936108	12.003989	174.66667	4189.6667	0.95997861	12.003968	320.66667	2311.3333	0.87816616		12.003963	506	2446	0.82859079	12.003987	159.33333	2967	0.94903508	0.43
12.103978	4.6666667	3236.3333	0.99856012		12.104015	145.33333	4137.6667	0.9660674	12.104023	142.33333	4010	0.96572208	12.104001	327.66667	2821	0.89593479		12.103996	428.66667	2894	0.87098716	12.10402	304	3232.3333	0.91403525	0.43
12.204011	67.333333	3094.6667	0.97870546		12.204048	148	4638	0.96907647	12.204056	-239	4229	1.0598997	12.204034	570	2905.3333	0.83598696		12.204029	257.66667	3205.3333	0.92559438	12.204053	431	3237.3333	0.88250795	0.44
12.304043	214	3196.6667	0.93725567		12.304081	501.66667	4826	0.90583745	12.304089	22.666667	4726.3333	0.99522707	12.304067	589	3077.3333	0.83934903		12.304062	151	3564.6667	0.95936126	12.304086	173	3152	0.94796992	0.44
12.404076	258.66667	3482	0.93085012		12.404114	330.33333	4517	0.93185256	12.404122	44.666667	4580.3333	0.99034234	12.404101	529.33333	2456	0.8226887		12.404095	274	3307	0.92348506	12.40412	330.66667	2529.3333	0.88438228	0.45
12.504109	305.66667	3438	0.91835099		12.504147	271	4567.6667	0.94399284	12.504156	261.66667	4921	0.94951119	12.504134	586	2541.3333	0.81261991		12.504128	521.66667	3211	0.8602429	12.504153	280.33333	2556.3333	0.90117509	0.45
12.604142	330.33333	3101.3333	0.90373968		12.60418	135	5432.6667	0.97575286	12.604189	128.66667	4757.3333	0.97366626	12.604167	550	2426.6667	0.81522956		12.604161	706	2786	0.7978236	12.604186	326	2858.6667	0.8976345	0.45
12.704175	402.33333	3210	0.88862231		12.704214	481	5507	0.91967268	12.704222	328.66667	5141.3333	0.93991469	12.7042	523.33333	2594	0.83212147		12.704194	676.66667	2964.3333	0.81415362	12.704219	202.66667	3530	0.94570459	0.45
12.804208	455	2899.6667	0.86436804		12.804247	671.66667	5912.6667	0.89799018	12.804255	258.33333	4292.3333	0.94323176	12.804233	273.33333	2450.6667	0.89965737		12.804227	624.66667	3261.6667	0.8392658	12.804252	264	3683.3333	0.93311941	0.45
12.904241	524.33333	2719.6667	0.83836827		12.90428	719	5213	0.87879299	12.904289	98.666667	3999.6667	0.97592517	12.904266	190.33333	2331	0.92451084		12.90426	604.33333	3278.3333	0.84435096	12.904286	245	3575	0.93586387	0.45
13.004274	313.33333	2769	0.89834541		13.004313	504.66667	4996.3333	0.9082591	13.004322	-180	3658.3333	1.0517489	13.004299	226.66667	2000.6667	0.89823406		13.004293	489	3133.3333	0.86500414	13.004319	94.333333	3128.3333	0.97072818	0.45
13.104306	51.666667	2668	0.98100257		13.104346	675	3913	0.85287707	13.104355	-262	3980.6667	1.0704554	13.104332	439.66667	2331	0.84131376		13.104326	380	2914.6667	0.88466208	13.104352	-0.33333333	3086.6667	1.000108	0.45
13.204339	-127.66667	3171	1.0419496		13.20438	545.66667	4226.6667	0.8856604	13.204388	-200.66667	3592.6667	1.0591588	13.204365	364	2576	0.87619048		13.204359	393.33333	3168.3333	0.88956481	13.204385	-58	3315.6667	1.0178042	0.46
13.304372	184.66667	3112	0.94398382		13.304413	390	4563.3333	0.92126514	13.304422	-238.33333	3478	1.0735672	13.304398	572.33333	2971	0.83847601		13.304392	464.33333	3324	0.87743071	13.304419	307.33333	3467.3333	0.91858001	0.46
13.404405	347.66667	3380	0.90673343		13.404446	-81	5649.6667	1.0145457	13.404455	-289	3403.6667	1.0927868	13.404431	352	2910.3333	0.89210177		13.404425	599	3362	0.84877556	13.404452	394.66667	3760.6667	0.90502166	0.47
13.504438	341.33333	3020.6667	0.89847313		13.504479	-123.66667	5162	1.0245452	13.504488	-200.33333	3486.6667	1.0609595	13.504464	462	3088.6667	0.86988359		13.504458	606	3493.6667	0.8521831	13.504485	477	3703.6667	0.88590336	0.48
13.604471	125.66667	2918.3333	0.9587166		13.604512	-102	4967.6667	1.0209632	13.604521	-43	3372.3333	1.0129155	13.604497	193.33333	2752.3333	0.93436687		13.604491	513.66667	3266.3333	0.86410935	13.604518	337.66667	4014	0.92240521	0.49
13.704504	58.666667	3118.6667	0.98153588		13.704545	64.333333	4713	0.98653363	13.704555	161.33333	3740	0.95864662	13.70453	406	2459	0.8582897		13.704524	373.33333	3434.3333	0.9019522	13.704551	261.66667	4031.3333	0.93904806	0.49
13.804536	71.666667	3019	0.97681191		13.804579	43	4877	0.99126016	13.804588	211.66667	3789.3333	0.94709656	13.804563	514.66667	2590	0.83422804		13.804557	286.33333	3203	0.91794039	13.804585	284	3864.6667	0.93154427	0.49
13.904569	112.66667	3452.3333	0.96839645		13.904612	153	4837	0.96933868	13.904621	43.666667	3923.6667	0.98899345	13.904597	588	2791.3333	0.82600118		13.90459	308.66667	3628.3333	0.92159851	13.904618	43	3297.3333	0.98712703	0.5
14.004602	299	3153	0.91338355		14.004645	295.33333	4393.3333	0.93701123	14.004654	94.333333	3686.3333	0.97504849	14.00463	683	2714.3333	0.79895997		14.004624	458	3783	0.8920066	14.004651	249	2749	0.91694463	0.5
14.104635	129	3290.6667	0.96227703		14.104678	330.33333	4914.6667	0.93701938	14.104688	206.33333	3963.6667	0.95051958	14.104663	797	2073	0.72229965		14.104657	341.33333	3966	0.9207553	14.104684	86	2696.3333	0.96909069	0.5
14.204668	66.333333	3264	0.98008207		14.204711	140.66667	4939.3333	0.97230971	14.204721	410.66667	3795.3333	0.90236171	14.204696	784.66667	2041	0.72230742		14.20469	698.33333	3985	0.85088968	14.204718	396.33333	2652.3333	0.86999781	0.52
14.304701	3.6666667	3497	0.99895258		14.304745	-86	5198.3333	1.0168221	14.304754	182.33333	3854.6667	0.95483445	14.304729	705	2349.6667	0.76920559		14.304723	547.66667	3936	0.87785295	14.304751	208	2671.3333	0.92776106	0.6
14.404734	19.666667	3748.3333	0.99478061		14.404778	-122.33333	4917.3333	1.0255127	14.404787	-39.333333	3774.6667	1.0105301	14.404762	643.33333	2594	0.80127677		14.404756	463.66667	3747.3333	0.88989155	14.404784	197.66667	2247.6667	0.91916576	0.6
14.504767	-80.666667	3556.3333	1.023209		14.504811	-50.333333	5186.3333	1.0098001	14.50482	-63.333333	3948.3333	1.016302	14.504795	751	2832	0.79039911		14.504789	282.66667	3323	0.92160488	14.504817	89.333333	2690	0.967858	0.6
14.604799	-101	3702.6667	1.0280426		14.604844	141.33333	4670.3333	0.97062695	14.604854	-43.666667	3882.3333	1.0113755	14.604828	542.66667	2584.3333	0.82645773		14.604822	244	3260.3333	0.93037192	14.60485	114.66667	2730.6667	0.95970009	0.6
14.704832	1.6666667	3132.6667	0.99946825		14.704877	262.33333	4323	0.9427886	14.704887	140.66667	4015.6667	0.96615607	14.704861	491.33333	2548	0.83834174		14.704855	471.33333	3597.3333	0.88415533	14.704884	136.33333	3241	0.95963285	0.65
14.804865	225.33333	3235.3333	0.9348873		14.80491	329.66667	4150	0.92640821	14.80492	147.66667	4502	0.96824145	14.804894	435.66667	2736	0.86263794		14.804888	358.33333	3639.3333	0.91036438	14.804917	19	3075	0.99385908	0.68
14.904898	258.33333	3095.3333	0.92296988		14.904944	2	4184.3333	0.99952225	14.904953	62	4490	0.98637961	14.904927	585.33333	2710.6667	0.822411		14.904921	449	3423	0.88403926	14.90495	-64.666667	3108.6667	1.021244	0.7
15.004931	375	3416.3333	0.90109021		15.004977	129.66667	4264.3333	0.97049006	15.004987	-4.3333333	4470.6667	1.0009702	15.00496	507.33333	2708.6667	0.8422471		15.004954	403	3237.3333	0.88929585	15.004983	-50.666667	3457.6667	1.0148713	0.72
15.104964	407	3609	0.89865538		15.10501	185.66667	4455.3333	0.95999425	15.10502	7.6666667	4067.6667	0.99811876	15.104993	380.66667	2260.3333	0.85586268		15.104987	445.66667	3013	0.87114495	15.105017	64	3403.3333	0.98154201	0.73
15.204997	289.66667	3678.3333	0.92699933		15.205043	401	4686.6667	0.92118194	15.205053	117.66667	3984	0.97131247	15.205026	482.66667	2477.6667	0.8369553		15.20502	512	3182.3333	0.86140937	15.20505	196.66667	3194	0.94199764	0.74
15.30503	181.66667	3620	0.95221394		15.305076	174.33333	5097.3333	0.96693013	15.305086	-28.333333	3713.6667	1.0076881	15.30506	484	2636.3333	0.84488837		15.305053	466.66667	3169.3333	0.87165383	15.305083	272	2709	0.90875545	0.75
15.405062	55.333333	3430.6667	0.98412698		15.405109	189	4799.6667	0.96211413	15.40512	-118.66667	3960.6667	1.0308867	15.405093	666.33333	2825.3333	0.80916468		15.405086	424.33333	3737.6667	0.89804581	15.405116	202	2492.3333	0.92502784	0.75
15.505095	45	3348.3333	0.9867387		15.505143	142.33333	5081	0.97275048	15.505153	50.333333	4175	0.98808772	15.505126	409	2821.3333	0.87338768		15.505119	411.33333	4068.3333	0.90817769	15.50515	185.33333	2524.6667	0.93161132	0.75
15.605128	-80.333333	3710	1.0221324		15.605176	132	4860	0.97355769	15.605186	135	4384.6667	0.97013054	15.605159	493.33333	2707.6667	0.8458815		15.605152	410.33333	3809.3333	0.90275693	15.605183	106.33333	2825.6667	0.96373352	0.76
15.705161	-81	3523.6667	1.0235283		15.705209	121	5455.3333	0.97830115	15.705219	260.66667	4121	0.9405097	15.705192	428.33333	2538	0.85560175		15.705185	575	3501	0.85893032	15.705216	-80.666667	3324.6667	1.0248664	0.76
15.805194	-6	3402.6667	1.0017664		15.805242	66.333333	5382.3333	0.98782577	15.805253	167.66667	3969	0.95946817	15.805225	433.66667	2409.6667	0.84747948		15.805218	724.33333	3173	0.81414642	15.805249	-50.333333	3367	1.0151759	0.76
15.905227	217.33333	3233	0.93701092		15.905275	196.33333	5255.6667	0.96398875	15.905286	300.33333	3760	0.92603235	15.905258	532	2670	0.83385384		15.905251	841	3489	0.80577367	15.905282	98.333333	2922.6667	0.96745007	0.77
16.00526	259	3268.6667	0.92658036		16.005309	207.33333	4606.3333	0.95692819	16.005319	139.33333	3910	0.96559104	16.005291	490.66667	2788	0.85034567		16.005284	640.33333	3183	0.83251962	16.005316	228.33333	2447	0.91465238	0.78
16.105293	104.33333	3285.3333	0.96922018		16.105342	420.66667	5051	0.9231191	16.105352	146	4277.6667	0.9669957	16.105324	756.33333	2776	0.7858828		16.105317	365.66667	3305.6667	0.90039949	16.105349	75.333333	2890	0.97459532	0.79
16.205325	214.66667	3265.6667	0.93832008		16.205375	672.33333	5118.6667	0.88390031	16.205386	-93	4029.6667	1.023624	16.205357	735.33333	2280.6667	0.75618921		16.20535	234.66667	3131	0.93027632	16.205382	-18	3258.3333	1.005555	0.8
16.305358	160.33333	3071.6667	0.95039191		16.305408	709.33333	5497.3333	0.88571429	16.305419	-167.33333	3889.3333	1.0449579	16.30539	795	2101.6667	0.72554661		16.305383	218	3260	0.9373203	16.305415	-98.333333	3781	1.0267017	0.8
16.405391	415	3008.3333	0.87877313		16.405441	602.33333	5586.6667	0.90267679	16.405452	-315.66667	3440.6667	1.1010133	16.405423	796	1881.6667	0.70272625		16.405416	83	3009	0.97315653	16.405449	-21.666667	3605	1.0060465	0.82
16.505424	317	2700	0.89492874		16.505474	533.33333	5824.6667	0.91611618	16.505485	-125.33333	3847.3333	1.0336737	16.505456	622.33333	2096	0.7710607		16.505449	174.66667	3224.3333	0.94861234	16.505482	19.333333	4003.3333	0.9951939	0.85
16.605457	433	2904.6667	0.87026865		16.605508	453	5921	0.92893003	16.605519	-98.666667	3632	1.0279245	16.605489	479	2416.3333	0.83456136		16.605482	225	3189.3333	0.93410134	16.605515	140.66667	3883.6667	0.96504597	0.85
16.70549	407.33333	2913.6667	0.87734618		16.705541	476.66667	5163.6667	0.91548963	16.705552	51.333333	3621	0.9860216	16.705522	294.33333	2510.3333	0.89505586		16.705515	371.33333	3283.6667	0.89840401	16.705548	235.33333	3870.6667	0.9426855	0.87
16.805523	452.66667	3820	0.89405523		16.805574	531.66667	4908	0.90226117	16.805585	135.66667	3657.6667	0.9642355	16.805556	429	2509.3333	0.85399887		16.805548	340.33333	3039	0.8992898	16.805581	149	3300.3333	0.95680325	0.87
16.905556	667	3837	0.85190941		16.905607	791.33333	4342.6667	0.84586417	16.905618	64.333333	3782	0.98327411	16.905589	637.66667	1985	0.75686324		16.905581	351.66667	3271.6667	0.90294388	16.905615	480	3407	0.87651145	0.87
17.005588	558.33333	3941	0.87590754		17.00564	826.66667	4090	0.83186441	17.005652	152	4098	0.96423529	17.005622	799	2123.6667	0.72661953		17.005614	311.33333	3510	0.91852756	17.005648	522.66667	3237.6667	0.86100523	0.87
17.105621	743.66667	3312.3333	0.81665023		17.105674	716.66667	4245.6667	0.85557869	17.105685	-5.6666667	3998.6667	1.0014192	17.105655	607	2204.3333	0.78408821		17.105647	253.33333	3630.3333	0.93476955	17.105681	742.33333	3269.6667	0.81497175	0.88
17.205654	601.33333	3283.3333	0.84520336		17.205707	449.66667	4309.3333	0.90551236	17.205718	86.666667	4148.3333	0.97953562	17.205688	613.66667	2557.6667	0.80649569		17.20568	222.33333	3895.3333	0.94600502	17.205714	337.33333	3076.3333	0.90118153	0.88
17.305687	542.66667	3566	0.86792147		17.30574	412.66667	4935.6667	0.92284201	17.305751	-64	3884.3333	1.0167525	17.305721	459.33333	2475	0.84346246		17.305713	336.66667	4135.3333	0.92471676	17.305748	218.66667	3192.6667	0.93589994	0.89
17.40572	329	3545.6667	0.91508947		17.405773	402.33333	4481.3333	0.91761654	17.405785	189	3746.3333	0.95197357	17.405754	481.66667	2237	0.82282982		17.405746	280.33333	4090.3333	0.93586028	17.405781	136.33333	3135.6667	0.95833333	0.9
17.505753	113.66667	3604	0.96942527		17.505806	843	4993.3333	0.85556	17.505818	354.33333	4028.3333	0.9191512	17.505787	373	2294.3333	0.86015996		17.505779	457	3682.6667	0.88960464	17.505814	201	2878.3333	0.93472613	0.9
17.605786	76.333333	3202.6667	0.97672054		17.605839	932	4818	0.83791304	17.605851	283.33333	4246.6667	0.93745401	17.60582	507.66667	2063.3333	0.80254116		17.605812	352	3710.6667	0.9133574	17.605847	255	3106	0.92412972	0.9
17.705819	244	3302	0.93119007		17.705873	1001	5022.6667	0.83382215	17.705884	87	4427	0.98072663	17.705853	557	1811.3333	0.76481351		17.705845	377.33333	4088	0.91549716	17.70588	452.33333	3295.6667	0.87931341	0.9
17.805851	319.66667	3488	0.91604657		17.805906	497	4806.3333	0.90628536	17.805918	-13	3832.3333	1.0034037	17.805886	903.33333	1729.6667	0.6569186		17.805878	206.33333	4549	0.95661012	17.805914	419	3375.3333	0.88957217	0.9
17.905884	669	3280.3333	0.83060432		17.905939	215	4708.3333	0.9563304	17.905951	145	3602.6667	0.96130926	17.905919	717	2239	0.75744249		17.905911	172.66667	4308.6667	0.9614698	17.905947	345	3099.6667	0.89984517	0.9
18.005917	373	3174.6667	0.89486047		18.005972	164.33333	4869	0.96735099	18.005984	-47	3506	1.0135877	18.005952	887	2484.6667	0.73692536		18.005945	188.33333	3587.6667	0.95012359	18.00598	180	2969.6667	0.9428511	0.9
18.10595	449.33333	2738.6667	0.85905479		18.106005	60.666667	4280	0.98602365	18.106017	-10.666667	3729.6667	1.0028682	18.105985	680.66667	2388	0.77818814		18.105978	159.66667	3304.3333	0.95390685	18.106013	340	3451	0.9103139	0.91
18.205983	123.66667	3040.6667	0.96091857		18.206038	236	4635.6667	0.95155662	18.206051	-72	3772	1.0194595	18.206019	840.66667	2357.6667	0.73715477		18.206011	359.66667	3438.3333	0.90530104	18.206047	476.33333	3506.3333	0.88039839	0.91
18.306016	367.33333	2923	0.88835984		18.306072	445	4735	0.91409266	18.306084	58.333333	3739.3333	0.98463969	18.306052	827.66667	2637.6667	0.76115814		18.306044	423	3697.3333	0.8973384	18.30608	406	3478.6667	0.89548653	0.92
18.406049	152.33333	3153.6667	0.95392216		18.406105	629.33333	5128	0.89069013	18.406117	23.333333	3547.3333	0.99346527	18.406085	846	2824.6667	0.76952416		18.406077	555	3972	0.87740225	18.406113	187.33333	2941.3333	0.94012359	0.92
18.506082	474.33333	2896.6667	0.85929002		18.506138	532.66667	5048.3333	0.90455713	18.50615	14	3957	0.99647444	18.506118	823	2772.6667	0.77111338		18.50611	438	3711	0.89443239	18.506146	275.66667	2767.3333	0.90940957	0.92
18.606114	168	2701.6667	0.94145662		18.606171	82.666667	4840.6667	0.98320921	18.606184	-64	4159	1.0156288	18.606151	1028.6667	2265.6667	0.68774664		18.606143	610.66667	3921.6667	0.8652644	18.606179	331.33333	2895	0.89730344	0.93
18.706147	412.33333	2636.3333	0.86474962		18.706204	13	4352	0.99702176	18.706217	-86	4081	1.0215269	18.706184	777	2491.3333	0.76226415		18.706176	617.66667	3853.3333	0.86185044	18.706213	695	2928.6667	0.80820532	0.93
18.80618	137.66667	2819	0.95343856		18.806238	236.33333	3924	0.94319365	18.80625	-206.33333	4046.6667	1.053728	18.806217	631.66667	2314	0.78556071		18.806209	674	3588	0.84185828	18.806246	405.66667	3040.3333	0.88227897	0.94
18.906213	187.33333	2809.3333	0.9374861		18.906271	190.66667	4439.3333	0.95881929	18.906283	-231.33333	4026.3333	1.0609574	18.90625	295.66667	2613	0.89834976		18.906242	500.33333	3299.3333	0.86832178	18.906279	256.66667	2853	0.91746168	0.94
19.006246	-3.6666667	2482.3333	1.0014793		19.006304	372.33333	5153.6667	0.93262155	19.006316	-338.33333	4511.6667	1.0810703	19.006283	410.33333	2614.6667	0.86435262		19.006275	249.33333	2998.3333	0.92322693	19.006312	-67.666667	3016.3333	1.0229482	0.94
19.106279	42	2519.3333	0.98360229		19.106337	491.33333	5577	0.91903323	19.10635	-100.33333	4174	1.0246297	19.106316	563	2733.3333	0.82920417		19.106308	96	3099	0.96995305	19.106346	-100.33333	2827.3333	1.0367926	0.94
19.206312	161	3189.6667	0.95194986		19.20637	553.66667	5220.3333	0.90411038	19.206383	51	3760.3333	0.98661886	19.206349	476	3019.3333	0.86381842		19.206341	40.666667	3415.3333	0.98823302	19.206379	-69.666667	2755	1.0259434	0.94
19.306345	428	3495.6667	0.89091836		19.306403	340	5347.3333	0.94021803	19.306416	210.66667	3507	0.94333363	19.306382	527	2699.6667	0.83667355		19.306374	457.33333	3494	0.88425848	19.306412	-109	2849.3333	1.0397762	0.94
19.406377	422.66667	3374.3333	0.88868405		19.406437	85.666667	5116.3333	0.98353197	19.406449	171	3363.6667	0.95162203	19.406415	537.66667	2904.3333	0.84379237		19.406407	531	3568.6667	0.87047727	19.406445	93.333333	3280.6667	0.97233748	0.94
19.50641	349	3334	0.90524029		19.50647	257.66667	4777.6667	0.94882828	19.506483	72	3868	0.98172589	19.506448	509.66667	2574.6667	0.8347563		19.50644	522	3730	0.87723424	19.506478	2.6666667	3360	0.99920698	0.94
19.606443	332	3402	0.91108731		19.606503	487.66667	4574.6667	0.90366761	19.606516	-11	4137.3333	1.0026658	19.606481	393.66667	2581.3333	0.86767507		19.606473	205	3436.3333	0.94370194	19.606512	28	3476.3333	0.99200989	0.95
19.706476	318	3116.3333	0.90740561		19.706536	648.66667	5057	0.88631185	19.706549	-64	4478	1.0144993	19.706515	221.66667	2148	0.9064566		19.706506	127.33333	3829	0.96781532	19.706545	-135	3577	1.0392214	0.95
19.806509	450	3025	0.8705036		19.806569	641	5025.6667	0.88688235	19.806582	-62	4388.6667	1.0143297	19.806548	519.66667	2180	0.8075071		19.806539	182.66667	3707.3333	0.95304199	19.806578	70.333333	3673	0.98121104	0.95
19.906542	409.66667	3365	0.89146945		19.906603	521.33333	4899	0.90381895	19.906616	30.333333	3913.3333	0.99230834	19.906581	572.33333	2118.3333	0.7872894		19.906572	392	4182	0.91429821	19.906611	256	3769	0.93639752	0.95
20.006575	603	3978	0.86836935		20.006636	490	4599	0.90371389	20.006649	51	3476.6667	0.98554285	20.006614	716	2188.6667	0.75350011		20.006605	541.66667	3965	0.87980769	20.006644	502.66667	3543.6667	0.8757723	0.95
20.106607	509.66667	3962	0.88602311		20.106669	401.66667	5001	0.925654	20.106682	96	3462.3333	0.97302108	20.106647	437.66667	2209.6667	0.8346764		20.106638	526.33333	3632.3333	0.873437	20.106678	486.33333	3297.6667	0.87147639	0.95
20.20664	358	3738.6667	0.91261188		20.206702	309.33333	5058.6667	0.94237457	20.206715	26.666667	3902	0.99321229	20.20668	416.66667	2343.6667	0.84905205		20.206671	243	3495.3333	0.93499777	20.206711	280.66667	3289.6667	0.92138923	0.95
20.306673	67.666667	3573.6667	0.98141706		20.306735	237	4938.6667	0.9542088	20.306749	11.666667	4210	0.99723648	20.306713	474.66667	2385.6667	0.83405198		20.306704	188.33333	3302.3333	0.9460466	20.306744	140.33333	3221.6667	0.95825897	0.95
20.406706	-25	3276.6667	1.0076884		20.406768	174.33333	5266	0.96795539	20.406782	43.333333	3859.6667	0.98889743	20.406746	237.33333	2082.3333	0.89768645		20.406737	275.66667	3483.6667	0.9266714	20.406777	108.66667	3872.3333	0.97270368	0.95
20.506739	30.333333	3346.6667	0.99101767		20.506802	328	4565.3333	0.93297003	20.506815	68.333333	3823	0.98243961	20.506779	412.33333	1994.6667	0.82869409		20.50677	541.66667	3379.6667	0.86186671	20.506811	284.33333	3814	0.9306222	0.95
20.606772	-26.333333	3413.6667	1.0077741		20.606835	86.333333	4747.3333	0.98213916	20.606848	-34	3975	1.0086273	20.606812	407.66667	2072.6667	0.83564037		20.606803	632.33333	3679.3333	0.85334364	20.606844	285	3837	0.93085881	0.95
20.706805	28.666667	3474.6667	0.99181732		20.706868	252.33333	4740.6667	0.94946258	20.706882	-124	4188.3333	1.0305093	20.706845	674	2070.6667	0.75443284		20.706836	506.66667	3656.3333	0.8782929	20.706877	164.66667	3503	0.95510315	0.95
20.806838	24	3527.3333	0.99324197		20.806901	170	5533.3333	0.97019287	20.806915	-147.66667	4245	1.0360397	20.806878	651	2408.3333	0.78720854		20.806869	401.66667	3729.6667	0.90277554	20.80691	-98	3516.3333	1.0286689	0.96
20.90687	71	3143.6667	0.97791373		20.906934	443	5151.3333	0.92081273	20.906948	-89.333333	4813.6667	1.0189092	20.906911	518	2462.6667	0.82621338		20.906902	376.66667	3588.6667	0.90501009	20.906944	-62.333333	3659	1.0173309	0.96
21.006903	68.666667	3256.6667	0.97935044		21.006967	309.33333	5238	0.94423747	21.006981	8.6666667	4806	0.99819994	21.006944	575.33333	2561	0.81655861		21.006935	505.66667	3495.6667	0.87362546	21.006977	79	3695.6667	0.979071	0.96
21.106936	204.33333	3051.3333	0.93723764		21.107001	290	4722.6667	0.94214656	21.107015	224	4364.3333	0.95118053	21.106978	482	2137.3333	0.81598371		21.106968	457	3595.6667	0.88723474	21.10701	193.66667	3598.3333	0.94892757	0.96
21.206969	311.33333	3400	0.91611281		21.207034	83.333333	4719.6667	0.98264973	21.207048	92.333333	3763.3333	0.97605256	21.207011	552	2239.6667	0.80226866		21.207001	422.33333	3813	0.90028333	21.207043	350.66667	3401.3333	0.90653873	0.96
21.307002	232	3311	0.93451877		21.307067	122	4582.3333	0.97406646	21.307081	186	3389.6667	0.94798173	21.307044	607.66667	2742.3333	0.81860697		21.307034	258.66667	3699.6667	0.93465263	21.307076	274.33333	3228	0.92167127	0.96

21.407035	315.66667	3058	0.90643217	21.4071	133	4771.3333	0.97288113	21.407114	-4.3333333	3468.3333	1.001251	21.407077	660.66667	2606.6667	0.79779637	21.407067	469.66667	3736.3333	0.88833413	21.40711	265.66667	3248.3333	0.92439765	0.97
21.507068	286.33333	3057.3333	0.91436547	21.507133	359	4756	0.92981427	21.507148	109	3778.6667	0.97196262	21.50711	658.66667	2517	0.79258948	21.5071	467.66667	3522.6667	0.8828001	21.507143	23.333333	3838.3333	0.9939577	0.97
21.607101	374	2955.6667	0.88767644	21.607167	278	4359.6667	0.94005606	21.607181	17	4094.6667	0.99586542	21.607143	614.66667	2222	0.78331375	21.607133	500.33333	3774.3333	0.88295384	21.607176	359.33333	4084.3333	0.91913585	0.97
21.707133	136	3359	0.96108727	21.7072	307	4383.6667	0.93455088	21.707214	-60.333333	4353.3333	1.0140539	21.707176	608.33333	2735.3333	0.818064	21.707166	506.33333	3410	0.8707124	21.707209	321.66667	3950.6667	0.92470937	0.97
21.807166	-32.666667	3210.6667	1.010279	21.807233	149.33333	4605.6667	0.96859446	21.807247	-75.666667	4033.3333	1.019119	21.807209	529	2555.6667	0.82850659	21.807199	507	3443.3333	0.8716564	21.807243	433.66667	3620.6667	0.89303626	0.98
21.907199	-8	3463.6667	1.002315	21.907266	115	4334	0.97415149	21.907281	-127	4030	1.0325391	21.907242	490.66667	2789.3333	0.8504065	21.907232	490	3718	0.88355513	21.907276	180.33333	3348.6667	0.94889959	0.98
22.007232	-102.66667	3294.6667	1.0321637	22.007299	-45.333333	4592.6667	1.0099692	22.007314	80.333333	3748.3333	0.97901793	22.007275	452.66667	2743.3333	0.85836462	22.007266	158.66667	3979.6667	0.96165928	22.007309	311.66667	3500.6667	0.91824779	0.98
22.107265	5.6666667	3208.6667	0.99823706	22.107332	-58.333333	4522.6667	1.0130665	22.107347	178.33333	3993	0.95724788	22.107308	402	2780	0.87366436	22.107299	112.66667	4201.6667	0.9738855	22.107342	283.66667	3207.6667	0.91875119	0.98
22.207298	-64.666667	3007.6667	1.021973	22.207366	99.666667	4440.6667	0.9780486	22.20738	145	4336.6667	0.96764597	22.207341	481.66667	2553.6667	0.84131342	22.207332	235	3937	0.9436721	22.207375	140.66667	3477.3333	0.96112032	0.98
22.307331	64	2933	0.97864531	22.307399	152	4257.6667	0.96553027	22.307414	-36	4374.3333	1.0082981	22.307374	255	2540.6667	0.90878741	22.307365	310.33333	4127.6667	0.93007361	22.307409	109.33333	3638	0.9708237	0.98
22.407364	43	3169.6667	0.98661548	22.407432	269.33333	4134	0.93883422	22.407447	-190.66667	3861	1.0519481	22.407407	391	2566.6667	0.86780119	22.407398	322.66667	4129.3333	0.92752321	22.407442	-33	3794.6667	1.0087727	0.98
22.507396	-28.666667	3490.6667	1.0082804	22.507465	203.33333	4222.3333	0.95405589	22.50748	-171.33333	3837.3333	1.0467358	22.50744	447	2591.3333	0.85287987	22.507431	381.66667	4106.3333	0.91495841	22.507475	123	3547.3333	0.96648806	0.98
22.607429	56.666667	3694.6667	0.98489426	22.607498	167	4288.3333	0.96251683	22.607513	-7.3333333	4444	1.0016529	22.607474	646.33333	2408.6667	0.78843426	22.607464	556.33333	3873.3333	0.8744074	22.607508	187.33333	3103.3333	0.94307131	0.98
22.707462	142.66667	3326.3333	0.95887383	22.707532	99.666667	4646.3333	0.97899986	22.707547	180.66667	4314.3333	0.95980719	22.707507	761.33333	2364.3333	0.7564253	22.707497	667.33333	3668.6667	0.84609471	22.707542	170	2952.3333	0.94555354	0.98
22.807495	166.66667	3375.6667	0.95295003	22.807565	87	5232	0.98364354	22.80758	125	3878.6667	0.96887862	22.80754	741.66667	2689	0.78381267	22.80753	614.66667	3415.3333	0.84747725	22.807575	182	3215.6667	0.94643383	0.99
22.907528	21.666667	3550	0.99393374	22.907598	163.66667	5119.3333	0.96902013	22.907613	2	3647.6667	0.999452	22.907573	651.33333	2910.3333	0.81712681	22.907563	604	3402.3333	0.84923871	22.907608	172	3408	0.95195531	0.99
23.007561	89	3585	0.97577572	23.007631	232.66667	5050.6667	0.95596215	23.007646	-122.33333	4011.3333	1.0314562	23.007606	533.66667	2739	0.83693217	23.007596	598.66667	3065.6667	0.83662331	23.007641	262	3645	0.93294088	0.99
23.107594	302	3491	0.92037965	23.107664	324.33333	5087	0.94006406	23.10768	79	4052.6667	0.98087939	23.107639	196	2271	0.92055128	23.107629	493.66667	2947.3333	0.85653395	23.107674	231	3225.6667	0.93317261	1
23.207627	331.66667	3357.3333	0.91009307	23.207697	484.33333	4952	0.91090809	23.207713	49.333333	4029.3333	0.98790454	23.207672	348.66667	2146.3333	0.86025384	23.207662	398	3099.6667	0.88620985	23.207708	-27	3321.3333	1.0081959	1
23.307659	323	3748.6667	0.92067131	23.307731	558.66667	4456.3333	0.88860086	23.307746	6.3333333	4059.6667	0.99844237	23.307705	232.66667	2315	0.9086746	23.307695	242.33333	3082.3333	0.92711049	23.307741	-26	2980.6667	1.0087996	1
23.407692	244.33333	3589.3333	0.93626641	23.407764	259.66667	4448	0.94484175	23.407779	-141	4133	1.0353206	23.407738	509.33333	2372.6667	0.82327088	23.407728	488.66667	3448.3333	0.87587842	23.407774	-86.666667	2952.6667	1.0302396	1
23.507725	279.33333	3800.3333	0.93153035	23.507797	101.33333	4240.6667	0.97666206	23.507813	-130.33333	4032	1.0334045	23.507771	348.33333	2151	0.8606295	23.507761	412.66667	3293.3333	0.88864904	23.507807	-30.666667	2719	1.0114073	1
23.607758	223.66667	3542	0.9406037	23.60783	-59	5483.3333	1.0108769	23.607846	-72.333333	3592	1.0205512	23.607804	390	2214.3333	0.85024958	23.607794	412.33333	3525	0.89527599	23.607841	-55	2743.3333	1.0204588	1
23.707791	120.66667	3958	0.97041517	23.707863	382	5103.3333	0.93035975	23.707879	-70	3679	1.019396	23.707837	220.66667	2330.3333	0.91349797	23.707827	253	3430	0.931306	23.707874	-106	3321	1.0329705	1
23.807824	122.66667	3525.3333	0.96637427	23.807896	369.66667	5117.6667	0.93263273	23.807912	-144.33333	3554	1.0423306	23.80787	223.33333	2311.3333	0.91188848	23.80786	303.33333	3401.6667	0.91812865	23.807907	-54	3312.3333	1.0165729	1
23.907857	79.333333	3782.6667	0.97945797	23.90793	599.33333	4238	0.87610254	23.907945	-163	4100	1.0414021	23.907903	360	2846	0.88771054	23.907893	514.33333	3464.6667	0.87073804	23.90794	165.66667	3339.3333	0.95273419	1
24.00789	-41	3170.6667	1.0131004	24.007963	286.66667	4271.3333	0.93710692	24.007979	-89	4044.3333	1.0225013	24.007937	592.66667	2815.3333	0.82609546	24.007926	352.66667	3386	0.90567047	24.007973	149.66667	2838.3333	0.94991075	1
24.107922	-7	3483	1.0020138	24.107996	379.33333	4407	0.92074657	24.108012	-122	4239.6667	1.0296284	24.10797	783.66667	2921.3333	0.78848403	24.107959	318.33333	3919.6667	0.92488595	24.108007	35.333333	2908.3333	0.98799683	1
24.207955	41.333333	3582.3333	0.98859351	24.208029	153.66667	4477.6667	0.96682021	24.208045	-15.666667	4146.6667	1.0037925	24.208003	872.66667	2489.6667	0.74045802	24.207992	168.66667	3881	0.95835048	24.20804	-221.33333	3000.3333	1.079645	1
24.307988	149.66667	3637	0.96047535	24.308062	389	4628.6667	0.92247393	24.308078	126.33333	3893	0.96856859	24.308036	778	2610	0.770366	24.308025	478.33333	3842.3333	0.88929178	24.308073	-146.66667	3284.3333	1.0467439	1
24.408021	274	3619	0.92961726	24.408096	82.333333	4757	0.98298664	24.408112	399.66667	3988.6667	0.90892518	24.408069	579.33333	2493.6667	0.8114763	24.408058	505.66667	3484.3333	0.8732665	24.408106	-13.333333	3355	1.00399	1
24.508054	206.33333	3304.6667	0.94123232	24.508129	-28.666667	4615	1.0062505	24.508145	465.66667	4492.3333	0.90607772	24.508102	322	2289.6667	0.87670708	24.508091	436	3551.6667	0.89066288	24.50814	86.333333	3687.3333	0.97712216	1
24.608087	411.66667	3444.3333	0.89323997	24.608162	60	4630.6667	0.98720864	24.608178	178.66667	4745.3333	0.96371514	24.608135	172.66667	2025.3333	0.92144374	24.608124	163.33333	3963.3333	0.96042003	24.608173	-135	3366.3333	1.0417784	1
24.70812	139.33333	3366.6667	0.9602586	24.708195	270	5052	0.94926719	24.708211	1	4782	0.99979093	24.708168	209.33333	2218	0.91375996	24.708157	116.66667	3783.6667	0.97008803	24.708206	-195.33333	3570.3333	1.0578765	1
24.808153	288.33333	3791	0.92931852	24.808228	686.66667	4917	0.87746119	24.808245	-59.333333	4297.3333	1.0140003	24.808201	185.33333	2280.3333	0.92483439	24.80819	157.33333	3589.3333	0.95800712	24.808239	-193.33333	3083	1.0669051	
24.908185	170	3785.6667	0.95702368	24.908261	596.66667	5321.3333	0.89917765	24.908278	-24	4018	1.006009	24.908234	169.33333	2472.6667	0.93590714	24.908223	250.66667	3020.6667	0.92337477	24.908272	-12.666667	3012.3333	1.0042227	
25.008218	133.33333	3555	0.96384998	25.008295	828.66667	4782.3333	0.85231391	25.008311	-65.333333	4093.6667	1.0162185	25.008267	252.66667	2644.3333	0.91278334	25.008256	280.33333	3083.3333	0.91665841	25.008306	-19.666667	2847.6667	1.0069543	
25.108251	201	3376.3333	0.9438129	25.108328	559.66667	4989.3333	0.89914099	25.108344	-200	3712.6667	1.0569368	25.1083	497	2882	0.85291506	25.108289	643.33333	3188.6667	0.83211552	25.108339	-140.33333	3326.3333	1.0440469	
25.208284	96	3266	0.97144557	25.208361	582	4227.3333	0.87898531	25.208378	-89.333333	4264	1.0213989	25.208333	597.66667	3349.3333	0.84857698	25.208322	541.66667	3446.3333	0.86417586	25.208372	-208	4134	1.0529801	
25.308317	254.66667	3443.6667	0.93114015	25.308394	393.66667	4554.3333	0.92043923	25.308411	-7.3333333	3821	1.0019229	25.308366	531.66667	3226.6667	0.85853659	25.308355	569.66667	3664	0.86544367	25.308405	-131.33333	4005.6667	1.0338983	
25.40835	142.33333	3267.6667	0.95826002	25.408427	518	5203.3333	0.90946166	25.408444	35.333333	4043.3333	0.99133704	25.408399	437.33333	3090	0.87601588	25.408388	136.33333	3832	0.96564469	25.408439	121.66667	3662.6667	0.96784991	
25.508383	154.33333	3672.6667	0.9596725	25.508461	465	5796.3333	0.92573467	25.508477	-78	4004.6667	1.0198642	25.508433	863	2431.6667	0.73806151	25.508421	147.66667	3933.6667	0.96381901	25.508472	198	2964.6667	0.9373946	
25.608416	183	3373.3333	0.94854251	25.608494	695	5854	0.89387693	25.608511	123	4164	0.97130861	25.608466	855.66667	2527	0.74704375	25.608454	317	3728.6667	0.92164456	25.608505	255.66667	3120.6667	0.92427683	
25.708448	113.33333	3344	0.96721944	25.708527	599.66667	5614.6667	0.90350266	25.708544	262	4244.3333	0.94185961	25.708499	816.66667	2649.6667	0.76440042	25.708487	528.66667	3533.6667	0.86986133	25.708538	62	3157.6667	0.98074335	
25.808481	256	3020.6667	0.92187182	25.80856	312.33333	4898	0.94005502	25.808577	305	3771	0.92517174	25.808532	470.66667	2879.3333	0.85950249	25.80852	640.33333	3274	0.83641318	25.808571	106.33333	3882.3333	0.97334113	
25.908514	367.33333	3315.6667	0.90026247	25.908593	60.666667	4800.6667	0.98752057	25.90861	133	3442.3333	0.96280067	25.908565	344.66667	2779.6667	0.88968313	25.908554	333.33333	3500.3333	0.91305104	25.908605	119.33333	3694	0.96870629	
26.008547	396	2963.3333	0.88211947	26.008626	316.33333	4273.3333	0.93107706	26.008644	-54.333333	3290.6667	1.0167885	26.008598	655.33333	2476	0.79071748	26.008587	124.66667	3795	0.96819457	26.008638	3.6666667	4090.3333	0.99910438	
26.10858	346.33333	3101.6667	0.8995553	26.10866	480.33333	4646.3333	0.90630689	26.108677	-29.666667	3447	1.0086812	26.108631	713.33333	2363.6667	0.76817246	26.10862	244.66667	3772	0.93908714	26.108671	-56.666667	3986.3333	1.0144202	
26.208613	33.333333	3110.3333	0.98939667	26.208693	429.33333	4583.3333	0.91435031	26.20871	-173.33333	3899	1.0465241	26.208664	836.33333	1957.3333	0.70063238	26.208653	560.33333	3717	0.86899938	26.208704	-133.33333	4164	1.0330797	
26.308646	-124.33333	3814.3333	1.0336947	26.308726	300.66667	4362	0.93551616	26.308743	-200	4392	1.0477099	26.308697	577.33333	1983	0.77450853	26.308686	651.33333	3674	0.8494143	26.308738	181.33333	3900	0.95557008	
26.408679	-163.33333	3790	1.0450368	26.408759	158.66667	4483.3333	0.96581933	26.408777	-171.33333	4325	1.0412487	26.40873	524	2035.6667	0.79528584	26.408719	561.66667	3700.3333	0.86821524	26.408771	144.66667	3515.3333	0.96047359	
26.508711	-35.666667	3701.6667	1.009729	26.508792	312.66667	4337.6667	0.93276468	26.50881	-68	4192.3333	1.0164875	26.508763	633	2182.6667	0.77518646	26.508752	507.66667	3374	0.86921426	26.508804	176.66667	3506.6667	0.9520362	
26.608744	163.66667	3329	0.95313991	26.608825	162	5039	0.96885214	26.608843	-52.333333	3943	1.013451	26.608796	622	2309.3333	0.78780987	26.608785	487.66667	2828	0.85292048	26.608837	48.666667	3519.6667	0.98636151	
26.708777	321	3537	0.91679627	26.708859	684.66667	5002.3333	0.87960846	26.708876	-169.66667	4041.6667	1.0438189	26.708829	724.33333	2237	0.75540297	26.708818	474	2757.3333	0.85331133	26.70887	219.33333	3522.6667	0.94138607	
26.80881	462.66667	3342.6667	0.87841626	26.808892	732	5156.3333	0.87568639	26.80891	-255	4135.3333	1.065716	26.808862	695.33333	2571	0.78712113	26.808851	353	2687.3333	0.88389431	26.808904	172.33333	3169	0.94842378	
26.908843	452.33333	3377.6667	0.8818973	26.908925	629.33333	5046.3333	0.88911728	26.908943	-82.666667	4152.6667	1.0203112	26.908896	587	2749.3333	0.82405835	26.908884	421.66667	2992.3333	0.87648897	26.908937	132	3170	0.96002423	
27.008876	356.66667	3158.3333	0.89853011	27.008958	397.33333	4869.6667	0.92456174	27.008976	53.333333	4410.3333	0.98805168	27.008929	557	2714.6667	0.82975038	27.008917	618	3630.6667	0.8545426	27.00897	-42.666667	3078	1.0140567	
27.108909	416.33333	3467.3333	0.8927989	27.108991	223.66667	4787.3333	0.95536486	27.109009	180.33333	4339.6667	0.96010324	27.108962	710.33333	2619.3333	0.78666533	27.10895	814	3516	0.81200924	27.109003	313.33333	3193	0.91063789	
27.208941	525.66667	3216.3333	0.85952254	27.209025	363	4910.6667	0.93116744	27.209043	182.66667	4072.6667	0.95707348	27.208995	666.33333	2342.6667	0.77855323	27.208983	922.66667	3922	0.80955002	27.209037	301.33333	3497	0.92066696	
27.308974	390.33333	3150.6667	0.88976749	27.309058	506	5222.6667	0.91167229	27.309076	205.33333	3907	0.9500689	27.309028	559.66667	2430.3333	0.81282051	27.309016	678.33333	3752	0.84688887	27.30907	284	3568.6667	0.92628482	
27.409007	65.333333	3137.3333	0.97960033	27.409091	634.33333	5315	0.89337741	27.409109	261.33333	3966.3333	0.93818497	27.409061	328	2300.6667	0.87522191	27.409049	733.33333	3620.3333	0.8315596	27.409103	-156.66667	3908.6667	1.0417555	
27.50904	-44.333333	3506	1.0128069	27.509124	696.33333	5314.3333	0.8841504	27.509142	141.33333	4126.6667	0.96688535	27.509094	251	2324.6667	0.9025495	27.509082	842.33333	3084.6667	0.78550208	27.509136	-132.33333	3372.6667	1.0408394	
27.609073	3	3772.3333	0.99920537	27.609157	329	4823.3333	0.93614544	27.609176	24.333333	4034	0.99400411	27.609127	201.66667	2235.3333	0.91724798	27.609115	967	2719.3333	0.73767972	27.609169	122.66667	3764.3333	0.96844181	
27.709106	19.333333	3890.6667	0.99505541	27.70919	48	4465.6667	0.98936563	27.709209	-12.333333	3742.6667	1.0337906	27.70916	211.66667	2488	0.92159526	27.709148	846.66667	3212.6667	0.79142716	27.709203	363	3825.3333	0.91333068	
27.809139	-32.333333	4072.3333	1.0080033	27.809224	-40.666667	3952	1.0103971	27.809242	-3.6666667	3746	1.0009798	27.809193	331.66667	2346.6667	0.87616677	27.809181	571.33333	3793	0.86909035	27.809236	384.33333	4105.3333	0.91439602	
27.909172	101.33333	3837.6667	0.97427435	27.909257	122.66667	4359.3333	0.97263127	27.909275	-9	3864	1.0023346	27.909226	341	2630	0.88522383	27.909214	693.33333	4043.6667	0.85363451	27.909269	138.66667	3478.6667	0.96166605	
28.009204	252	3518	0.9331565	28.00929	344	4878.3333	0.93412906	28.009309	30.666667	4431.6667	0.99312766	28.009259	351.66667	2453.3333	0.87462864	28.009247	545.66667	4604.3333	0.89404531	28.009302	109	2997	0.96490663	
28.109237	330	3504.3333	0.9139355	28.109323	441.66667	5016.3333	0.91907903	28.109342	80.666667	5175	0.98465149	28.109292	310.33333	2715.6667	0.89744437	28.10928	613	4499	0.88008607	28.109336	-3	2827.6667	1.0010621	
28.20927	90.666667	3390.3333	0.97395384	28.209356	850.66667	5300.3333	0.8617027	28.209375	64.333333	5454.3333	0.98834259	28.209325	237.33333	2750.6667	0.92057117	28.209313	479.33333	4464.6667	0.90304746	28.209369	59.333333	2693.6667	0.97844775	
28.309303	158	3283.6667	0.95409201	28.30939	1148.6667	5079	0.81555425	28.309408	-34	5059.3333	1.0067657	28.309358	274.66667	2949.6667	0.91481443	28.309346	467.33333	3729.6667	0.88865062	28.309402	-40	2914.3333	1.0139163	
28.409336	103	2938.3333	0.96613327	28.409423	1228	5274.6667	0.8111544	28.409441	-361.66667	4635.3333	1.0846268	28.409392	369	2794.3333	0.8833509	28.409379	440	3462.3333	0.88724695	28.409435	-50.333333	2862.6667	1.0178974	
28.509369	227.33333	2763.3333	0.92398573	28.509456	693.33333	5359.6667	0.88545625	28.509475	-276.66667	4243.6667	1.069742	28.509425	437.33333	2429	0.84742412	28.509412	238.66667	3456	0.93540238	28.509468	147	3327.6667	0.95769378	
28.609402	65.333333	3073.6667	0.97918658	28.609489	402.66667	4620	0.9198301	28.609508	-238	4243	1.0594257	28.609458	521.33333	2109.6667	0.80184974	28.609445	417	3662.6667	0.89778577	28.609502	260.33333	3114.3333	0.92285658	
28.709435	53	3075	0.98305627	28.709522	396.66667	4827.3333	0.9240684	28.709541	107.66667	4030.6667	0.97398308	28.709491	409.66667	1809.6667	0.81541003	28.709478	305.33333	3787.3333	0.92539502	28.709535	500.33333	3051.6667	0.85914039	
28.809467	57.666667	2990.3333	0.98108049	28.809555	424	4816.3333	0.91908912	28.809574	-52	4417.6667	1.0119111	28.809524	576.33333	1960.6667	0.7728288	28.809511	417	3838.3333	0.90200533	28.809568	338.33333	3295.3333	0.90688928	
28.9095	343	3130.6667	0.90125708	28.909589	560	5723	0.9108706	28.909608	-40.333333	4394.6667	1.0092628	28.909557	553	1884.6667	0.77314372	28.909544	433.66667	3613	0.89283361	28.909601	277.33333	3378	0.92412913	
29.009533	517.66667	3375	0.8670149	29.009622	367.66667	5444.6667	0.93674371	29.009641	-119.33333	4633.6667	1.0264343	29.00959	780.33333	2579	0.76771185	29.009577	411.33333	3560.3333	0.89643307	29.009635	102	3523	0.97186207	
29.109566	703	3908	0.84753849	29.109655	388.33333	5373.6667	0.93260442	29.109674	45.666667	4902.6667	0.9907713	29.109623	815.66667	2644.6667	0.7642809	29.10961	596	3975	0.86961278	29.109668	134	3087	0.95839801	
29.209599	411.33333	3797.3333	0.90226517	29.209688	320.33333	5047.6667	0.94032538	29.209707	83	5125.3333	0.984064	29.209656	740.33333	3161	0.81023582	29.209643	433.66667	4179.3333	0.90599032	29.209701	66.333333	3188.6667	0.9796211	
29.309632	149	3524.3333	0.95943739	29.309721	242.33333	4879.6667	0.95268775	29.30974	-87	4909.3333	1.0180411	29.309689	668	2994.3333	0.81760262	29.309676	580.66667	4159	0.87748787	29.309734	100.33333	3074	0.96839231	
29.409665	100.33333	3032.3333	0.96797191	29.409754	269.66667	4843.6667	0.94726206	29.409774	-51.333333	4550.6667	1.0114091	29.409722	427	3019	0.87608822	29.409709	495.33333	3587.6667	0.87868397	29.409767	-95.666667	2740.3333	1.0361734	
29.509698	93.333333	2811.3333	0.9678678	29.509788	360	4937.6667	0.93204555	29.509807	-82	4437.3333	1.0188275	29.509755	285	2364	0.89241223	29.509742	657.66667	3564.3333	0.84422864	29.509801	-35.333333	2808.6667	1.0127404	
29.60973	72	2837	0.97524923	29.609821	567.66667	5238.6667	0.90223319	29.60984	103.66667	4253.3333	0.97620687	29.609788	256.66667	2410.3333	0.90376203	29.609775	544	3168	0.85344828	29.609834	-139.33333	2805	1.0522696	
29.709763	27.333333	3146	0.99138655	29.709854	803.66667	4827.3333	0.85727816	29.709874	-70	4298.6667	1.0165537	29.709821	339.33333	2623.3333	0.88546355	29.709808	599	3275.6667	0.84540606	29.709867	193	3161	0.94245677	
29.809796	35.333333	3131.6667	0.98884328	29.809887	615.33333	4456	0.87866439	29.809907	142.33333	4710.3333	0.97066905	29.809854	442	2882	0.86702768	29.809841	437.66667	3271.6667	0.88200935	29.8099	361.33333	3265.3333	0.90036765	
29.909829	82.666667	3200.6667	0.97482234	29.90992	374	4693	0.92618907	29.90994	129.33333	4900.3333	0.9742859	29.909888	476	2658.6667	0.84814972	29.909875	674	3588	0.84185828	29.909934	439.66667	3325.6667	0.883233	
30.009862	60.666667	3301.6667	0.98195697	30.009954	66.666667	4809.3333	0.98632759	30.009973	188.33333	4961	0.96342569	30.009921	527.66667	2461	0.82344412	30.009908	639.66667	3505.6667	0.84568993	30.009967	334	3248	0.906756	
30.109895	-95.333333	3663	1.0267215	30.109987	-109	5505.3333	1.0201989	30.110007	-13.333333	4583	1.0029178	30.109954	437.33333	2415.3333	0.84669315	30.109941	684.66667	3387.3333	0.83185986	30.11	136.33333	3123.6667	0.95817996	
30.209928	47	3600.3333	0.98711387	30.21002	-51.333333	4908.3333	1.0105689	30.21004	86	4418.6667	0.98090869	30.209987	480	2588	0.84354628	30.209974	478.33333	3249.3333	0.87168023	30.210033	178.66667	3354.3333	0.94942919	
30.309961	-3.3333333	3590.3333	1.0009293	30.310053	132.33333	4736	0.97281753	30.310073	88.333333	4478.6667	0.98065835	30.31002	537	2486	0.82236189	30.310007	296	2887.6667	0.90702544	30.310066	212	3506	0.9429801	
30.409993	185.33333	3216.3333	0.9455169	30.410086	394.33333	4776	0.92373155	30.410106	73.333333	5001.3333	0.98554913	30.410053	522.66667	2584	0.83175966	30.41004	233	3341.6667	0.9348191	30.4101	358.66667	3639.3333	0.91028848	
30.510026	267	3311.3333	0.92538426	30.510119	684	4898.3333	0.87747059	30.51014	-235	5455	1.0450192	30.510086	703.33333	1795.6667	0.71855409	30.510073	284.33333	3325.3333	0.92123003	30.510133	300	3905.3333	0.92866202	
30.610059	209	3416.3333	0.94235013	30.610153	651.33333	4643	0.87697538	30.610173	-127.66667	4937	1.0265456	30.610119	1029	928	0.4741952	30.610106	352	3936.3333	0.91791683	30.610166	455	4111	0.90035042	
30.710092	205	3811.3333	0.94895842	30.710186	649	4906.3333	0.88317533	30.710206	-188	4761	1.0411109	30.710152	1479.6667	128	0.079618495	30.710139	539	3426	0.86406053	30.710199	440.66667	3912.6667	0.89877489	
30.810125	-0.33333333	4062	1.0000821	30.810219	376.66667	4976	0.92963009	30.810239	-42	4508.3333	1.0094037	30.810185	1539.6667	34.666667	0.022019903	30.810172	833.66667	3423	0.80415035	30.810233	477.33333	3185.6667	0.86968787	
30.910158	-37.333333	3941	1.0095637	30.910252	468.33333	5369.6667	0.91977846	30.910273	-193.33333	4659.6667	1.0432868	30.910218	1610.6667	20.666667	0.012668574	30.910205	785.33333	3381	0.81150492	30.910266	211.66667	3065.3333	0.9354084	
31.010191	75	3767	0.98047892	31.010285	263	5339	0.95305248	31.010306	-38.666667	4800	1.008121	31.010251	1463	79.333333	0.051437216	31.010238	725.33333	3389	0.82370574	31.010299	80	3239	0.97589635	
31.110224	536.33333	3360.3333	0.86236099	31.110319	477	4898.6667	0.91126682	31.110339	10.333333	5084.6667	0.99797187	31.110284	1412.6667	-42	-0.030642023	31.110271	404.33333	3305.6667	0.89101527	31.110332	-15.666667	4015.3333	1.003917	
31.210256	533	3005	0.84934992	31.210352	382	4770	0.92585404	31.210372	-26	5221	1.0050048	31.210317	1120	38.333333	0.033093525	31.210304	254	2742.6667	0.91523915	31.210365	-119.33333	3721.6667	1.0331267	
31.310289	406.33333	2764.6667	0.87185956	31.310385	499	4534.6667	0.90086749	31.310406	-179	4730.3333	1.0393291	31.310351	1188.6667	-141.33333	-0.13494589	31.310337	194.66667	2900	0.93709608	31.310399	-112	3992.6667	1.028861	
31.410322	15.333333	2821	0.99459396	31.410418	289.66667	4843.3333	0.94356776	31.410439	-161.66667	4577.3333	1.0366121	31.410384	1229.3333	-139.66667	-0.12817035	31.41037	223.66667	3203.3333	0.93473398	31.410432	-155.66667	3431.6667	1.0475173	
31.510355	-2	2709.3333	1.0007387	31.510451	300.66667	4798	0.94103033	31.510472	-73	4936	1.0150113	31.510417	1754.6667	-104	-0.063004847	31.510403	519	3554.3333	0.87258592	31.510465	-174	3771.3333	1.0483692	
31.610388	33.666667	2858.6667	0.98836003	31.610484	370	4988.3333	0.93094868	31.610505	-56.666667	5386	1.010633	31.61045	1693.6667	-8	-0.004745897	31.610436	527.66667	3373	0.86472398	31.610498	-12	3491.3333	1.0034489	
31.710421	-49.666667	3399.6667	1.0148259	31.710518	487.33333	5290.3333	0.91565222	31.710539	-68	5459.6667	1.0126121	31.710483	1718.6667	94	0.0518573	31.710469	578.33333	3062.6667	0.84116085	31.710532	324	3867.6667	0.92270378	
31.810454	42.666667	3526.3333	0.9880452	31.810551	361.33333	5557.6667	0.93895365	31.810572	2.3333333	5063.3333	0.99953938	31.810516	1761	173	0.089451913	31.810502	519.33333	3127.6667	0.85759985	31.810565	329.33333	3777	0.91979868	
31.910487	49.666667	3111.6667	0.98428933	31.910584	473	5868.3333	0.92541001	31.910605	235	4694	0.95232299	31.910549	2091	222.33333	0.09610951	31.910535	520.33333	3570.3333	0.87279987	31.910598	225.33333	3854.3333	0.94476673	
32.010519	-3.6666667	2819	1.0013024	32.010617	273	5516	0.9528416	32.010638	251.66667	5075.3333	0.9527564	32.010582	2350.3333	279.33333	0.10622386	32.010568	535.33333	3781.3333	0.87598456	32.010631	15	3892.3333	0.99616106	
32.110552	-43.333333	3023.6667	1.0145398	32.11065	246	4780	0.95105452	32.110672	131.66667	5307	0.97579063	32.110615	2036.6667	161.33333	0.073400061	32.110601	533	3920.6667	0.88032333	32.110664	112.66667	3783.6667	0.97108393	

32.210585	-156.33333	3261.6667	1.0503435
32.310618	70.666667	3352.6667	0.97935735
32.410651	185.33333	3657.3333	0.9517696
32.510684	212.33333	3671.3333	0.94532658
32.610717	293.66667	3539	0.92337798
32.71075	206	3358	0.94219978
32.810782	489	3359.6667	0.87294301
32.910815	302	2982.3333	0.90804831
33.010848	283.33333	2373.6667	0.89336344
33.110881	193.66667	2417	0.92581716
33.210914	331.33333	2611.6667	0.88741647
33.310947	465.66667	3125	0.87031192
33.41098	499.66667	3289	0.86811543
33.511012	418.66667	3138.3333	0.88229782
33.611045	259.33333	3456.6667	0.9302117
33.711078	172.33333	3550.3333	0.95370702
33.811111	121.66667	3656	0.96779317
33.911144	222.66667	2805	0.92645602
34.011177	129.66667	2633.6667	0.953076
34.11121	33	2791.6667	0.98831721
34.211243	-111	3760.3333	1.0304165
34.311275	-102.66667	3716	1.0284133
34.411308	52	3449	0.9851471
34.511341	224.66667	2957.3333	0.92939451
34.611374	43.333333	3222	0.98672928
34.711407	-29.333333	3456.6667	1.0085586
34.81144	2	3464.6667	0.99942308
34.911473	216.66667	3616.3333	0.94347335
35.011506	251	3600	0.93482212
35.111538	42.666667	3779.3333	0.98883656
35.211571	116.66667	3976.3333	0.97149605
35.311604	37	3772	0.99028616
35.411637	307.33333	3448.3333	0.9181681
35.51167	334.33333	2765.3333	0.89213894
35.611703	396.33333	3061.3333	0.88537549
35.711736	154	2937	0.95017794
35.811769	13.666667	3020.3333	0.9954955
35.911801	24.666667	2891	0.99153996
36.011834	113.66667	3253.3333	0.96624097
36.111867	-63.666667	3650.3333	1.0177509
36.2119	-157	3972.3333	1.0411497
36.311933	-18	4193	1.0043114
36.411966	169.33333	3939.6667	0.95878965
36.511999	394.33333	3540.6667	0.89978823
36.612032	293	3448	0.9216787
36.712064	495	3979.6667	0.88937723
36.812097	373	4049.3333	0.91565539
36.91213	477.33333	3527	0.8807958
37.012163	313.66667	3165	0.90983135
37.112196	253	3277.6667	0.92834215
37.212229	57	3630.6667	0.98454307
37.312262	-4	3111.6667	1.0012871
37.412295	102	3046.6667	0.96760534
37.512327	47	3101	0.98506989
37.61236	249	3805.6667	0.93858928
37.712393	119.33333	3572.3333	0.96767494
37.812426	463	3373.6667	0.87932233
37.912459	450	1846.6667	0.80406386
38.012492	878.33333	889	0.50301773
38.112525	1051.3333	-79	-0.081247857
38.212558	1266.6667	-87	-0.073749647
38.31259	1361.6667	29.666667	0.021322472
38.412623	1248.6667	-31.666667	-0.026020268
38.512656	1512	70.333333	0.044449126
38.612689	1220.6667	53.333333	0.041862899
38.712722	1196.3333	139	0.10409386
38.812755	1043.6667	-20	-0.01953761
38.912788	1231.6667	11.666667	0.009383378
39.012821	1314.3333	11	0.008299799
39.112853	1338.3333	78.666667	0.055516349
39.212886	1423	57.333333	0.038730016
39.312919	1288.3333	146	0.10178945
39.412952	1155.3333	66	0.054039301
39.512985	1085.6667	21	0.018975904
39.613018	1203.6667	99.666667	0.076470588
39.713051	1089.3333	169.33333	0.1345339
39.813083	1103.6667	254.33333	0.18728522
39.913116	919.66667	150.33333	0.14049844
40.013149	1142.6667	97	0.078246841
40.113182	1210.3333	-21.666667	-0.018227706
40.213215	1295.6667	-190	-0.17184203
40.313248	1267.3333	-211.33333	-0.20012626
40.413281	1209.6667	-157.33333	-0.14950903
40.513314	1270.3333	-124	-0.10817098
40.613346	1343.6667	41.666667	0.030076997
40.713379	1298	41	0.030619866
40.813412	1303.3333	198.66667	0.13226809
40.913445	1166.6667	36	0.029933481
41.013478	1041	100	0.087642419
41.113511	1105.6667	-31	-0.028846154
41.213544	1139.6667	-108.66667	-0.10539929
41.313577	1297.3333	-206.66667	-0.18948655
41.413609	1367.3333	-207	-0.17839701
41.513642	1449	-69.666667	-0.050507492
41.613675	1707	-44.333333	-0.026663994
41.713708	1497.6667	23	0.015124945
41.813741	1576	-1.3333333	-0.00084674
41.913774	1153	-74.333333	-0.068912237
42.013807	1237.6667	-262.66667	-0.26940171

32.210683	128.33333	4217.6667	0.97047093
32.310717	216.66667	4163.6667	0.95053649
32.41075	282	5090.3333	0.94750884
32.510783	145	5600.6667	0.97476359
32.610816	161.66667	5551.6667	0.97170362
32.710849	82	5497	0.98530203
32.810883	-49	4976.6667	1.0099439
32.910916	56.666667	4968.3333	0.98872305
33.010949	52	4288.3333	0.98801935
33.110982	274	3978.6667	0.93556984
33.211015	288.33333	4502.3333	0.93981353
33.311048	288.33333	5205	0.94751214
33.411082	205.66667	5762.6667	0.96554035
33.511115	346.33333	5459.6667	0.94034906
33.611148	357.33333	5173	0.93538666
33.711181	407.33333	4977.3333	0.9243531
33.811214	292.33333	5101.6667	0.94580398
33.911248	219.33333	6107	0.9653301
34.011281	4.6666667	5893	0.99920873
34.111314	-160	5983.6667	1.0274741
34.211347	127.66667	5353.6667	0.97670883
34.31138	495	5305.3333	0.91466008
34.411413	388	5138.3333	0.9297907
34.511447	154.33333	4959.6667	0.96982141
34.61148	-3	5528	1.000543
34.711513	215.33333	5988.3333	0.96528935
34.811546	510	6233.3333	0.92436975
34.911579	608.33333	5817	0.90532268
35.011612	575.33333	5525.6667	0.90569852
35.111646	555	5491.3333	0.90820883
35.211679	594.33333	5208.6667	0.89758171
35.311712	337.33333	5083.6667	0.93777286
35.411745	361	4537.6667	0.92630648
35.511778	287.33333	4879.3333	0.9443871
35.611812	624	5616	0.9
35.711845	490.33333	5533.3333	0.91859886
35.811878	573.33333	5342.3333	0.90308221
35.911911	443.33333	4781.6667	0.91515152
36.011944	437.66667	5213.6667	0.92255515
36.111977	367	5492.6667	0.93736845
36.212011	303.33333	5101	0.9438722
36.312044	169.33333	4903	0.96661628
36.412077	237.66667	4806.6667	0.95288442
36.51211	348	5405	0.93950982
36.612143	478.33333	5890	0.92488877
36.712177	186.66667	5987.3333	0.96976568
36.81221	112.33333	5927.6667	0.98140177
36.912243	64.333333	5432.6667	0.98829665
37.012276	624.33333	5106.6667	0.89106032
37.112309	822.66667	4279	0.83874551
37.212342	1048.6667	3959.6667	0.79061564
37.312376	640	3931	0.85998687
37.412409	564.33333	3956.6667	0.87517511
37.512442	519.66667	3734.3333	0.87784046
37.612475	849.66667	3435.6667	0.80172682
37.712508	831.33333	3804	0.82065296
37.812541	826.33333	4559.6667	0.84657755
37.912575	753.33333	4644	0.8604249
38.012608	705.33333	4913.6667	0.87447351
38.112641	620.33333	4640	0.88207338
38.212674	292.33333	4928	0.94400102
38.312707	382.33333	4788.3333	0.92605725
38.412741	524.33333	4661	0.89888146
38.512774	521.66667	5580.6667	0.91451357
38.612807	398.33333	5389	0.93117152
38.71284	9.3333333	5841.6667	0.99840483
38.812873	-24.333333	5305	1.004608
38.912906	-229.33333	5693.3333	1.0419717
39.01294	-233	4971.3333	1.0491734
39.112973	-172.66667	4728	1.0379043
39.213006	18.666667	4575	0.99593643
39.313039	272.66667	4959	0.94788149
39.413072	247.33333	4616.3333	0.94914673
39.513106	634	4397.6667	0.87399801
39.613139	515.33333	4358	0.89425445
39.713172	484.33333	4678.6667	0.90619149
39.813205	147.33333	5013	0.97144887
39.913238	134	5287.3333	0.97528283
40.013271	178.66667	5484.6667	0.96845203
40.113305	38	5655.3333	0.99332553
40.213338	181.66667	5617.6667	0.96867456
40.313371	317	5330.6667	0.94387063
40.413404	598	5150.6667	0.89597588
40.513437	508	4553.6667	0.8996378
40.61347	375	4927	0.92927197
40.713504	-12	5005	1.0024034
40.813537	1	5436	0.99981608
40.91357	93.333333	5425	0.98308668
41.013603	196	5191.6667	0.96362061
41.113636	160	5310	0.97074954
41.21367	172	5100.6667	0.96737894
41.313703	466.66667	6103.6667	0.92897367
41.413736	554.33333	6210.3333	0.9180546
41.513769	584.66667	6251.3333	0.9144724
41.613802	186	5787	0.96885987
41.713835	21.333333	5534.3333	0.99616008
41.813869	-91.666667	5286.6667	1.0176452
41.913902	481.66667	4449.3333	0.90231866
42.013935	713.33333	4460.6667	0.86213117
42.113968	985.66667	4433.6667	0.81812031
42.214001	634.66667	4819.3333	0.88363281
42.314035	639	4906.6667	0.8847749
42.414068	396	4685.6667	0.92207281
42.514101	259	4577.6667	0.94645072
42.614134	36.333333	4356	0.99172801
42.714167	91	4318.6667	0.97936352
42.8142	136	3897.6667	0.96628378
42.914234	252.33333	4409	0.9458667

32.210705	63.666667	5304.3333	0.98813959
32.310738	65.666667	4756.6667	0.9863828
32.410771	79	4932	0.98423468
32.510805	41.666667	5076.3333	0.9918588
32.610838	-67.666667	5244.6667	1.0130706
32.710871	62.333333	4694	0.98689467
32.810904	-61.666667	5089	1.0122663
32.910938	31.333333	5097	0.99389015
33.010971	-37.333333	5236	1.0071813
33.111004	175	4569.3333	0.96311389
33.211037	209.33333	4618.6667	0.95664181
33.31107	131	4684	0.97279335
33.411104	-33.666667	5240.3333	1.0064661
33.511137	-55.666667	5205.3333	1.0108098
33.61117	100	5108.6667	0.98080123
33.711203	87	4389	0.980563
33.811237	31	4114.6667	0.99252231
33.91127	-119.66667	3996.6667	1.0308658
34.011303	-152.66667	4316.6667	1.0366635
34.111336	-54.666667	4418.3333	1.0125277
34.21137	-93	4536.3333	1.0209302
34.311403	52.666667	4599.6667	0.98867952
34.411436	-148.33333	4809	1.0318266
34.511469	53.333333	5363.3333	0.99015385
34.611503	73.666667	5158.6667	0.98592088
34.711536	57	4871.6667	0.98843501
34.811569	-96.333333	4431	1.0222239
34.911602	-77.333333	4330.6667	1.0181818
35.011636	-17	4818	1.0035409
35.111669	-4.6666667	4768	1.0009797
35.211702	-320.66667	5022.6667	1.0681979
35.311735	-199.33333	5180.6667	1.0400161
35.411769	-251	5579.6667	1.0471037
35.511802	-24.666667	6201	1.0039937
35.611835	-238	5796	1.0428212
35.711868	-94.333333	5594.3333	1.0171515
35.811902	-49	5404.6667	1.0091492
35.911935	99.666667	5174.3333	0.98110226
36.011968	79.333333	5204.6667	0.98498612
36.112001	18	4694.6667	0.99618051
36.212035	226.66667	4549.6667	0.95254379
36.312068	201	4438	0.9566717
36.412101	215.66667	4792.6667	0.95693844
36.512134	-133.66667	4592.3333	1.0299791
36.612168	-222.33333	4466.6667	1.0523836
36.712201	-161.33333	3841.6667	1.0438366
36.812234	-62.666667	4011	1.0158717
36.912267	-25	3918.6667	1.0064207
37.012301	-8.3333333	3872	1.0021568
37.112334	-39.333333	3862.6667	1.0102877
37.212367	18.666667	4014.6667	0.9953719
37.3124	-75.333333	4652.6667	1.0164579
37.412434	165.66667	4680.3333	0.96581373
37.512467	353.33333	4122	0.92104871
37.6125	215	3775	0.94611529
37.712533	-120.33333	3892.3333	1.0319017
37.812566	-430	4337.3333	1.1100495
37.9126	-246	4106.3333	1.0637251
38.012633	-63	4065.3333	1.0157408
38.112666	109	4282.6667	0.97518027
38.212699	101.33333	4422.3333	0.97759929
38.312733	-88.333333	4558.6667	1.0197599
38.412766	-225.66667	4243.3333	1.0561686
38.512799	-357.33333	3979.6667	1.0986473
38.612832	-312.66667	3634.6667	1.09412
38.712866	-71.666667	3774.3333	1.0193554
38.812899	152	4034	0.96368849
38.912932	205.33333	4278.6667	0.95420755
39.012965	37	4068.3333	0.99098733
39.112999	52.333333	3923	0.98683549
39.213032	-13	3722.3333	1.0035047
39.313065	0.66666667	3951	0.99983129
39.413098	-9.3333333	3995.6667	1.0023413
39.513132	274.33333	4342.3333	0.94057762
39.613165	248.33333	4529.6667	0.94802567
39.713198	104.66667	4722.6667	0.97831791
39.813231	35.333333	4871	0.99279842
39.913265	98.333333	4775	0.97982216
40.013298	411.66667	4751	0.92026085
40.113331	305.33333	4883.3333	0.9411538
40.213364	420	4626	0.91676576
40.313398	132	4416	0.97097625
40.413431	100.33333	4195.6667	0.97664494
40.513464	16	4334.3333	0.99632212
40.613497	-16.666667	4841.3333	1.0034545
40.713531	-10	5024.6667	1.0019942
40.813564	-2.3333333	4773.6667	1.000489
40.913597	-61.333333	4063.3333	1.0153257
41.01363	81.333333	3660	0.97826087
41.113664	3.3333333	3566.6667	0.99906629
41.213697	40.666667	3967.3333	0.98985363
41.31373	85	4296.3333	0.98059951
41.413763	113.66667	4709	0.97643074
41.513797	127.33333	4446.6667	0.97216149
41.61383	-13	3997.3333	1.0032628
41.713863	36.666667	3736	0.99028097
41.813896	48	4056.6667	0.98830599
41.91393	48	3975	0.98806861
42.013963	-44.333333	3946.3333	1.0113617
42.113996	-146.33333	3977.6667	1.0381938
42.214029	-236.33333	3988.3333	1.0629886
42.314063	-247.66667	3805.3333	1.0696149
42.414096	-117	4024.3333	1.0299437
42.514129	62.333333	3571.6667	0.98284718
42.614162	133.33333	3837	0.9664176
42.714195	148.33333	3450	0.95877721
42.814229	4.6666667	4447.3333	0.99895178
42.914262	-114.66667	4458.6667	1.0263966

32.210648	1925.3333	121.33333	0.059283388
32.310681	1800.6667	-25.666667	-0.014460094
32.410714	2016.6667	-72	-0.03702434
32.510747	2015	-125.66667	-0.066513761
32.61078	1936.6667	-3.3333333	-0.001724138
32.710813	1656.3333	24.333333	0.014478382
32.810847	1787.3333	157.66667	0.081062554
32.91088	1727.3333	18	0.010313216
33.010913	1711	-29	-0.017241379
33.110946	1616	-175.66667	-0.12196251
33.210979	1519	-121	-0.086552217
33.311012	1650	-67.333333	-0.042544229
33.411045	1618.3333	-109.66667	-0.072691118
33.511078	1933.3333	-131.33333	-0.072881983
33.611111	1854.6667	-75.333333	-0.042337954
33.711144	2010.6667	-72.666667	-0.0374957
33.811177	1836.6667	-73.333333	-0.041587902
33.91121	1849	-218.33333	-0.13389207
34.011243	1921.3333	-95.666667	-0.052400949
34.111276	2016	-28	-0.014084507
34.21131	2063.6667	-54.666667	-0.027210884
34.311343	1786.3333	-99	-0.058672461
34.411376	1693	-167	-0.10943644
34.511409	1859.6667	-44	-0.024233523
34.611442	2089.3333	-52	-0.02552356
34.711475	2134.3333	-23.333333	-0.011053213
34.811508	2037	71	0.033681214
34.911541	1989	123	0.058238636
35.011574	1967.3333	135.33333	0.064362714

32.210634	427	3345.6667	0.88681746
32.310667	307	3514	0.91965454
32.4107	381.33333	3112	0.89083969
32.510733	422.66667	3297.3333	0.88637993
32.610766	402.66667	3313	0.89163003
32.710799	189	3642	0.95066562
32.810832	184.66667	3633	0.95162839
32.910865	160.66667	3728	0.95868335
33.010898	150.33333	3430.6667	0.95801918
33.110931	189	3542.6667	0.94935239
33.210964	199	3153	0.94063246
33.310997	187	3093	0.9429878
33.41103	148	3076.3333	0.95409904
33.511063	106.33333	3107.3333	0.96691215
33.611096	229.66667	3432.3333	0.93728382
33.711129	367.66667	3231.6667	0.89785145
33.811162	784	3471.6667	0.81577505
33.911196	741.33333	3462.6667	0.82366001
34.011229	692	3828	0.84690265
34.111262	367.66667	3469.3333	0.90417861
34.211295	270.66667	3498	0.92817973
34.311328	487.66667	3484	0.8772136
34.411361	421.33333	3677	0.89719398
34.511394	537.33333	3453.3333	0.86535249
34.611427	579	3521	0.85878049
34.71146	702.66667	3427.3333	0.82986279
34.811493	718.33333	3335.3333	0.82279418
34.911526	422	2689.6667	0.86438136
35.011559	805	2054.6667	0.71849866
35.111592	2226	1014	0.31296296
35.211625	3033.3333	289.66667	0.087170228
35.311658	3664.6667	-145.66667	-0.041394336
35.411691	3116.6667	135	0.041517171
35.511724	3284	195.33333	0.056141023
35.611757	2997	252	0.077562327
35.71179	2833.6667	88.666667	0.030341052
35.811823	2397.3333	31	0.012765957
35.911856	2581.3333	32	0.012244898
36.011889	2866	-67.666667	-0.02418106
36.111922	3432.3333	-186	-0.05729541
36.211955	3363.6667	-215.66667	-0.068509106
36.311988	3227	-226	-0.075308231
36.412021	2900	-213	-0.079270562
36.512054	2794.6667	-21	-0.007571205
36.612087	2929	52.666667	0.017663499
36.71212	3077.3333	145.33333	0.045097228
36.812153	3088.3333	-64	-0.021161689
36.912186	3161.6667	-47.333333	-0.015198544
37.012219	3068	166.33333	0.051427394
37.112252	3218.3333	202.33333	0.059150263
37.212285	2959	144.66667	0.046611535
37.312318	3033.6667	-19	-0.006302521
37.412351	2967	-57	-0.019587629
37.512384	3284.6667	14	0.004244139
37.612417	3332.6667	-77	-0.02365107
37.71245	3266.6667	-22.666667	-0.006987259
37.812483	2885	-53	-0.018714689
37.912517	2732.6667	-24.333333	-0.008984615
38.01255	2722	-23	-0.008521675
38.112583	3113.6667	-132.33333	-0.044387299
38.212616	3181.6667	-58.333333	-0.018676628
38.312649	3250.6667	-149.33333	-0.048151333
38.412682	3005.3333	-45	-0.015200991
38.512715	3025	-68	-0.02299628
38.612748	2843.6667	24.666667	0.008599651
38.712781	2731	156	0.054035331
38.812814	2815	200	0.066334992
38.912847	2875.6667	148.66667	0.049156839
39.01288	2792	61	0.021381002
39.112913	2714.3333	-0.66666667	-0.00024567
39.212946	2827	-29.666667	-0.010605338
39.312979	2979.6667	-130	-0.045619371
39.413012	3084	-213	-0.074190178
39.513045	3128.6667	-70.666667	-0.023108786
39.613078	2951	-60.333333	-0.020871771
39.713111	3103	231.33333	0.069379186
39.813144	3216.3333	199.33333	0.058358544
39.913177	3907.3333	310.33333	0.073579388
40.01321	3488	94.666667	0.026423521

32.210698	74.333333	3760.3333	0.98061544
32.310731	207	3738	0.94752852
32.410764	361.33333	3688.6667	0.91078189
32.510797	255	3851	0.93789576
32.610831	342.33333	3250.6667	0.90472214
32.710864	182.66667	3478.3333	0.95010471
32.810897	252	3035.3333	0.92334212
32.91093	91.666667	3398.6667	0.97373699
33.010963	176.33333	3312	0.94945055
33.110997	379.33333	3843.3333	0.91016735
33.21103	459.66667	3819.6667	0.89258451
33.311063	412.33333	3579	0.89669283
33.411096	249	3201.3333	0.92783306
33.51113	120	3019	0.96177126
33.611163	47	3319.6667	0.9860396
33.711196	-60.333333	2982.6667	1.0206456
33.811229	-65.333333	3113.3333	1.0214348
33.911262	-64.333333	3178.6667	1.0206572
34.011296	50.666667	3282.6667	0.9848
34.111329	-57.666667	3520.3333	1.0166538
34.211362	139	3215	0.95855695
34.311395	155.66667	3454.3333	0.95687904
34.411429	128.66667	3373.3333	0.96325909
34.511462	21.666667	3698.3333	0.99417563
34.611495	-169.33333	3525	1.0504619
34.711528	-129	3153	1.0426587
34.811561	-241	2824.6667	1.0932783
34.911595	-54.666667	2814	1.0198115
35.011628	82.666667	3246.6667	0.9751702
35.111661	208	3262.3333	0.94006339
35.211694	264.33333	3209.6667	0.92391096
35.311728	169.66667	2836.3333	0.94355733
35.411761	103	2902.6667	0.9657314
35.511794	-22	3370	1.0065711
35.611827	204.66667	3614.6667	0.94641299
35.71186	205.33333	3426	0.94345511
35.811894	168.66667	3219.3333	0.95021645
35.911927	18	2887.3333	0.9938045
36.01196	6.3333333	3567	0.99822761
36.111993	165.66667	3445.6667	0.9541259
36.212027	343.33333	3483.6667	0.91028656
36.31206	361.66667	2899	0.88908199
36.412093	408.33333	2686.6667	0.86806677
36.512126	141	2895.3333	0.95356241
36.612159	69.333333	2782.6667	0.97568957
36.712193	-122.66667	3368	1.0377979
36.812226	24	3787.3333	0.99370299
36.912259	138	3848.3333	0.96538172
37.012292	83	3813.3333	0.97869792
37.112326	-16.666667	3557.3333	1.0047072
37.212359	23.666667	3818.6667	0.99384055
37.312392	215	3219.3333	0.93739687
37.412425	161.33333	3313.3333	0.95356869
37.512458	204.66667	3124.6667	0.93852623
37.612492	128.66667	3326	0.96275569
37.712525	372	3140.6667	0.89409755
37.812558	266.33333	2799.6667	0.91313329
37.912591	115.33333	2968.6667	0.96260268
38.012625	-26.333333	3006	1.0088377
38.112658	-103.33333	3449	1.0308857
38.212691	-125	3687	1.0350926
38.312724	-69.666667	3407.6667	1.0208708
38.412757	-8	3538	1.0022663
38.512791	220	3279.6667	0.93713687
38.612824	235	3444	0.93612395
38.712857	247	3071	0.92555756
38.81289	194	3171	0.9423477
38.912924	45	3704.6667	0.98799893
39.012957	-41	4017.6667	1.0103101
39.11299	-126.66667	3696.3333	1.0354842
39.213023	-201.33333	2961.3333	1.0729469
39.313056	-94.333333	3014	1.0323096
39.41309	39.666667	3213	0.98780488
39.513123	151.66667	3475.3333	0.95818399
39.613156	155.33333	3561.6667	0.95821003
39.713189	2.3333333	3912.6667	0.999404
39.813223	-171.66667	3472.3333	1.0520097
39.913256	-182	3392.6667	1.056686
40.013289	-107	3223.6667	1.0343316
40.113322	39	3251.6667	0.9881483
40.213355	188	3052.6667	0.94198725
40.313389	224.66667	2941.3333	0.92903769
40.413422	294	2850	0.90648855
40.513455	-1	3247	1.0003081
40.613488	19	3189	0.99407731
40.713522	27.333333	3736.6667	0.99273822
40.813555	378.66667	3117	0.89167541
40.913588	236.66667	2914.6667	0.92489951
41.013621	197.33333	2641.3333	0.9304838
41.113654	142	2920.3333	0.95363013
41.213688	278.33333	2959	0.91402389
41.313721	295.66667	3086.3333	0.91257638
41.413754	181.33333	3041.3333	0.9437319
41.513787	138.33333	3056	0.95669415
41.613821	135	2686	0.95214463
41.713854	-0.66666667	2674	1.0002494
41.813887	-16	2737.3333	1.0058795
41.91392	58.333333	3024.3333	0.98107699
42.013953	265	2708.3333	0.91087444
42.113987	484.66667	2228	0.82133202
42.21402	436.33333	1744	0.79987769
42.314053	610.66667	1771	0.74359692
42.414086	621	1913.6667	0.75499737
42.51412	761.66667	2142.3333	0.73771809
42.614153	798.33333	2263.6667	0.73927716
42.714186	859.33333	2283	0.72653018
42.814219	564.66667	2016.3333	0.78122175
42.914252	287	1845.6667	0.8654267

43.014267	122	4862.3333	0.97552331	43.014295	-43.333333	4474	1.0097803	43.014286	147.66667	1951	0.92963787
43.1143	208.33333	5154.3333	0.96115117	43.114328	54.666667	4114	0.98688629	43.114319	459.66667	2411	0.83987459
43.214333	263.33333	4847.6667	0.94847714	43.214362	38	3932.6667	0.99042982	43.214352	601	2766.6667	0.82153816
43.314366	372.66667	5491.3333	0.93644839	43.314395	-6	3916.6667	1.0015343	43.314385	540.66667	2673.3333	0.83177764
43.414399	510.66667	5250.6667	0.91136311	43.414428	-42.333333	4261.6667	1.0100332	43.414419	471.33333	2371	0.8341738
43.514433	651.66667	5059.6667	0.88589938	43.514461	23.333333	4555.3333	0.9949039	43.514452	512.66667	2120	0.80526716
43.614466	715.33333	4046	0.84976197	43.614495	81.333333	4636.6667	0.98276106	43.614485	528.33333	1839	0.77682343
43.714499	544.66667	4309.6667	0.88779784	43.714528	31.666667	4370	0.99280576	43.714518	558.66667	1919	0.77451904
43.814532	280.66667	4865.6667	0.94546279	43.814561	-70.333333	4136.3333	1.0172979	43.814551	545.66667	1733	0.76053248
43.914565	196	5273.3333	0.96416382	43.914594	-139.66667	4080.3333	1.0354424	43.914585	530.66667	1976	0.78829787
44.014599	356	5294.6667	0.93699858	44.014628	-244.66667	3974.3333	1.0656001	44.014618	481.33333	1955.6667	0.8024894
44.114632	299.66667	4917.3333	0.94255958	44.114661	-115.66667	3908.3333	1.0304975	44.114651	276.33333	2236.6667	0.89003847
44.214665	145	4811.3333	0.9707445	44.214694	-179.66667	4131.3333	1.045466	44.214684	185.33333	2363	0.92727273
44.314698	128.33333	4436.3333	0.9718855	44.314727	-56.333333	3967.3333	1.0144038	44.314718	70	2319	0.97069904
44.414731	283	3648	0.92800814	44.414761	-73	4579	1.0162006	44.414751	-74.333333	2274.3333	1.0337879
44.514764	362	3599.3333	0.90861663	44.514794	61	4502.3333	0.98663258	44.514784	200.33333	1949.6667	0.90682171
44.614798	494	3691	0.88195938	44.614827	121	4701	0.97490668	44.614817	144.66667	2205.3333	0.93843972
44.714831	797.33333	4291	0.84330167	44.71486	134	4204	0.96911019	44.71485	308.33333	2141	0.87411541
44.814864	1094.6667	4020.3333	0.78598892	44.814894	127	3740.3333	0.96716083	44.814884	65.333333	2500.3333	0.97453553
44.914897	873.33333	4540	0.83866995	44.914927	377	3837	0.91053631	44.914917	24.666667	2284.3333	0.98931716
45.01493	352.66667	4313.3333	0.92441777	45.01496	381	4054	0.91409245	45.01495	4.3333333	1936.6667	0.99776747
45.114964	110	4126.6667	0.97403619	45.114993	155.66667	4697.3333	0.96792362	45.114983	224.66667	2048.3333	0.90115853
45.214997	50.666667	4102	0.987799	45.215027	-149.33333	4679.6667	1.032963	45.215017	364	2100	0.85227273
45.31503	274.33333	4082.6667	0.93703619	45.31506	-234.66667	4871	1.0506147	45.31505	536.33333	2402.3333	0.81749093
45.415063	116	4473.3333	0.974724	45.415093	-135.66667	4219.3333	1.0332218	45.415083	423.33333	2119.3333	0.83350813
45.515096	162.66667	4476.6667	0.96493749	45.515126	-73	4168.6667	1.0178237	45.515116	480.33333	2219	0.82205483
45.615129	411.33333	4953	0.9233207	45.61516	17.666667	3715.6667	0.99526786	45.61515	304	2289	0.88276128
45.715163	554.33333	5485	0.90821283	45.715193	102.33333	4068.3333	0.97546355	45.715183	142.33333	2300	0.9417224
45.815196	597.66667	5495.3333	0.90190929	45.815226	-91.333333	3664.6667	1.0255597	45.815216	5	1900.6667	0.99737625
45.915229	305.66667	6143.3333	0.95260247	45.915259	-278	3798.6667	1.0789623	45.915249	313.33333	1754.3333	0.84846042
46.015262	268.66667	5699	0.95497961	46.015293	-405	3666.3333	1.1241823	46.015282	359.33333	1672.3333	0.82313372
46.115295	157	5567.6667	0.97257482	46.115326	-256	4223.6667	1.0645215	46.115316	639	1964.3333	0.75454545
46.215328	-69.333333	5151.6667	1.013642	46.215359	31.666667	4279	0.99265388	46.215349	506.66667	2021.6667	0.79960448
46.315362	-24	5368.6667	1.0044905	46.315392	43.333333	4199	0.9897855	46.315382	517	2140	0.80541965
46.415395	-22.666667	4916	1.0046322	46.415426	-69.666667	3863	1.0183656	46.415415	188.66667	2423	0.92776005
46.515428	112.33333	4563.3333	0.97597491	46.515459	-214.33333	3370.6667	1.0679058	46.515449	374.33333	2370.6667	0.86363084
46.615461	223.33333	4541.3333	0.95312719	46.615492	-247.66667	3589	1.0741221	46.615482	663	2443.6667	0.78658798
46.715494	166	5391.6667	0.97013135	46.715525	-208.66667	3511.3333	1.0631813	46.715515	874.66667	1866.6667	0.68093385
46.815528	121.66667	5052.3333	0.97648499	46.815559	-146.66667	4327	1.0350849	46.815548	390	1933.6667	0.83216181
46.915561	-169.66667	5378.3333	1.0325739	46.915592	52	4356.6667	0.98820505	46.915581	-59.333333	1825.6667	1.0335912
47.015594	12.666667	4969.6667	0.99745768	47.015625	80	4246.6667	0.98151002	47.015615	59	2405	0.97605519
47.115627	-40.333333	5707	1.0071176	47.115658	54.666667	3891	0.98614514	47.115648	285.33333	2428.6667	0.89486613
47.21566	311.33333	5448.3333	0.94594595	47.215691	52.666667	3774.3333	0.98623813	47.215681	680	2285	0.77065767
47.315693	241.66667	5369	0.95692728	47.315725	123.33333	3822.6667	0.96874472	47.315714	338.66667	2017.3333	0.85625354
47.415727	519.33333	5421	0.91257505	47.415758	78.666667	3725.3333	0.97932001	47.415748	503.66667	1769.6667	0.77844575
47.51576	728.66667	5424.3333	0.88157538	47.515791	84	4149.3333	0.98015748	47.515781	452	2017.3333	0.81695464
47.615793	622.33333	5336.6667	0.89556413	47.615824	43.666667	4147.3333	0.98958085	47.615814	751	1850.3333	0.7113019
47.715826	476.33333	4873.3333	0.91096018	47.715858	159	4127.3333	0.96290536	47.715847	448.33333	1948	0.81290861
47.815859	203	4761	0.95910556	47.815891	182.66667	3964.3333	0.95595209	47.81588	448.66667	1652	0.78641701
47.915893	394.33333	4845.3333	0.92474076	47.915924	-13	4269	1.0030545	47.915914	391	1733.6667	0.81597113
48.015926	438.66667	4938.6667	0.91842301	48.015957	-92.666667	4091.3333	1.0231744	48.015947	463.66667	1800.6667	0.79523038
48.115959	345.66667	4959.3333	0.93484134	48.115991	-267	3730	1.0771008	48.11598	340.66667	2048	0.85738208
48.215992	244	5310.3333	0.95607034	48.216024	-136.33333	3487	1.0406884	48.216013	244.33333	2223	0.90097271
48.316025	388.66667	5221.3333	0.93071895	48.316057	-113.33333	3441	1.0340579	48.316047	375	2274.6667	0.85847276
48.416058	331.66667	4891.3333	0.93649882	48.41609	-30	3500.3333	1.0086447	48.41608	542	2198	0.80218978
48.516092	389	4335.3333	0.91766034	48.516124	186.66667	3315	0.94669205	48.516113	620.33333	1975	0.76098125
48.616125	161.66667	4586.6667	0.96595297	48.616157	348	3589.3333	0.91161531	48.616146	412.33333	1884	0.82043838
48.716158	168.66667	4312.3333	0.96235959	48.71619	315.66667	3799	0.92328257	48.716179	338.33333	1881.6667	0.8475976
48.816191	143	4795.3333	0.97104286	48.816223	64	3943.6667	0.98403061	48.816213	536.66667	1898.6667	0.77963318
48.916224	338.66667	4792.6667	0.93400026	48.916257	-181.33333	3899.6667	1.0487674	48.916246	838	2112	0.7159322
49.016257	349.33333	5091.6667	0.93579612	49.01629	-295.66667	3796.3333	1.0844601	49.016279	774	2197.6667	0.7395401
49.116291	196.33333	5223.3333	0.96377391	49.116323	-182.33333	4147.6667	1.0459818	49.116312	410.66667	2849.3333	0.87402863
49.216324	-140.33333	4824.3333	1.0299601	49.216356	-188	4378	1.0448687	49.216346	386.33333	2848	0.88055241
49.316357	-92	5125.3333	1.0182781	49.31639	-193.33333	4241	1.0477641	49.316379	449	2820	0.86264913
49.41639	15	4901	0.99694874	49.416423	-318.66667	4215.6667	1.0817723	49.416412	790.33333	2211.6667	0.73673107
49.516423	417	5125	0.92475641	49.516456	-324.66667	3980	1.08882	49.516445	776.33333	1825.6667	0.70163976
49.616457	235	4898.3333	0.95422078	49.616489	102.66667	4126	0.97572127	49.616478	734.66667	1495	0.67050381
49.71649	283.33333	4705.6667	0.94320839	49.716523	300.66667	3849	0.92754438	49.716512	418	1362	0.76516854
49.816523	-48.666667	5491.3333	1.0089417	49.816556	355.33333	4001.3333	0.91843917	49.816545	259	1502.3333	0.85295231
49.916556	114.33333	5458.6667	0.97948442	49.916589	-7.6666667	4182.3333	1.0018365	49.916578	207	1820	0.89787864
50.016589	-140.66667	6177.6667	1.0233008	50.016622	-40.666667	4376.3333	1.0093796	50.016611	215	1991.3333	0.90255326
50.116622	133.33333	5613	0.9767968	50.116656	-41.666667	4050.3333	1.0103941	50.116645	172	2466.6667	0.93481556
50.216656	-81.333333	6034	1.0136633	50.216689	-58	3839.6667	1.0153372	50.216678	181.33333	2648.6667	0.93592462
50.316689	406.66667	5309.3333	0.92885468	50.316722	96	3609	0.97408907	50.316711	172.33333	2587.6667	0.93756039
50.416722	164.66667	5218.6667	0.96941176	50.416755	123	3921.3333	0.96958708	50.416744	223.66667	2008	0.89977595
50.516755	375	5415	0.93523316	50.516789	218.66667	3745.6667	0.9448415	50.516777	379	1732.6667	0.82052092
50.616788	96.666667	5322.6667	0.98216263	50.616822	68.666667	4031.6667	0.98325339	50.616811	438.66667	1875	0.81040196
50.716821	271	5429.6667	0.9524617	50.716855	30	4216.3333	0.99293508	50.716844	409.33333	2206	0.84348713
50.816855	435.66667	4798.3333	0.9167622	50.816888	-5	4606	1.0010867	50.816877	459	2094	0.82021152
50.916888	343.66667	5104.3333	0.93691875	50.916922	-13	4615.6667	1.0028244	50.91691	677.66667	2121	0.75786089
51.016921	318.66667	5147.6667	0.94170376	51.016955	179.33333	4525.6667	0.96188452	51.016944	810.66667	1975	0.70898648
51.116954	263.33333	5174.6667	0.95157533	51.116988	49.666667	4378.6667	0.98878434	51.116977	812	2052.6667	0.71654643
51.216987	600.66667	4606.6667	0.88464985	51.217021	108.66667	4429	0.9760523	51.21701	599	2042.3333	0.7732206
51.317021	492.66667	4616.3333	0.90356887	51.317055	-92.333333	4399.3333	1.021438	51.317043	454	1923.3333	0.80902973
51.417054	316.66667	4770.3333	0.93774982	51.417088	107	4901.3333	0.97863561	51.417076	358.66667	1874	0.83935503
51.517087	43.666667	5099.6667	0.99151005	51.517121	-64.333333	5057.6667	1.0128838	51.51711	527.66667	1672	0.76011517
51.61712	-40.333333	4733.6667	1.0085938	51.617154	132	5033.6667	0.97444667	51.617143	683	1601	0.70096322
51.717153	93	4970	0.98163144	51.717188	132.33333	4636.3333	0.97224941	51.717176	432.33333	1704.3333	0.79765991
51.817186	116.33333	5610.6667	0.97968686	51.817221	186.33333	4526.6667	0.96046396	51.817209	123.66667	1716.6667	0.93280203
51.91722	256.66667	5810.3333	0.95769463	51.917254	75	4331	0.98297776	51.917243	-6.3333333	2172	1.0029244
52.017253	264.66667	5748.3333	0.95598426	52.017287	-66.333333	4316.3333	1.0156078	52.017276	95.333333	2314.6667	0.9604426
52.117286	279.33333	5441.6667	0.95117404	52.11732	1.6666667	4345	0.99961656	52.117309	233.66667	2696.6667	0.92025936
52.217319	313.33333	5717	0.94804046	52.217354	91.333333	4509	0.98014637	52.217342	115	2700.3333	0.95915226
52.317352	370.66667	5457.3333	0.93639899	52.317387	156.66667	4411.3333	0.96570344	52.317375	-74.333333	3029	1.0251579
52.417386	665.66667	5504.6667	0.8921182	52.41742	232.33333	4755.3333	0.95341843	52.417409	-280.33333	2957.3333	1.1047192
52.517419	495	4825	0.90695489	52.517453	241.66667	4430	0.94826971	52.517442	-458.33333	2952	1.183799
52.617452	296.33333	4638.6667	0.93995272	52.617487	123	4112.3333	0.9709586	52.617475	-503.33333	2914.3333	1.2087654
52.717485	279.33333	4161.3333	0.93709653	52.71752	-40	3376.3333	1.0119892	52.717508	-351.66667	3152	1.1255803
52.817518	385.33333	4348.3333	0.91859728	52.817553	-253	3450.3333	1.0791284	52.817542	-225	3456	1.0696379
52.917551	358	4155.6667	0.92068533	52.917586	-275	3854.6667	1.0768228	52.917575	-97.666667	3391.3333	1.0296529
53.017585	86.333333	4261	0.98014108	53.01762	-163	4431	1.0381912	53.017608	102.33333	3067.3333	0.9677148
53.117618	49.666667	5127	0.99040567	53.117653	23.333333	4571	0.99492128	53.117641	168	2961	0.94630872
53.217651	94.333333	5261	0.98238516	53.217686	182.33333	4261.3333	0.95896782	53.217674	274	3370	0.9248079
53.317684	141.33333	5424.6667	0.97460774	53.317719	80.666667	4062.6667	0.98053097	53.317708	39	3727	0.98964418
53.417717	135.66667	4380.6667	0.96996088	53.417753	64.333333	3921.3333	0.98385883	53.417741	-146.33333	3597	1.0424073
53.51775	163.66667	5109.3333	0.96896138	53.517786	6	3967	0.99848981	53.517774	-137.66667	3588.6667	1.0398918
53.617784	449.33333	4622	0.9113974	53.617819	115.66667	3501	0.96801843	53.617807	-117.33333	3729.6667	1.0324813
53.717817	309.66667	5288.6667	0.94468592	53.717852	33	3512	0.99069111	53.717841	-13.333333	3854	1.0034716

53.81785	199	4839.6667	0.96050542	53.817886	-23	3650.3333	1.0063407	53.817874	-32.666667	3797.3333	1.0086772
53.917883	-124	5170.6667	1.0245707	53.917919	-113.33333	3489.6667	1.033567	53.917907	-28.333333	3154	1.0090647
54.017916	223.33333	4739	0.95499429	54.017952	-148	3603	1.0428365	54.01794	177	3210	0.94774136
54.11795	183.33333	4625	0.96187175	54.117985	-130.33333	3734.3333	1.0361635	54.117973	107.33333	3033.6667	0.96582829
54.217983	113.66667	4776.6667	0.97675687	54.218019	-200.33333	4186.6667	1.050255	54.218007	31	3291.3333	0.99066921
54.318016	-111.33333	5235	1.0217292	54.318052	-208.33333	4102.6667	1.0534965	54.31804	154.66667	2985	0.95073787
54.418049	-42.333333	5108.3333	1.0083564	54.418085	-90	3823	1.0241093	54.418073	262.66667	3235.6667	0.92491663
54.518082	100.66667	5410.6667	0.98173461	54.518118	-39	3515	1.0112198	54.518106	346.66667	3530	0.9105761
54.618115	346.66667	5089	0.93622371	54.618152	-90	3742.3333	1.0246418	54.61814	183.66667	3911	0.9551449
54.718149	340.33333	5334	0.94002232	54.718185	-126	3680.6667	1.0354464	54.718173	21	3914.3333	0.99466373
54.818182	122.66667	4961.3333	0.97587202	54.818218	-154	3892.3333	1.0411948	54.818206	13.666667	3740	0.99635912
54.918215	11	4937.6667	0.99777718	54.918251	-191.66667	3299.3333	1.0616754	54.918239	-28	4082.3333	1.0069062
55.018248	59	5225.6667	0.98883563	55.018285	-186.66667	3090.3333	1.0642865	55.018272	40.666667	3726.3333	0.9892045
55.118281	21	5081.6667	0.9958845	55.118318	-218.33333	2731	1.0868931	55.118306	156.66667	3392.6667	0.95586026
55.218315	-152.66667	4849	1.0325076	55.218351	-97.333333	3006.3333	1.0334594	55.218339	44	2924	0.9851752
55.318348	-133.33333	5044	1.0271518	55.318384	-166	2935.6667	1.059935	55.318372	3	2914.6667	0.99897178
55.418381	49	5295.6667	0.99083198	55.418418	-44.333333	3000.3333	1.0149977	55.418405	-128.33333	3267	1.0408879
55.518414	251.33333	5033.3333	0.95244102	55.518451	-110.66667	3148.3333	1.0364315	55.518439	-94.666667	3270.6667	1.0298069
55.618447	280.66667	5028.3333	0.9471338	55.618484	15.333333	3588.6667	0.99574547	55.618472	-21.666667	2995.3333	1.0072862
55.71848	218.33333	4893	0.95728447	55.718517	-110	3773	1.03003	55.718505	-75.666667	3110.6667	1.0249314
55.818514	137.66667	5460.3333	0.97540788	55.818551	-119.66667	3654	1.0338583	55.818538	-46.333333	2842.6667	1.0165693
55.918547	89.333333	5075.6667	0.9827041	55.918584	-284	3173.6667	1.0982812	55.918571	-107.33333	3076.3333	1.0361513
56.01858	127	5065	0.97553929	56.018617	-289.33333	2888.6667	1.1113106	56.018605	-61.666667	3054.3333	1.0206059
56.118613	55.333333	4788	0.98857536	56.11865	-371.66667	2852	1.1498455	56.118638	-48.666667	3217.6667	1.0153571
56.218646	39.666667	4569.6667	0.99139427	56.218684	-214.66667	3626.6667	1.0629152	56.218671	-107.66667	3332	1.0333919
56.318679	57	4501.6667	0.98749634	56.318717	-177.33333	3673.6667	1.0507198	56.318704	-135.33333	2979.3333	1.0475856
56.418713	466	4605.6667	0.90811699	56.41875	-16.333333	3593	1.0045666	56.418738	-224.33333	3319.6667	1.0724747
56.518746	599.66667	5243	0.89736422	56.518783	4	3316.6667	0.99879542	56.518771	-163.33333	3422.3333	1.0501176
56.618779	749.33333	5325	0.87663941	56.618816	60.666667	3537.6667	0.98314034	56.618804	-51.666667	3738.6667	1.0140132
56.718812	413	5439	0.92942584	56.71885	208.33333	3503.6667	0.94387572	56.718837	54.333333	3660	0.98537198
56.818845	419.33333	4703.6667	0.91814692	56.818883	212.33333	3154.6667	0.93693694	56.81887	293.33333	3473	0.922117
56.918879	469.33333	4400.6667	0.90362765	56.918916	395	3328.3333	0.89391226	56.918904	288.33333	3216.3333	0.91772874
57.018912	493	4208	0.8951287	57.018949	359	3429	0.90522703	57.018937	356	3189	0.89957687
57.118945	371.33333	4571.6667	0.92487693	57.118983	337.66667	3545	0.91303228	57.11897	172.33333	3167	0.94839289
57.218978	479	5012.3333	0.91277164	57.219016	183	3283	0.94720138	57.219003	10	3547.6667	0.99718917
57.319011	729	4981.6667	0.87234415	57.319049	-37.333333	3308.3333	1.0114134	57.319037	-127.33333	3120.3333	1.0425437
57.419044	849.66667	5703.3333	0.87033928	57.419082	-100	3349.3333	1.0307755	57.41907	-208.33333	2831.3333	1.0794256
57.519078	533.66667	5448	0.91078295	57.519116	-159.33333	3475.3333	1.0480499	57.519103	60	2685	0.97814208
57.619111	383	5835	0.93840463	57.619149	-109.66667	3533.3333	1.0320319	57.619136	16.333333	3483.3333	0.99533289
57.719144	215.66667	4976	0.95845907	57.719182	-218.33333	3203.3333	1.0731435	57.719169	206	3873.6667	0.94950568
57.819177	152.33333	5055.3333	0.97074826	57.819215	-222.33333	2960	1.0812127	57.819203	-19.333333	3968.6667	1.0048953
57.91921	-122.33333	4485	1.028041	57.919249	-36.333333	2902	1.0126788	57.919236	109.66667	3410.3333	0.9688447
58.019244	95.666667	4461	0.97900512	58.019282	2.6666667	3654.6667	0.99927087	58.019269	8.6666667	3248.6667	0.99733934
58.119277	235.33333	4979.6667	0.95487376	58.119315	39.333333	3853	0.98989466	58.119302	98.333333	3114.6667	0.96939517
58.21931	243.66667	5147	0.95479842	58.219348	-118.66667	3525.3333	1.0348337	58.219336	229	3336.6667	0.93577639
58.319343	441.33333	4963	0.91833714	58.319382	-106.33333	2772.6667	1.03988	58.319369	271.66667	3424.6667	0.92650374
58.419376	643.33333	4324.6667	0.87050456	58.419415	-109	2648.6667	1.042919	58.419402	89.666667	3497.3333	0.97500232
58.519409	772.66667	4990	0.86591856	58.519448	-133.33333	2671	1.0525417	58.519435	-135	3345	1.0420561
58.619443	449.33333	5502.3333	0.92450294	58.619481	-4	2940.3333	1.0013622	58.619468	-327	3648.3333	1.0984544
58.719476	445	6199.6667	0.933029	58.719515	-27.666667	3180	1.0087766	58.719502	-111.66667	3724.6667	1.0309069
58.819509	385	5541.6667	0.93503937	58.819548	24.666667	3772.6667	0.99350421	58.819535	-158.33333	3623.3333	1.045695
58.919542	544	4920.3333	0.90044531	58.919581	-38	3847.3333	1.0099755	58.919568	-101.66667	3350	1.0312981
59.019575	492.66667	4507	0.9014601	59.019614	-51	3447.6667	1.0150147	59.019601	-246	2994.6667	1.0894979
59.119608	592	5229.3333	0.89830508	59.119648	-83.666667	3236	1.0265412	59.119635	-174.33333	3361.6667	1.0546957
59.219642	481	5544.6667	0.92017481	59.219681	-139	3434	1.0421851	59.219668	-105.66667	3596.3333	1.0302712
59.319675	259.66667	4901.3333	0.94968675	59.319714	-75.333333	3626.3333	1.0212147	59.319701	0	3854.3333	1
59.419708	82.666667	4255.3333	0.9809436	59.419747	-15.666667	3511.3333	1.0044817	59.419734	52	3464	0.98521047
59.519741	5.6666667	4598.6667	0.99876928	59.519781	23.333333	3242.6667	0.99285568	59.519767	162.66667	2969.6667	0.94806853
59.619774	78	5176	0.98515417	59.619814	-4.6666667	2720	1.0017186	59.619801	291.66667	2994.6667	0.91124861
59.719808	38	5058	0.99254317	59.719847	-5	2497.3333	1.0020062	59.719834	480	3261.3333	0.87170349
59.819841	107.66667	4962.3333	0.97876397	59.81988	47.666667	2255.3333	0.97930236	59.819867	345.33333	3333.6667	0.90613391
59.919874	-87	4646.3333	1.0190817	59.919914	-27.666667	2587.6667	1.0108073	59.9199	73.333333	3403	0.97890498
60.019907	315.66667	5202	0.94278983	60.019947	-70.333333	3106.6667	1.0231639	60.019934	-246.33333	3288.3333	1.0809774
60.11994	225.33333	5433	0.96017673	60.11998	-105.33333	3573.6667	1.03037	60.119967	-259	3617.3333	1.0771216
60.219973	596.66667	6173.6667	0.91187042	60.220013	-55.666667	4162.6667	1.0135541	60.22	-286	3608	1.0860927
60.320007	359.66667	6025.6667	0.943673	60.320047	94.333333	3951.3333	0.97668287	60.320033	33.666667	3678.6667	0.99093113
60.42004	313.33333	6013.6667	0.95047679	60.42008	11	3559.3333	0.99691906	60.420066	-50.666667	3818	1.0134489
60.520073	204	6043.6667	0.96734781	60.520113	179.33333	3206.3333	0.9470316	60.5201	102.33333	3863	0.97419301
60.620106	258.66667	5882.6667	0.95788102	60.620146	67.333333	3220.3333	0.97951942	60.620133	-9	3971.3333	1.0022714
60.720139	748	5328.6667	0.8769062	60.72018	189.33333	3465	0.94818936	60.720166	153.33333	3707.3333	0.9602832
60.820173	632	4810.3333	0.88387334	60.820213	93.666667	3187.3333	0.97145179	60.820199	-31.333333	3446.3333	1.0091752
60.920206	539.66667	4989.6667	0.90239932	60.920246	56.666667	2872.6667	0.98065544	60.920233	-208.66667	3060.6667	1.073165
61.020239	77.666667	5383	0.98577707	61.020279	-77.333333	2654	1.0300129	61.020266	-287.33333	3211.3333	1.0982672
61.120272	-114.33333	5889	1.0197991	61.120313	-230.66667	3166	1.0785828	61.120299	-241	3296.3333	1.0788785
61.220305	245.66667	5611	0.9580535	61.220346	-254	3254.6667	1.0846479	61.220332	-174.66667	3098.3333	1.0597423
61.320338	573	5166	0.90015682	61.320379	-200.66667	3490.3333	1.0609991	61.320365	-75	2648.6667	1.0291413
61.420372	714.33333	4677.3333	0.86751159	61.420412	6.3333333	3265.3333	0.99806419	61.420399	89	2729.6667	0.96842479
61.520405	372.33333	4837	0.92852572	61.520445	-110	3035.6667	1.0375983	61.520432	63.666667	2971.6667	0.97902482
61.620438	205.33333	4862	0.95947902	61.620479	-46	2988.3333	1.0156339	61.620465	54.666667	2862	0.98125714
61.720471	409	5042.3333	0.92497248	61.720512	-250.33333	2742.3333	1.1004548	61.720498	202	2811	0.93295719
61.820504	489.33333	4864.3333	0.90859847	61.820545	-45	3130.6667	1.0145836	61.820532	289.66667	3109	0.9147705
61.920537	435.33333	4798.6667	0.91682588	61.920578	8	3135	0.99745466	61.920565	376.66667	3380	0.89973381
62.020571	249	4204.3333	0.94408683	62.020612	267.66667	3266.3333	0.92425957	62.020598	212	3266	0.93904543
62.120604	431.66667	4109.3333	0.90494017	62.120645	169	3474.3333	0.95361391	62.120631	211	2955	0.93335439
62.220637	378.33333	3924	0.91206322	62.220678	107.33333	3664.3333	0.9715422	62.220664	-0.33333333	2910.6667	1.0001145
62.32067	463.33333	4092	0.89828772	62.320711	-103.66667	3437.3333	1.0310969	62.320698	-16.666667	2846	1.0058907
62.420703	238.66667	4096	0.94494002	62.420745	23.333333	3034	0.99236808	62.420731	-34.333333	2772	1.0125411
62.520737	338.33333	4514.3333	0.93027888	62.520778	269	2650.3333	0.90785567	62.520764	8.3333333	2740.6667	0.99696859
62.62077	518.33333	4665	0.9	62.620811	368	3372.3333	0.90161305	62.620797	-35.666667	2933.6667	1.0123073
62.720803	721.33333	4513.3333	0.86220071	62.720844	257.33333	3606.6667	0.93340235	62.720831	144.33333	3126.3333	0.95587036
62.820836	836.66667	4321	0.83778194	62.820878	-85.333333	3638.6667	1.024015	62.820864	94	3635.6667	0.97479668
62.920869	784	4598	0.85432925	62.920911	-115.33333	3693.3333	1.032234	62.920897	26.333333	3424.6667	0.99236936
63.020902	615.66667	4469.3333	0.87892494	63.020944	27.333333	3643.3333	0.99255358	63.02093	-131.33333	3172.6667	1.0431828
63.120936	482.33333	4673.6667	0.90645203	63.120977	64	3814.6667	0.98349948	63.120963	-101	2798.6667	1.0374398
63.220969	297.33333	5130.3333	0.94521894	63.221011	-9	3305.3333	1.0027303	63.220997	85.333333	2723	0.96961424
63.321002	363	5746.6667	0.94058596	63.321044	-58.666667	3154.3333	1.0189512	63.32103	55.666667	2708.3333	0.97986011
63.421035	168.33333	5768.3333	0.97164514	63.421077	-31.333333	3364.3333	1.0094009	63.421063	112	2852.6667	0.96222172
63.521068	377.66667	5304.6667	0.93353669	63.52111	-36.666667	2903	1.0127922	63.521096	-18.333333	3136.3333	1.0058798
63.621102	167.33333	4651.3333	0.96527393	63.621144	-175.66667	3400	1.0544815	63.62113	-35	3229.6667	1.0109558
63.721135	182.33333	4863	0.96386099	63.721177	-196.33333	3189.6667	1.0655902	63.721163	-207	3149	1.0703603
63.821168	-177.66667	4980.6667	1.0369908	63.82121	-103.33333	3986.6667	1.0266094	63.821196	-14.666667	3200.3333	1.004604
63.921201	40.333333	5566	0.99280576	63.921243	17	3700.6667	0.99542724	63.921229	-149.66667	3256	1.0481811
64.021234	176	5458.3333	0.96876294	64.021277	-127	3534.3333	1.0372725	64.021262	-59.666667	3248.6667	1.0187101
64.121267	389.66667	5162.6667	0.9298193	64.12131	-269.66667	3315.3333	1.0885411	64.121296	219.33333	2012.3333	0.9017177
64.221301	243.66667	4850.3333	0.95216595	64.221343	-380.33333	3394.3333	1.1261889	64.221329	1233.6667	1021	0.45283856
64.321334	183.33333	4881	0.96379912	64.321376	-196	3197.3333	1.0653043	64.321362	2061.6667	-56.333333	-0.028091755
64.421367	127.33333	4630	0.9732343	64.42141	-110.33333	2683.3333	1.0428812	64.421395	2796.3333	44	0.01549114
64.5214	57.666667	4361.6667	0.98695127	64.521443	-27	2480.6667	1.0110039	64.521429	2899.3333	-12	-0.004156084

64.621433	82.333333	4585.3333	0.98236092
64.721466	267.33333	4742.6667	0.94664005
64.8215	538.33333	4679.3333	0.89682489
64.921533	884.33333	4120	0.82328649
65.021566	705	4254	0.85783424
65.121599	581.33333	4380.3333	0.88283507
65.221632	340.66667	4490.6667	0.92948806
65.321666	235.66667	4155.6667	0.94633369
65.421699	587	4588.3333	0.88657735
65.521732	535.66667	4580.3333	0.8952958
65.621765	645.33333	5123	0.88812482
65.721798	85	5109	0.98363496
65.821831	56	5511	0.98994072
65.921865	-1.6666667	5729.6667	1.000291
66.021898	239.33333	6157.3333	0.96258468
66.121931	534.33333	6016	0.91842654
66.221964	847.66667	5361	0.86347042
66.321997	671	5520.3333	0.8916227
66.422031	365	5098.3333	0.93319097
66.522064	72	5265.6667	0.98651096
66.622097	441.33333	4989.6667	0.91873811
66.72213	353	4863.3333	0.93232794
66.822163	538.66667	5099.6667	0.90446349
66.922196	400.66667	5524.6667	0.93238074
67.02223	384	5843	0.93833307
67.122263	104.33333	5653.6667	0.98188028
67.222296	232	4703	0.95298886
67.322329	415.66667	4657.3333	0.91806295
67.422362	559	4711.3333	0.8939346
67.522395	102.33333	5087.3333	0.98028133
67.622429	-79.666667	4697	1.0172538
67.722462	-54	4840	1.0112829
67.822495	232.66667	4606.6667	0.95192175
67.922528	331	5023.3333	0.93818091
68.022561	483.33333	4517	0.90333978
68.122595	592	4385	0.88105284
68.222628	457.33333	4188	0.90154994
68.322661	475.33333	4309.3333	0.90065487
68.422694	374.33333	4417	0.92187283
68.522727	413	4732	0.91972789
68.62276	316.33333	5180	0.94244648
68.722794	93.666667	5557.6667	0.98342574
68.822827	397	5519.6667	0.93290141
68.92286	610	5774.3333	0.90445361
69.022893	703	5944.6667	0.89424861
69.122926	330	5659.3333	0.94490205
69.22296	-95	5383	1.0179652
69.322993	11.666667	5123.3333	0.99772801
69.423026	212.33333	5183.3333	0.96064743
69.523059	353.66667	4900	0.93268194
69.623092	124	5131.6667	0.97640642
69.723125	62	5413.3333	0.98867649
69.823159	-22.333333	5270.3333	1.0042556
69.923192	130	4627.6667	0.97267568
70.023225	78.666667	5013.3333	0.98455093
70.123258	105	5156.3333	0.98004308
70.223291	-0.66666667	5868.3333	1.0001136
70.323324	293.33333	6154.6667	0.95450786
70.423358	457	6257.6667	0.93194003
70.523391	479	6445.6667	0.930827
70.623424	258.33333	5148.3333	0.95221948
70.723457	222.66667	5508.3333	0.96114698
70.82349	263	4737	0.9474
70.923524	228.33333	5460.3333	0.95986171
71.023557	47	5164	0.99098062
71.12359	128	5428.3333	0.97696323
71.223623	22	4884.3333	0.995516
71.323656	128	5041	0.97523699
71.423689	323.33333	4869.6667	0.9377367
71.523723	465.33333	4756.6667	0.91088983
71.623756	592.33333	4002	0.87107306
71.723789	301.66667	3913.3333	0.92843021
71.823822	484	4575.3333	0.90433522
71.923855	548.66667	5103.6667	0.90293094
72.023889	458	4847.3333	0.91367178
72.123922	156	4165.3333	0.96390003
72.223955	128	4061.3333	0.96944621
72.323988	170.33333	4965.3333	0.96683326
72.424021	491.33333	5645.6667	0.91993917
72.524054	2371.3333	3919.3333	0.62303942
72.624088	3763.6667	1974.3333	0.3440804
72.724121	5606	157.33333	0.027299017
72.824154	4967.3333	241.33333	0.046333035
72.924187	5218	270.33333	0.049255998
73.02422	5149.3333	169.33333	0.031837553
73.124253	4837.3333	527.66667	0.098353526
73.224287	4186	614	0.12791667
73.32432	4059.3333	801.66667	0.16491806
73.424353	4670.3333	728	0.13485644
73.524386	5220.3333	417.66667	0.074080643
73.624419	5327.3333	219	0.039485546
73.724453	5061	-86.333333	-0.017354597
73.824486	5060	-170.66667	-0.034905918
73.924519	4772	-178	-0.038746191
74.024552	4801.6667	-55	-0.011587079
74.124585	4886	-114.33333	-0.02396088
74.224618	4618.6667	-102	-0.022583026
74.324652	5145.6667	-136.33333	-0.027215864
74.424685	5661	-171	-0.031147541
74.524718	5628	-255.66667	-0.047589502
74.624751	4990.6667	-283	-0.060114707
74.724784	4589.3333	-176.33333	-0.039957701
74.824818	4529.3333	32.666667	0.007160602
74.924851	4588	148	0.03125
75.024884	4925.3333	88.666667	0.017683819
75.124917	5556.6667	-64.333333	-0.011713297
75.22495	5531.3333	-182	-0.034022931
75.324983	5352	78	0.014364641

64.621476	34.333333	2543	0.98667874
64.721509	86.333333	2605.6667	0.96792967
64.821543	32	2775	0.98859993
64.921576	-49.666667	3148	1.0160301
65.021609	-44.333333	3335	1.0134724
65.121642	114.66667	3195.6667	0.96536099
65.221676	59.666667	2975	0.98033831
65.321709	90	3344	0.9737915
65.421742	-141.33333	3420	1.043107
65.521775	-104	3638	1.0294284
65.621809	-59	3115.3333	1.0193042
65.721842	20.666667	3198.6667	0.99358045
65.821875	197.66667	3145.6667	0.94087737
65.921908	-34	3409.6667	1.0100721
66.021941	1.6666667	3642.3333	0.99954263
66.121975	-111.66667	3523.3333	1.0327308
66.222008	1.3333333	3273.3333	0.99959283
66.322041	291.66667	2961.6667	0.91034836
66.422074	223	2971.3333	0.93018888
66.522108	132.66667	3573.6667	0.96420541
66.622141	-40.666667	3548.3333	1.0115937
66.722174	111	3721	0.9710334
66.822207	217.33333	3191.3333	0.93624095
66.922241	113.33333	3074	0.96444259
67.022274	-17	2730.6667	1.0062646
67.122307	-193	3005	1.0686344
67.22234	-264.33333	3309.6667	1.0867995
67.322374	-283.33333	3410	1.0906183
67.422407	-21	3516.3333	1.006008
67.52244	-46.666667	3371	1.0140379
67.622473	129.66667	3244	0.96156506
67.722507	28	2456.3333	0.98872937
67.82254	135	2066.6667	0.93868282
67.922573	-26	2309.6667	1.0113852
68.022606	-97	3159.6667	1.0316717
68.12264	-97.333333	3331.3333	1.0300969
68.222673	9.3333333	3505.3333	0.99734446
68.322706	113.33333	3406.3333	0.96779998
68.422739	138	3690.6667	0.96395612
68.522773	21	3541.6667	0.99410554
68.622806	15.666667	3981.6667	0.99608072
68.722839	114.66667	3912	0.97152318
68.822872	265.33333	4129	0.93961921
68.922906	138.33333	3353	0.96037808
69.022939	-72.333333	3254.3333	1.022732
69.122972	-216.66667	3022.6667	1.0772155
69.223005	-65.666667	3198	1.0209641
69.323039	472.66667	2851.6667	0.8578161
69.423072	1611.6667	1937.6667	0.54592412
69.523105	2646.6667	1051	0.2842333
69.623138	3407	328.33333	0.08789934
69.723172	3203	147	0.043880597
69.823205	3426.3333	97.333333	0.027622741
69.923238	3309.6667	137.66667	0.039934249
70.023271	3590.6667	19.666667	0.005447327
70.123305	3764.6667	-189	-0.052857276
70.223338	3836.3333	-216.33333	-0.059760589
70.323371	3559.3333	-139.33333	-0.040740741
70.423404	3528	130	0.035538546
70.523438	3233	112.33333	0.033579115
70.623471	3676.6667	222.66667	0.057103778
70.723504	3226	40	0.012247397
70.823537	3473.3333	297.66667	0.078935738
70.92357	3127	376.33333	0.1074215
71.023604	3651.3333	418.66667	0.1028665
71.123637	3716.3333	161.66667	0.041688155
71.22367	3766.6667	53	0.013875556
71.323703	3185	69.333333	0.021304927
71.423737	3227.6667	102.33333	0.030730731
71.52377	3010.6667	4	0.001326847
71.623803	3196.6667	-38.333333	-0.012137203
71.723836	2911.3333	-87.333333	-0.030925401
71.82387	3050.3333	-50.333333	-0.016777778
71.923903	3070.3333	-60	-0.019931348
72.023936	3296.6667	101.66667	0.029916626
72.123969	3490	269.33333	0.071643908
72.224003	3831	207.66667	0.05141961
72.324036	3456.6667	64.333333	0.018271324
72.424069	3279	-46	-0.014228271
72.524102	2843.6667	62	0.021337616
72.624136	3323.6667	119	0.034566228
72.724169	3643.6667	-49.666667	-0.013819329
72.824202	3762.6667	-141.33333	-0.039027982
72.924235	3412.6667	-290	-0.092869342
73.024269	3341	-190.33333	-0.060410495
73.124302	3204	-43.666667	-0.013817108
73.224335	3403	139	0.039243365
73.324368	3536.6667	204	0.054535733
73.424402	3456.6667	70.333333	0.019941404
73.524435	3402.3333	-71	-0.021312788
73.624468	3194.3333	-58.333333	-0.01860119
73.724501	3104.3333	-39	-0.012722923
73.824535	2969	-55.333333	-0.018990962
73.924568	3202.6667	-43.333333	-0.013715974
74.024601	3517	-27.666667	-0.007928926
74.124634	4065.6667	67.666667	0.016370968
74.224668	3888.3333	4	0.001027661
74.324701	3462.6667	92.333333	0.025972808
74.424734	2577	191	0.06900289
74.524767	2563	184.33333	0.067095365
74.624801	2693	123.33333	0.043792165
74.724834	3070.3333	19	0.006150194
74.824867	3487.6667	2.6666667	0.000764015
74.9249	3995.6667	-30	-0.007564932
75.024934	4163	0	0
75.124967	3770.3333	-47.666667	-0.012804441
75.225	3729.6667	-71.666667	-0.019591762
75.325033	3534.3333	-140	-0.041245213

64.621462	2843.6667	-35.666667	-0.012701804
64.721495	2682.6667	-55.666667	-0.021190204
64.821528	2764.6667	-79	-0.029415415
64.921561	3119.3333	14	0.004468085
65.021595	3196.6667	-68.666667	-0.021952259
65.121628	3205.3333	-0.66666667	-0.00020803
65.221661	3316	-100.66667	-0.031308314
65.321694	3004.6667	10.333333	0.003427308
65.421728	2762.3333	-59.666667	-0.022076961
65.521761	2327.6667	-103	-0.046299071
65.621794	2053.3333	-103.33333	-0.052991453
65.721827	2056	-92	-0.046843177
65.82186	2223	-54.666667	-0.025211376
65.921894	2572.3333	-97.333333	-0.039326599
66.021927	2833.3333	-102	-0.037344398
66.12196	2758.3333	-66	-0.024514052
66.221993	2785	-79	-0.029194383
66.322027	2569.3333	1	0.000389055
66.42206	2709	11	0.004044118
66.522093	2988	46	0.015161503
66.622126	2882.6667	125	0.041560457
66.722159	2838.3333	52	0.017991004
66.822193	2635.6667	57.666667	0.021410891
66.922226	3050.3333	-18.333333	-0.006046614
67.022259	2808.3333	14	0.004960435
67.122292	3222	-77	-0.024483307
67.222326	2604	-158.66667	-0.064885496
67.322359	2635	-99.333333	-0.039174445
67.422392	2167.6667	-127.66667	-0.062581699
67.522425	2377	-100	-0.043917435
67.622458	2356.6667	-212	-0.09884986
67.722492	2520.3333	-175.33333	-0.074769012
67.822525	2896.6667	-30.333333	-0.010582626
67.922558	3004.6667	15.666667	0.005187065
68.022591	3137.3333	25	0.007905555
68.122625	2987.6667	-57	-0.0194495
68.222658	3228.3333	52	0.015852048
68.322691	2850.6667	122	0.041040592
68.422724	2676.6667	-17	-0.006391778
68.522757	2477	-14	-0.005684125
68.622791	2981	-50.666667	-0.017290411
68.722824	3335.6667	107.66667	0.031268151
68.822857	3325.6667	-46	-0.014025816
68.92289	3087.6667	-15	-0.004881753
69.022924	2944	-111.66667	-0.03942568
69.122957	2703	15.666667	0.005762629
69.22299	2526.3333	74.333333	0.028582415
69.323023	2390.3333	181.66667	0.070632452
69.423056	3031.6667	59.666667	0.019301272
69.52309	3467.3333	70.333333	0.019881278
69.623123	3379.6667	47	0.013715953
69.723156	3379.3333	-25.333333	-0.00755317
69.823189	3088	-30.333333	-0.009920419
69.923223	3857.6667	-65.333333	-0.01722774
70.023256	3901.6667	151	0.037259418

75.425017	5265.3333	73.666667	0.01379784
75.52505	5459.3333	63.333333	0.01146789
75.625083	5420.3333	257.33333	0.045323783
75.725116	4999	660.66667	0.11673243
75.825149	5220.3333	583.66667	0.10056283
75.925182	5140	64.666667	0.012424747
76.025216	5507.3333	-88.333333	-0.01630067
76.125249	5210	8	0.001533155
76.225282	4827.6667	80.666667	0.016434635
76.325315	4823	-106	-0.02247191
76.425348	5040.3333	41	0.008068748
76.525382	5155.6667	82.666667	0.015781101
76.625415	4836.3333	69.333333	0.014133315
76.725448	4472.3333	-155.33333	-0.035981777
76.825481	4169	-123	-0.030400395
76.925514	3774.6667	-12.666667	-0.003367003
77.025547	3792	0	0
77.125581	3716	202.66667	0.051718272
77.225614	4661	210	0.043112297
77.325647	5314.3333	393.66667	0.068967531
77.42568	6254	75	0.011850213
77.525713	5721.6667	-38	-0.006685825
77.625747	5225.6667	-113.66667	-0.022235263
77.72578	4282.6667	28.666667	0.006649142
77.825813	4678.6667	16	0.003408123
77.925846	4549.3333	-52	-0.011562407
78.025879	5063.3333	142	0.027279713
78.125912	5318.6667	59	0.010971301
78.225946	5617.3333	-45	-0.008075612
78.325979	4881	-325	-0.071334504
78.426012	4233.3333	-306	-0.077915464
78.526045	3928	-134.33333	-0.035409894
78.626078	4894.6667	360.66667	0.068628695
78.726111	4960.6667	473.66667	0.087161872
78.826145	5057	409	0.074826198
78.926178	4574.3333	-36.666667	-0.008080511
79.026211	4877	-16	-0.003291504
79.126244	4797.6667	-42	-0.008831569
79.226277	4583.3333	-75	-0.01663586
79.326311	4421.3333	-179.33333	-0.042275656
79.426344	4840.3333	-274.66667	-0.060159159
79.526377	5669.3333	-64.333333	-0.011477847
79.62641	5382	-100.66667	-0.019060843
79.726443	4968.3333	105	0.020696452
79.826476	4360.3333	-145.66667	-0.034561848
79.92651	4696	-3	-0.00063925
80.026543	5416	-134	-0.025369178

75.425066	3127.6667	-168.33333	-0.056882181
75.5251	2917	-51.333333	-0.017913226
75.625133	3297.6667	44	0.013167082
75.725166	3315.6667	106.33333	0.031073446
75.825199	3111.3333	74.666667	0.023435865
75.925233	3004.6667	-82	-0.028056569
76.025266	3333	-63.333333	-0.019369966
76.125299	3691	-156.66667	-0.044327077
76.225332	3921.6667	71	0.017782601
76.325366	3805.6667	74	0.019073804
76.425399	3642	128.66667	0.034123055
76.525432	3333	-35.333333	-0.010714647
76.625465	3192.3333	-50.666667	-0.016127321
76.725499	3135	-188	-0.063793688
76.825532	3013.6667	-157.33333	-0.055082273
76.925565	2682.3333	-97.333333	-0.037653127
77.025598	2688	-56.666667	-0.021535343
77.125632	2666	-11	-0.004143126
77.225665	3306.6667	-124.66667	-0.039178714
77.325698	3249.3333	62.666667	0.018921095
77.425731	2990.6667	-63.666667	-0.021751509
77.525765	2839.3333	-91.666667	-0.03336164
77.625798	2724.3333	-74.333333	-0.028050314
77.725831	3039	12	0.003933137
77.825864	2954	216	0.068138801
77.925898	3400.3333	90.666667	0.025971546
78.025931	3795.6667	67	0.01734553
78.125964	3988.6667	-13	-0.003269892
78.225997	4032	40	0.009823183
78.326031	3904.3333	40	0.010141131
78.426064	3839	117.66667	0.029738837
78.526097	3568.3333	330.66667	0.08480807
78.62613	3333.3333	348.66667	0.094694912
78.726164	3122.6667	398.33333	0.11313074
78.826197	3421.6667	205.66667	0.056699136
78.92623	3534.6667	116	0.031775018
79.026263	3218.3333	-98.333333	-0.031517094
79.126297	2655	-192.33333	-0.078099621
79.22633	2352	-165	-0.075445816
79.326363	3039	-182	-0.063703185
79.426396	3288.6667	-241	-0.079076889
79.52643	3273.6667	-186.66667	-0.060468632
79.626463	2946.3333	-171.33333	-0.061741742
79.726496	3117	-173.66667	-0.059003398
79.826529	3041.6667	-281.33333	-0.10192006
79.926563	3326.3333	-51	-0.015570934
80.026596	3031	-49	-0.016431925
80.126629	3116.3333	-57	-0.01863151
80.226662	3100.3333	-275.33333	-0.097463127
80.326695	3305.3333	44.666667	0.013333333
80.426729	3639.3333	249	0.06403772
80.526762	3578	358.66667	0.091109229
80.626795	3422	28	0.008115942
80.726828	3441	-194.33333	-0.059856263
80.826862	3375	-341	-0.11239288
80.926895	3419	-293.33333	-0.093846646

file:///Users/costaa/Dropbox%20(The%20Francis%20Crick)/Marcus's%20Nucleosome%20Intasome%20manuscript/Final%20Submission/source%20file%20(SM%20data)%20190715_final.htm[06/08/2019, 22:13:44]
